# *Gatad2b*, associated with the neurodevelopmental syndrome GAND, plays a critical role in neurodevelopment and cortical patterning

**DOI:** 10.1038/s41398-023-02678-x

**Published:** 2024-01-18

**Authors:** Clemer Abad, Maria C. Robayo, Maria del Mar Muñiz-Moreno, Maria T. Bernardi, Maria G. Otero, Christina Kosanovic, Anthony J. Griswold, Tyler Mark Pierson, Katherina Walz, Juan I. Young

**Affiliations:** 1 John P. Hussman Institute for Human Genomics, Miller School of Medicine, University of Miami, Miami, FL USA; 2 IQUIBICEN - CONICET, School of Exact and Natural Sciences - University of Buenos Aires, Buenos Aires, Argentina; 3 The Board of Governors Regenerative Medicine Institute, Cedars Sinai Medical Center, Los Angeles, CA USA; 4 Dr. John T. Macdonald Foundation Department of Human Genetics, Miller School of Medicine, University of Miami, Miami, FL USA; 5 Guerin Children’s, Departments of Pediatrics, Cedars Sinai Medical Center, Los Angeles, CA USA; 6 Department of Neurology, Cedars Sinai Medical Center, Los Angeles, CA USA; 7 The Center for the Undiagnosed Patient, Cedars Sinai Medical Center, Los Angeles, CA USA; 8 Present Address: KU Leuven Department of Neurosciences, Leuven Brain Institute, Leuven, Belgium

**Keywords:** Epigenetics and behaviour, Molecular neuroscience

## Abstract

*GATAD2B* (GATA zinc finger domain containing 2B) variants are associated with the neurodevelopmental syndrome GAND, characterized by intellectual disability (ID), infantile hypotonia, apraxia of speech, epilepsy, macrocephaly and distinct facial features. *GATAD2B* encodes for a subunit of the Nucleosome Remodeling and Histone Deacetylase (NuRD) complex. NuRD controls transcriptional programs critical for proper neurodevelopment by coupling histone deacetylase with ATP-dependent chromatin remodeling activity. To study mechanisms of pathogenesis for GAND, we characterized a mouse model harboring an inactivating mutation in *Gatad2b*. Homozygous *Gatad2b* mutants die perinatally, while haploinsufficient *Gatad2b* mice exhibit behavioral abnormalities resembling the clinical features of GAND patients. We also observed abnormal cortical patterning, and cellular proportions and cell-specific alterations in the developmental transcriptome in these mice. scRNAseq of embryonic cortex indicated misexpression of genes key for corticogenesis and associated with neurodevelopmental syndromes such as *Bcl11b*, *Nfia* and *H3f3b* and *Sox5*. These data suggest a crucial role for *Gatad2b* in brain development.

## Introduction

Neurodevelopmental disorders (NDDs), affecting brain development and function are estimated to affect ∼2–5% of children (Deciphering Developmental Disorders Study [1]). Genetic research has identified a large number of genetic mutations associated with NDDs. In spite of the genetic heterogeneity underlying the wide clinical spectrum of NDDs, there is a significant convergence of genetic pathways involving chromatin remodeling [2–4]. Chromatin remodeling complexes are central to the transcriptional control of cells, regulating the local chromatin state, yielding either transcriptional activation or repression. Understanding how mutations in chromatin remodeling genes affect transcriptional regulation during brain development may reveal developmental and cellular mechanisms driving NDDs and help identify the therapeutic targets.

Recently, mutations in *GATAD2B* were identified in patients with GAND (*GATAD2B*-associated neurodevelopmental disorder), a NDD characterized by intellectual disability (ID), infantile hypotonia, apraxia of speech, epilepsy. These cortical issues are associated with strabismus, macrocephaly (without megalencephaly) and characteristic facial features [5–8]. *GATAD2B* (initially called p66β) is a member of NuRD (Nucleosome Remodeling and histone Deacetylation), a multi-subunit chromatin-remodeling complex that contains at least two enzymatic functions: ATP-dependent chromatin remodeling and histone deacetylation. Chromatin remodeling activity is provided by either CHD3, CHD4 or CHD5 and histone deacetylase by HDAC1 or HDAC2. Other core constituents of NuRD include methyl-CpG-binding domain (MBD) containing proteins MBD2/3, histone chaperones RbAp46/48, DNA-binding proteins MTA1/2/3 and GATAD2B or its paralog GATAD2A [9, 10]. NuRD complexes have been implicated in regulating gene transcription, genome stability and DNA repair [11–14], as well as controlling events related to cell cycle progression, stem cell differentiation, and cerebral corticogenesis [15, 16].The CHD paralogs are sequentially expressed in different cell types during corticogenesis and were reported to play individualized roles in regulating the proliferation of progenitors (CHD4), neuronal migration (CHD5) and cortical layer specification (CHD3) [7, 17]. Depletion of the NuRD complex in postmitotic neurons impairs synapse development [18], consistent with altered synapse development in flies with neuronal knockdown of the fly GATAD2A/B single orthologue [19]. Further, previous work evaluating mice with *Mbd3* deletion (Mbd3 -/-) showed early embryological demise, while conditional Mbd3 null mice had defects in neurogenesis, a reduction in cortical thickness, and neonatal lethality [20, 21]. These findings are consistent with the identification of genetic variation in NuRD subunits, including *CHD3*, *CHD4*, *CHD5*, *GATAD2A*, and *GATAD2B* in neurodevelopmental disorders such as ID, autism spectrum disorders, schizophrenia and global developmental delay [22, 23].

GATAD2B, is one of the least studied members of the NuRD complex, and therefore its relevance to brain development and physiology, although evidenced by the association with GAND, is still largely unknown. The majority of *GATAD2B* mutations associated with GAND are predicted to be loss of function, suggesting that this syndrome involves haploinsufficiency of GATAD2B. To study pathogenesis mechanisms associated with *GATAD2B* haploinsufficiency and delineate GATAD2B functions, we characterized a mouse model harboring an inactivating mutation in *Gatad2b*. Mice with mutant *Gatad2b* exhibited behavioral and learning abnormalities resembling the human phenotype. These mice also had abnormal cortical development and gene expression indicating the intellectual disability associated with *GATAD2B* mutations results from abnormal epigenetic transcriptional regulation of corticogenesis.

## Materials and methods

### Animals

*Gatad2b*^tm1a(EUCOMM)Hmgu^, MGI:4434913, hereafter *Gatad2b*^stop^ were obtained from the European Mouse Mutant Archive EMMA. The colony was expanded at the University of Miami. The genetic background of the mice was C57BL/6N-A^tm1Brd^. Since *Gatad2b*^stop/+^ dams were not good mothers, litters derived from *Gatad2b*^+/+^ males x *Gatad2b*^stop/+^ were nursed by wild type foster females. At weaning age animals were housed 2-4 per cage in a room with a 12-hour light/dark cycle with access to food and water *ad libitum*. For behavioral and histological studies, animals from each genotype were randomly selected. All procedures were approved by the University of Miami Institutional Animal Care and followed the NIH Guidelines, “Using Animals in Intramural Research”.

### *Gatad2b* mRNA expression

**Fig. 1:**
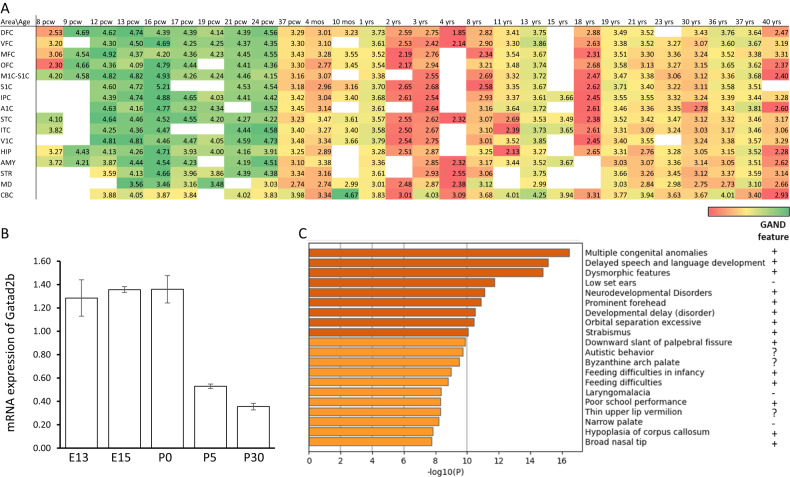
Developmental expression of *GATAD2B* and gene enrichment analysis suggest GATAD2B has a critical neurodevelopmental role. **A** Expression level of human *GATAD2B* measured in RPKM (reads per kilobase of exon model per million mapped reads) from postmortem human brain specimens at indicated developmental stages profiled by RNA sequencing (RNA-Seq) and exon microarray hybridization. Data extracted from BrainSpan Atlas of the Developing Human Brain (http://brainspan.org.) (Allen Institute for Brain Science et al. 2023). DFC dorsolateral prefrontal cortex, VFC ventrolateral prefrontal cortex, MFC medial prefrontal cortex, OFC orbital prefrontal cortex, M1C: primary motor cortex, S1C: primary somatosensory cortex, IPC posteroinferior parietal cortex, A1C primary auditory cortex, STC superior temporal cortex, ITC inferior temporal cortex, V1C primary visual (occipital) cortex, HIP: hippocampus, AMY amygdaloid complex, STR Striatum, MD mediodorsal nucleus of thalamus, CBC cerebellar cortex, pcw postconception weeks. White rectangles: no data. **B** Expression of mouse *Gatad2b* by qRT-PCR at indicated time points during development. **C** DisGeNET enrichment analysis. Phenotypic features reported as present in GAND are marked with a plus sign, question marks denote uncertainty.

**Fig. 2:**
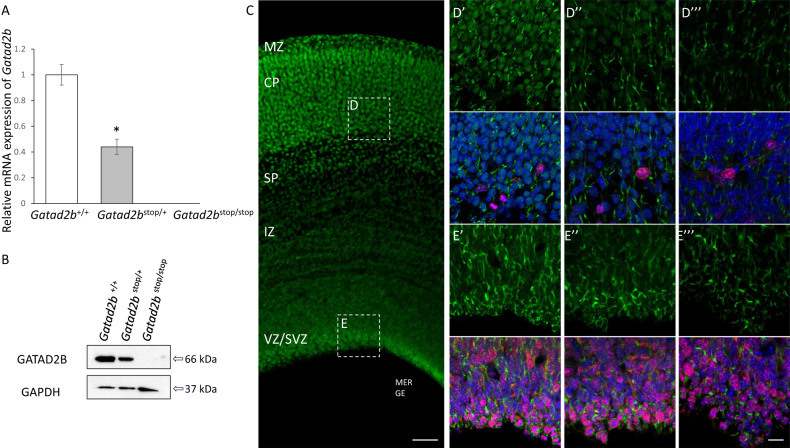
*Gatad2b*^*stop/+*^ mice are haploinsufficient. **A** Expression of *Gatad2b* mRNA as determined by qRT-PCR in brains of 1day old mice (*n* = 4 of each genotype, *p < 0.05). **B** Western blot for the detection of Gatad2b in cerebellum samples from WT (*Gatad2b*^*+/+*^), heterozygous (*Gatad2b*^*stop/+*^) and homozygous (*Gatad2b*^*stop/stop*^) mice. **C** Immunostaining for Gatad2b in 40 μm sections derived from *Gatad2b*^+/+^ E16.5 brain. Scale bar: 60 μm. Boxes **D** and **E** depict the CP and VZ/SVZ areas shown in **D’**, **E”’**, where immunostaining for Gatad2b (green) and Ki67 (red) is shown for *Gatad2b*^+/+^ (**D’**, **E’**), *Gatad2b*^*stop/+*^ (**D”**, **E”**) and *Gatad2b*^*stop/stop*^ (**D”’**, **E”**’) E 16.5 brain samples. DAPI (blue) was used as DNA marker to identify nuclei and showed merged with Gatad2b (green) and KI67 (red). Scale bar: 15 μm MZ marginal zone, CP cortical plate, SP subplate, IZ intermediate zone, VZ/SVZ ventricular zone and subventricular zone.

Quantitative PCR was used to assess the expression level of *Gatad2b* in brain of day 1 old mice. RNA was isolated from the homogenized brain tissue using TRIzol (Invitrogen). Five hundred nanogram of RNA were used to synthetize cDNA using qScript XLT Supermix kit (Quanta) following the manufacturer’s protocol. Quantitative real time RT-PCR (RT-qPCR) was performed on an Applied Biosystems QuantStudio 12 K Flex using SYBR Green (Applied Biosystems). The primers used for detecting *Gatad2b* transcript were forward 5’ -AGGCCTCATGGAGACAACAA- 3’ and reverse 5’ –GTTAGCCTTCCTCGGTCTGG- 3’. Results were normalized against Gapdh (5’ -CAACTTTGTCAAGCTCATTTCCTG- 3’ and reverse 5’– TCAGTGTCCTTGCTGGGGTG- 3’). All reactions were performed in triplicate with the following amplification protocol: 10 min at 95 °C, and 40 cycles of 10 s at 95 °C, 30 s at 58 °C, and 30 s at 72 °C. The relative expression level of *Gatad2b* in the brain was calculated with the ΔCt method using a Gapdh as a housekeeping gene. Figure 1B contains data from a single experiment with 1 or 2 animals per time point (sex of embryos was not determined, postnatal mice were males). Figure 2A contains data from two experiments with 4 animals per genotype.

### Western blot

The complete cerebellum of E18 embryos was homogenized in RIPA buffer in the presence of protease inhibitors. Samples were then centrifuged at 14,400 rpm for 30 minutes at 4 °C. Proteins were resolved using precast SDS/PAGE gels (Invitrogen, Carlsbad, CA) under reducing conditions and transferred to a nitrocellulose membrane using the Turbo-trans Blot system (Biorad, USA). The membrane was incubated in 5% BSA for 1.5 hr and incubated overnight at 4 °C in primary antibody diluted 1:1000 (anti-Gatad2b (A301-2882A, Bethyl), in TBST (TBS+Tween 0.5%). Blots were washed with TBST and incubated in HRP-conjugated Goat secondary antibodies (1:3000) for 1.5 hr at room temperature. On termination of antibody reactions, blots were washed three times with TBST and developed and visualized using west pico super-signal ECL substrate (Pierce, Thermo, USA) and FluorChemE (ProteinSimple, USA), respectively. Stripping Buffer (Thermo Fisher Scientific, USA) was used to wash off bands, then blocked for 1 h and incubating the same membrane with primary antibody Gapdh (Cell Signaling Technology, USA); then incubated with HRP-conjugated secondary antibody goat anti-rabbit (Cell Signaling Technology, USA). Figure 2B contains data from a single western blot on cell extracts from one cerebellum per genotype.

### Immunofluorescence analysis

**Fig. 3:**
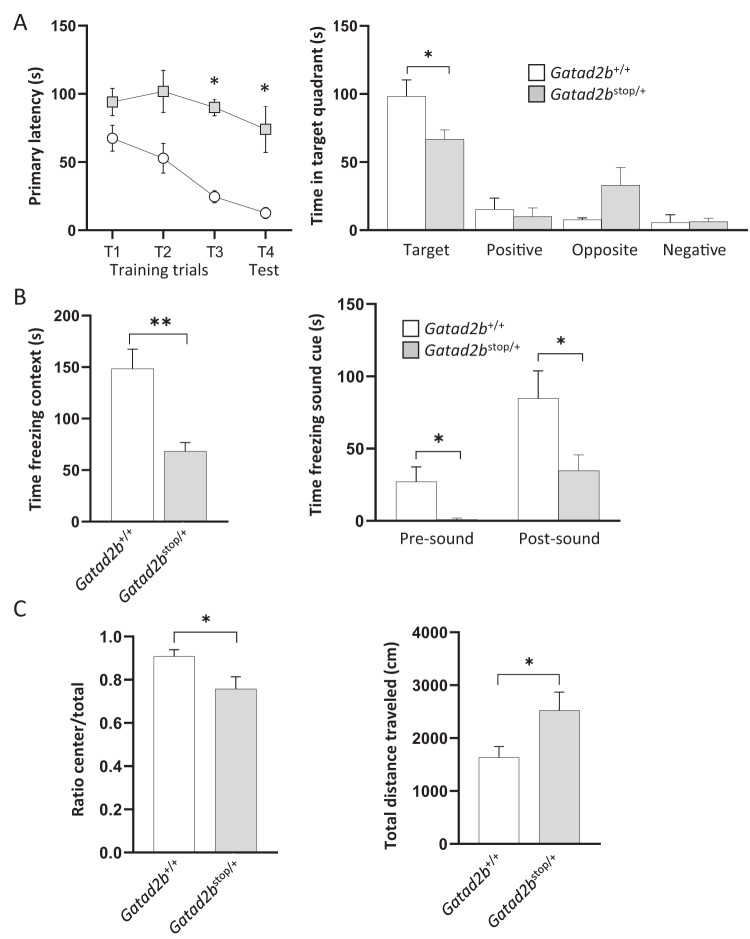
*Gatad2b* haploinsufficient mice exhibit behavioral abnormalities. **A** Spatial learning and memory was assessed by the Barnes maze task at 3 months of age. The time taken to identify the target hole (primary latency) is shown in the left panel for both the training phase as well as for the probe test day in which the escape box was removed. The distribution of time during the probe test, performed 1 day after the last training session, is shown in the right panel. Two-way repeated measures ANOVA showed main effects of day (F3,20 = 46.43, *P* < 0.0001) and genotype (F1,20 = 8.3, *P* = 0.0148), indicating that *Gatad2b*^stop/+^ mice performed worse than the wild type at finding the target hole across the acquisition phase. In the probe test, *Gatad2b*^stop/+^ exhibited differences in exploratory activity. *p < 0.05, *post hoc* comparisons using Tukey test. **B** Learning and memory of contextual fear was assessed by fear conditioning. Left: Contextual test: freezing responses on the contextual testing 24 h after conditioning. There was significantly less freezing in *Gatad2b*^stop/+^ mice during contextual testing (**p < 0.01, unpaired Student’s t-test). Right: Cued test: freezing responses on the cued testing. There was significant difference between WT and *Gatad2b*^stop/+^ mice in cued conditioning during pre-tone and tone (*p < 0.05, unpaired Student’s t-test). **C** Activity levels were measured in the open field test. Left: analysis of the ratio of distance traveled in the center of the open field to total distance traveled suggest abnormal anxiety-related responses in *Gatad2b*^stop/+^ mice (*<p0.05, unpaired Student’s t-test). Right: total distance traveled showed novel environment-induced hyperactivity in *Gatad2b*^stop/+^ mice (*p < 0.05, unpaired Student’s t-test). *N* = 9 male mice per genotype. Bars represent mean ± S.E.M.

The brains were dissected and immediately fixed in 4% of paraformaldehyde (PFA) at 4 °C overnight. After fixation, the brains were rinsed with cold PBS and stored at 4 °C until further analysis. Coronal sections were obtained using a vibratome (Leica biosystems) and permeabilized with 0.3% Triton X-100 and blocked in 5% BSA for one hour at room temperature, followed by overnight incubation at 4 °C with primary antibodies. A rabbit anti-Gatad2b (A301-2882A, Bethyl), mouse anti-Satb2 (ab32036, Abcam), rat anti-Ctip2 (ab18465, Abcam), rabbit anti-Tbr1 (ab31940, Abcam), mouse anti-2H3 (2H3-s, DSHB), mouse anti-Tuj1 (201201, Biolegend), mouse anti-NeuN (MAB377, Millipore Sigma) and rabbit anti-GFAP (ab7260, Abcam) were utilized as a primary antibody for *Gatad2b*^*+/+*^, *Gatad2b*^*stop/+*^ and *Gatad2*^*stop/stop*^ mice. Sections were washed with PBS, and respective secondary antibodies were applied. DAPI (Calbiochem) was used to counterstain nuclear DNA. Specimens were washed with PBS and mounted in fluorescence mounting medium (Dako). Images were taken using a Zeiss LSM710 confocal microscope. Figure 2C shows representative data from an experiment performed two times with a minimum of two independent embryos (sex undetermined) of each genotype. Figure 3 shows representative data from two experiments that included 3 embryos (sex undetermined) of each genotype. To estimate the number of callosal axons (Fig. 5), we quantified the area covered by the anti-filament (2H3) signal in coronal sections in a space of 200 × 200 μm placed on immunostaining images in the cingulate cortex. For each genotype, at least two embryonic brains (undetermined sex) were analyzed.

### Behavioral characterization

At 12 weeks of age, male mice were randomly selected (*n* = 9 of each genotype) and subjected to a battery of tests with 0-2 days between tests in the following order: (i) Barnes maze, (ii) Open field, and (iii) Pavlovian conditioned fear. For each test, the number of mice (n) tested is indicated in the respective figure caption. Although testing was meant to be performed by an investigator blind to the genotype, the presence of craniofacial abnormalities was suggestive of genotype.

#### Barnes maze test

The Barnes maze task was conducted on a circular PVC platform (100 cm in diameter) with 18 holes (hole diameter: 5.5 cm) along the perimeter. A black plexiglass escape box was located under one of the holes, the hole above the escape box represented the target. The test consisted of 5 consecutive days. Days 1-4 were for habituation (pre-training trial) and reference memory (T1-T3 training trials). On day five, the probe trial was performed. On the habituation trial, mice were placed in the center of the maze covered by a black cylinder; after 10 sec mice were released, guided to the escape box and allowed to remain there for 2 min. Following the pre-training trial, the training trials started. At the beginning of each trial, mice were placed in the same position (center of the platform) and covered with the black cylinder for 10 sec followed by release and allowing them to explore the maze. The trial ended when the mice enter the escape box or after 3 min. The animal was allowed to stay in the box for 1 min. Mice underwent four trials per day for 4 days. On day 5, a probe test was conducted without the escape box, to assess memory based on distal environmental room cues. The latency and path to reach the target hole were recorded.

#### Open field

Mice were placed in the center of a clear plexiglass (40 × 40 × 30 cm) open field arena and allowed to explore for 30 min. Exploratory behavior and general activity in the open filed is quantitated by a computer-operated activity monitor software (DOC-102, Med Associates).

#### Pavlovian conditioned fear

Mouse was first placed into a chamber for 5 min (pre-context) to allow exploration. The fear-conditioning training consisted of a 3 min acclimation period followed by an 80 dB (2.8 kHz) white noise that was presented for 30 sec as a conditional stimulus (CS) followed by a mild foot-shock (2 sec, 0.75 mA), which served as the unconditioned stimulus (US). After 2 min another CS-US pair was presented. Two minutes later the animal is placed in their home cage. After 24 h, mice were placed again in the same chamber to test their contextual memory ability, which consist of exploration for 5 minutes in the same chamber that was used the day before. Cue testing is performed after one hour of the context testing, mice were put in the chamber but the environmental cue, contextual cue, and odor were changed; for this purpose, black triangular plexiglass was inserted to alter the shape and spatial cues, red house lights replaced the white house lights, the wire grid floor was covered with white plexiglass, and 5% acetic acid was used to alter the smell. Two phases of the CS test were recorded, in the first phase (pre-sound cue) freezing time was recorded for 3 min, then a 30-sec tone without foot-shock was presented, finally, immediately after the tone occurred, the second phase for another 3 min was recorded to evaluate the mouse memory ability (post-sound). Mouse freeze software (Medpc-IV, Med Associates) was used to analyze the freezing time during the test.

### Pathway enrichment analysis

Gene Ontology (GO) enrichment analysis was performed using the Metascape platform [24] and DisGeNET database, which integrates standardizing data of disease-associated genes from multiple sources [25], through the EnrichR software [26]. In Metascape, we used the Membership (that flag genes which fall under GO biological process terms that include a preselected keyword and calculates enrichment significance) and Enrichment (identify unbiased pathways with significant enrichment *p* values, and automatically cluster them to reduce redundancy) functions.

### Isolation of embryonic cortex and single-cell dissociation

Timed-pregnant females were sacrificed to collect embryos (E16.5). Brains were collected from the embryos (*n* = 3 for WT and KO, *n* = 4 for Het), then the cortex was carefully removed using a stereo microscope from each embryo and placed in a 15 ml tube containing Hibernate E/B27/GlutaMax (HEB) media (BrainBits). Tissue pieces from the cortex were then incubated in a Papain solution (BrainBits) at 37 °C for 20 minutes. Following this, the Papain solution was gently removed and replaced with the HEB media. The tissues were manually disrupted using a polished Pasteur pipette for a maximum of 10 times. After disruption we allowed the tissue debris to settle for 1 minute at room temperature, the supernatant was transferred to a new tube and centrifuged at 200 rcf for 2 minutes to collect the cells. The supernatant was then carefully removed, and the cell pellet was resuspended in 1 ml of pre-warmed neuronal culture medium (NbActiv1). Subsequently the cell suspension was passed through a 30 µM cell strainer (Miltenyi Biotech) and an aliquot was used for counting using the Automated cell counter (Invitrogen) and morphological evaluation. Single-cell suspensions containing more than 90% viable cells were used for the generation of single-cell RNA sequencing libraries. Fast genotyping (~1 h) was performed with tissue from the rest of the body using Platinum Direct PCR Universal Master Mix.

### Single-cell RNA library preparation and sequencing

Single-cell RNA-seq libraries were generated using the 10X Genomics platform (Chromium Single Cell 3′ library and Gel Bead Kit v3, PN-1000075) and sequenced on a NovaSeq 6000 system (Illumina) at the Center for Genome Technology at the HIHG. Cells at a concentration of 1200 cell/mL were loaded on the 10X Genomics Chromium platform to isolate ~7,000 cells per sample and create individually barcoded Gel bead-in-Emulsions (GEMs) which were processed using the Chromium Single Cell 3’ Reagent Version 3 Kit. Sequencing libraries were evaluated for quality on the Agilent Tape Station (Agilent Technologies, Palo Alto, CA, USA), and quantified using a Qubit 2.0 Fluorometer (Invitrogen, Carlsbad, CA) and qPCR before sequencing on the Illumina NovaSeq 6000 targeting 100,000 reads per cell with sequencing parameters: Read1, 28 cycles; Index1, 8 cycles; Read2, 98 cycles.

### Processing of RNA sequencing data

Fastq files were aligned to the mm10 mouse reference genome and count matrices were generated using the CellRanger (v2.1) pipeline. Except where otherwise specified, we processed and visualized the scRNA-seq counts with the following Seurat-based pipeline, using Seurat v3.0.2 70. We filtered out cells with less than 1000 unique molecular identifiers (UMIs) based on the inflection point of the log-transformed barcode rank plot of each sample, or more than 10% of the UMIs coming from mitochondrially encoded genes. In total, 70,514 cells passed these filters, with a median of 7011 UMIs/cell, 2917 genes/cell, and 5.2% of UMIs in each cell coming from mitochondrially encoded genes. We next scaled and centered the UMI counts and used the default vst method to identify the top 2,000 variable genes. We removed sex specific genes (*Xist, Tsix, Eif2s3y, Ddx3y, Uty, and Kdm5d*), erythrocyte marker genes (*Hba-a1, Hba-a2, Hbb-b1, Hbb-bh1, Hbb-bh2, Hbb-bs, Hbb-bt, Hbb-y, Hbq1a, Hbq1b*), and *GM42418* and *AY636118*, mapping to rDNA repeating units, from the expression matrix to reduce the likelihood of false positives.

Data integration was performed to identify shared cell states across different samples following a recently published Seurat protocol [27]. Similar cells were clustered using an initial resolution in Seurat of 0.4 (27 clusters) through a final resolution of 0.4 resulting in 20 distinct clusters. Each cluster was assigned to a cell type on the basis of expression of marker genes based on expression defined by published single-cell transcriptome analysis of mouse embryonic brain [28–31]. To compute differentially expressed genes (DEGs) within each cluster we used the MAST test, which employs a generalized linear model framework using cell detection rate across groups as a co-variate [32]. The MAST test has low error and false discovery rates in comparison with other single-cell differential expression methods [33].

### Statistical analysis

Power analysis based on our previous studies on anticipated differences in the behavioral phenotypic variables indicate that at least 7 animals per group were necessary to detect a 20% change in mean with a type 1 error probability of 0.05 and 80% power. In this study 9 males/genotype were analyzed to account for casual losses. Inclusion/exclusion criteria for behavioral experiments were pre-established. Some criteria for exclusion were the presence of seizures during the testing, inability to walk in the open field, no reaction to the foot-shock in the Pavlovian conditioned fear. In our study no animal needed to be excluded from the analysis. Before statistical analysis the behavioral datasets were tested for normal distribution and analysis of the variance. For all, normal distribution and similar variance between the statistically compared groups was found. Behavioral data analysis was performed utilizing two-way repeated measures ANOVA, Tukey test and two-tailed Student’s t-test. Viability data as well as dysmorphology data were analyzed utilizing the Chi square statistic test. *P* value ≤ 0.05 was considered statistically significant.

## Results

### *GATAD2B* expression is higher in embryonic development and correlates with expression of genes involved in epigenetic and neurodevelopmental processes

Data from the publicly available dataset BrainSpan, which profiles the human brain transcriptome across the lifespan (8 postconceptional weeks (pcw) to 40 years old), show that *GATAD2B* expression is higher during embryonic development than in postnatal stages (http://www.brainspan.org, Fig. 1A). *GATAD2B* expression drops after 24 weeks pcw, except in the cerebellum, where *GATAD2B* seems to be expressed persistently until adulthood at similar levels. Higher expression of *Gatad2b* during development was confirmed in mice. We determined by RT-qPCR that mouse *Gatad2b* has higher expression in the brain during embryonic development than in postnatal ages (Fig. 1B), suggesting that *GATAD2B* has a role in neurodevelopmental processes.

A search for genes with a developmental expression pattern similar to human *GATAD2B* (i.e. *GATAD2B* used as “seed gene”) using the “Find Correlates” function of BrainSpan revealed 111 unique genes with a Pearson’s correlation > 0.85 (Supplementary Table 1). Gene ontology enrichment analysis indicates that the two most significantly overrepresented biological processes in this *GATAD2B* developmentally correlated gene set are related to “chromatin remodeling” (comprising GO:0016569, “covalent chromatin modification” multi-test adjusted *p* = 3.9E-11; GO: 0016573, “histone acetylation” multi-test adjusted *p* = 1.1E-4, GO:0006338, “chromatin remodeling” multitest adjusted *p* = 1.2E-3 and GO:0010452, “histone H3-K36 methylation” multi-test adjusted *p* = 1.0E-2) and “brain development” (GO: 0007420, multi-test adjusted *p* = 0.024) (Supplementary Table 2). Using the Disease Gene Network (DisGeNET) database -a comprehensive catalogue of human-disease associated genes-, significant enrichment in this *GATAD2B* neurodevelopmentally correlated gene list was observed in neurodevelopmental disease terms including features that have been reported in GAND patients (Fig. 1C).

### Validation of *Gatad2b* mutant mouse model

To understand the relationship between *GATAD2B* and neurodevelopment we evaluated a mutant mouse strain generated by the International Mouse Genotyping Consortium (IMPC), available through the European Mouse Mutant Archive (EMMA). These mice (*Gatad2b*^tm1a(EUCOMM)Hmgu^, MGI:4434913, hereafter *Gatad2b*^stop^) carry an insertion of a trapping cassette between *Gatad2b* first and second coding exons (intron 3). The intronic insertion is expected to “trap” the splicing of coding exon 1 to produce a LacZ fusion transcript and truncate the *Gatad2b* wild-type transcript. Since exons are retained in the insertional mutant allele, unintended expression of wild-type mRNA could potentially result from splicing skipping the trapping cassette (supplemental Fig. 1). We thus evaluated the expression of *Gatad2b* in brain tissue from heterozygous *Gatad2b*^stop/+^ mice by RT-qPCR. This analysis indicated that *Gatad2b*^stop/+^ express ~50% of *Gatad2b*, as compared to wild-type mice (Fig. 2A). Protein expression of Gatad2b followed mRNA; western analysis in E18 cerebellum showed approximately 50% of Gatad2b content in *Gatad2b*^stop/+^ mice and was undetectable in *Gatad2b*^stop/stop^ mice (Fig. 2B). Immunostaining of forebrain sections derived from embryonic day 16.5 (E16.5) *Gatad2b* mutant embryos confirmed the western data. Reduced and absent expression of *Gatad2b* was observed in the nuclei of cells in the cortex of *Gatad2b*^stop/+^ and *Gatad2b*^stop/stop^ embryos, respectively (Fig. 2C–E). Immunoreactivity of *Gatad2b* in wild-type (WT) brains showed nuclear localization throughout the cortex with higher levels in postmitotic cells of the cortical plate and the subplate than in proliferative cells in the deeper layers (see amplified boxes in Fig. 2C). Thus, we demonstrate that *Gatad2b*^stop*/+*^ mice have substantially reduced expression of *Gatad2b* mRNA and protein, supporting the use of this transgenic animal as a model of *Gatad2b* haploinsufficiency.

### Mice harboring heterozygous germline *Gatad2b* mutation exhibit a cognitive deficit

*Gatad2b*^stop/+^ mice are fertile and survive to adulthood, whereas *Gatad2b*^stop/stop^ mice die perinatally. From a total of 169 pups born from *Gatad2b*^stop/+^ x *Gatad2b*^stop/+^ matings, 55 were identified as wild type, 103 as heterozygous and only 11 were *Gatad2b*^stop/stop^ homozygous. These *Gatad2b*^stop/stop^ died soon after birth without overt morphological defects.

We performed a phenotypic analysis of *Gatad2b*^stop/+^ mice assaying features related to the clinical presentation of GAND subjects. *Gatad2b*^stop/+^ mice had a significantly broader snout than their wild-type siblings (Supplementary Fig. 2A), suggesting partial recapitulation of the craniofacial phenotype of GAND patients. Further, 85% of *Gatad2b*^stop/+^ mice exhibited increased curvature of the nasion vs. 14% of *Gatad2b*^+/+^ mice (*p* value = 0.018, Supplementary Fig. 2B). Spontaneous seizures were not observed in these mice at any age, suggesting that this phenotype, present in 24% of GAND subjects, is absent in this mouse model. Also, no significant difference was detected in the grip strength test and the hanging test performed at one month of age (data not shown), despite 100% GAND patients displaying infantile hypotonia [5]. We assessed cognitive functions in *Gatad2b*^stop/+^ mice at 12 weeks of age by the Barnes maze test, a contextual fear-conditioning test, and a cued fear-conditioning test. In the Barnes maze, *Gatad2b*^stop/+^ mice took significantly longer than the *Gatad2b*^+/+^ littermates to find the hidden escape hatch at test day (Fig. 3A). The analysis showed that at the beginning of training (days one and two), the latency to find the hidden target was not different of *Gatad2b*^stop/+^ and wild type mice, suggesting that non-cognitive deficits are not involved in the observed difference in the third and fourth probe tests. The increased latency to find the target implies that *Gatad2b* haploinsufficiency affects learning and memory processes. We thus investigated the performance of *Gatad2b* haploinsufficient mutants in a contextual fear conditioning task. *Gatad2b*^stop/+^ mice showed reduced levels of freezing compared with wild-type mice further suggesting cognitive alterations (Fig. 3B).

Although anxiety-like behavior has not been reported to be a common finding in GAND subjects, when introduced in the novel environment of the open field, *Gatad2b*^stop/+^ mice spent significantly more time exploring the periphery while avoiding the anxiety-provoking center (Fig. 3C, left panel). *Gatad2b*^stop/+^ mutant mice also exhibited significantly increased activity in this test (Fig. 3C, right panel).

### Haploinsufficiency of *GATAD2B* is associated with abnormal cortical development

**Fig. 4:**
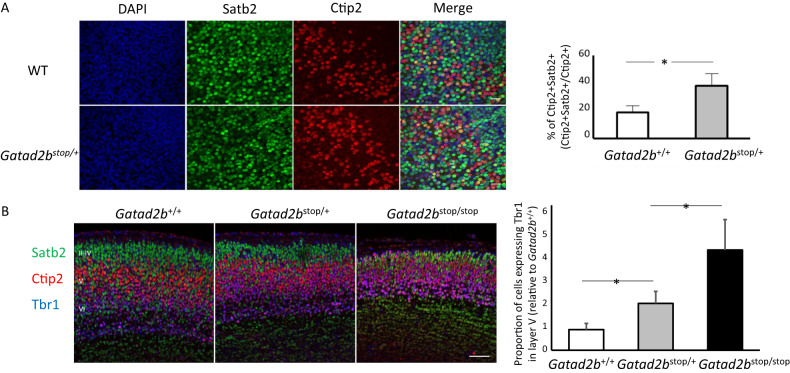
*Gatad2b* deficiency disrupts cortical neuronal development. **A** Immunostaining of coronal sections of E16.5 brains (cortex) with antibodies against Satb2 (green), and Ctip2 (red). Scale bar, 60 μm. Quantification data obtained with ImageJ (*n* = 3 of each genotype) for the proportion of double Satb2+ Ctip2+ neurons is shown to the right of the image **B** Coronal sections from E18.5 cortices immunolabeled for Satb2 (green), Ctip2 (red) and Tbr1 (blue). Scale bar: 100 μm. The relative proportion of Tbr1+ cells (*n* = 3 of each genotype) in layer V is depicted on the right. *P < 0.05. Data are presented as the mean ± S.E.M. *p ≤ 0.05.

To start dissecting the putative role of *Gatad2b* in cortical development, we analyzed the expression of cortical laminar markers in the brains of *Gatad2b* mutant mice. Immunostaining of embryonic brain sections showed reduction of cortical thickness and abnormal density of layer-specific neurons in both *Gatad2b*^stop/+^ and *Gatad2b*^stop/stop^ embryos (Fig. 4). Interestingly, although we observed that the expression pattern of the neuronal markers Ctip2 and Satb2 was grossly normal in *Gatad2b*^stop/+^ embryos (Satb2 was highly expressed in layers II-IV and less expressed in deep layers and Ctip2 was highly expressed in neurons of layer V and weakly expressed in layer VI) suggesting that cortical lamination in *Gatad2b* haploinsufficient mice is not overly deranged, we also observed that the frequency of cells co-expressing Satb2 and Ctip2 was significantly higher in the cortex of *Gatad2b*^stop/+^ embryos at E16.5 than in control mice (Fig. 4A). Further, although the deep layer marker Tbr1 is highly expressed in layer VI in all genotypes, the proportion of cells expressing Tbr1 in layer V (co-expressing Ctip2) is significantly higher in *Gatad2b*^stop/+^ and *Gatad2b*^stop/stop^ E18.5 embryos (Fig. 4B). These data indicate that the cognitive phenotype of these Gatad2b deficient mice is accompanied by alterations in cortical development.

**Fig. 5:**
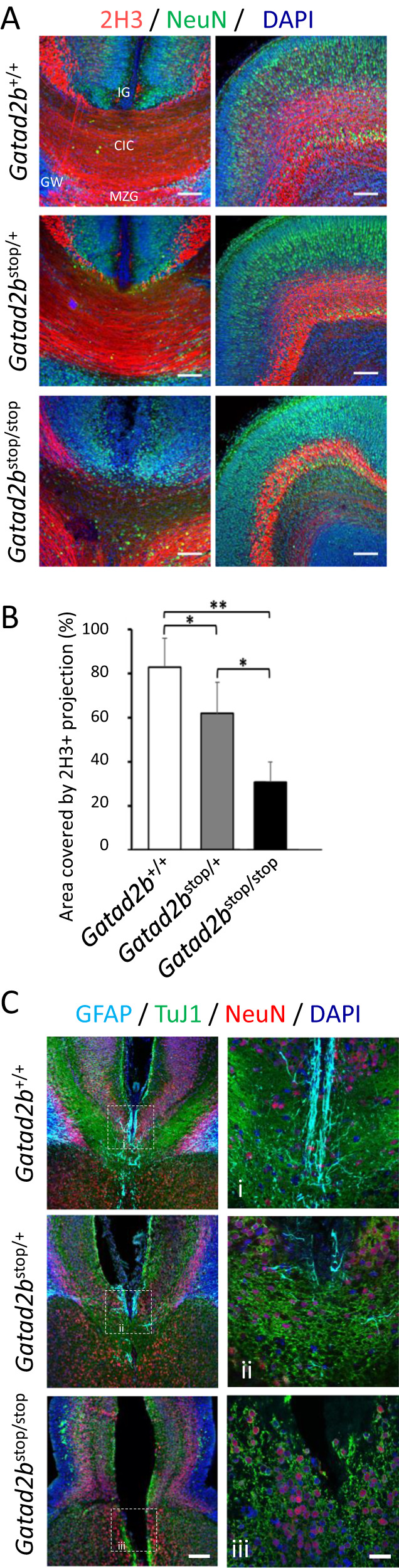
Disorganization of midline structures in *Gatad2b*^*stop/+*^ mice. **A** Immunostaining of brain coronal sections just rostral to the fornix and the anterior commissure (which were not overtly affected in the *Gatad2b*^*stop/+*^ brains by bright field microscopy), from P0 mice from the indicated genotypes for 2H3 (red, stains the pioneer axons of the cingulate cortex (CIC)) and NeuN (green). DAPI (blue) was used to identify nuclei. Most *Gatad2b*^*stop/stop*^ axons fail to reach the contralateral hemisphere, while significantly less *Gatad2b*^*stop/+*^ axons cross the midline. Scale bar: 100 µm. **B** Area covered by 2H3 projections, quantified for each genotype (*n* = 3 *Gatad2b*^*+/+*^*, n* = *2 Gatad2b*^*stop/+*^, *n* = 2 *Gatad2b*^*stop/stop*^, all from the same litter). *P < 0.05 (Student’s t-test). Data are presented as the mean ± S.E.M. **C** Immunostaining for glial cells with antibodies for GFAP (teal) in E18 embryos shows less glial cells and disorganization of the indisium griseum (IG), glial wedge (GW) and midline zipper glia (MZG). Labeling of neuronal cytoskeleton with TuJ1 (green) and NeuN (red) confirms the reduction of midline crossing axons. i-iii are enhanced magnifications of the indicated areas. Scale bars: 90 µm (left panels) and 30 µm (right panels). Images are from single embryos of either sex (siblings) but representative of an additional set of single embryos of each genotype derived from another breeding pair.

Cortical lamination is instrumental for proper development of callosal projections and several GAND subjects exhibited corpus callosum hypoplasia [5]. This led us to examine the brain midline of *Gatad2b*^stop*+/−*^ mice. We identified significant alterations, including reduced number of callosal projections and decreased number of midline astroglia cells (Fig. 5A–C). Midline glia populations originate from radial glia cells and control the correct navigation and midline crossing of callosal axons. These glial-cell types include the glial wedge (GW), indusium griseum glia (IGG), and midline zipper glia (MZG) subtypes and we observed a generalized reduction of all GFAP glia in *Gatad2b*^stop/+^ mice, with a virtually complete absence of GFAP expressing glia in *Gatad2b*^stop*/stop*^ E18 brains. Thus, reduced midline crossing of commissural axons in *Gatad2b* deficient mice is associated with an aberrant arrangement of GFAP-positive guidepost glia cells and a widening of the interhemispheric fissure (Fig. 5A–C). These alterations were observed in sibling mice derived from 2 different breeding pairs, suggesting that this phenotype is not the result of a stochastic defect but from deficiency of Gatad2b.

### Single-cell RNA-sequencing reveals abnormal cellular proportions in the cortex of *Gatad2b*^stop/+^ mice

We the performed single-cell RNA sequencing (scRNA-seq) on cortical samples obtained from *Gatad2b*^stop/+^ and WT littermates at a single embryonic time point (E16.5) in order to explore underlying mechanisms that contribute to the abnormal cortical development observed in the Gatad2b haploinsufficient mice. We also generated *Gatad2b*^stop/stop^ scRNA-seq data to identify dosage-sensitive targets of *Gatad2b* regulation. Altogether, 62,921 cells were sequenced in these studies.

**Fig. 6:**
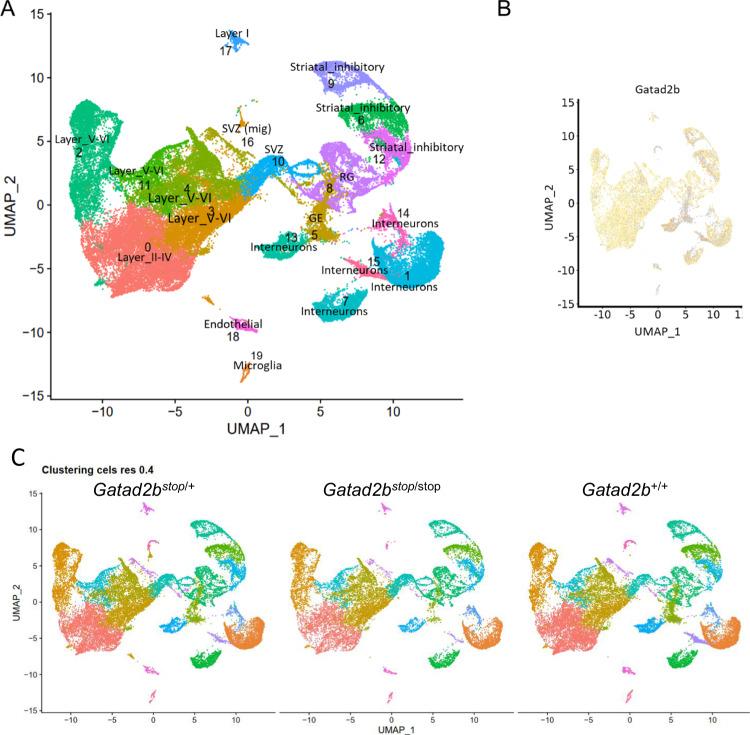
Cell clusters and cell identities of *Gatad2b* mutant mice. **A** Uniform manifold approximation and projection (UMAP) plot of single cells from 3 *Gatad2b*^+/+^, 4 *Gatad2b*^stop/+^ and 3 *Gatad2b*^stop/stop^ embryos, with each cell colored according to cluster. **B** UMAP of the integrated scRNA-seq data colored for the visualization of Gatad2b expression. **C** UMAP visualization of scRNA-seq data from individual genotypes.

We integrated expression data from WT and *Gatad2b* mutant cells to perform cell clustering using Seurat (Stuart et al. 2019). We categorized the clusters into 20 distinct cell populations (Table 1, Fig. 6A) based on expression of canonical markers [28–31]. Cell clusters included two major populations of excitatory cortical neurons: one cluster of upper-layer neurons (layer II through IV) and 4 clusters of deeper-layer neurons (layers V and VI). Inhibitory subpopulations included one cluster of layer I neurons, 5 interneuron cortical clusters and 3 striatal clusters. Proliferative cells included two clusters of subventricular zone progenitors, one of ganglionic eminence and one cluster of radial glia. Additionally, there was one cluster of microglia and one of endothelial cells. *Gatad2b* was expressed across all cell populations (Fig. 6B).

**Table 1:** Variability in cell type proportions detected by scRNA-seq in *Gatad2b* mutant brains.

Cluster	Cell type	% of total	WT (avg%)	Het (avg%)	KO (avg%)	*p* value (Het vs WT)	*p* value (KO vs WT)
0	Layer II-IV	16.22	19.27	17.25	14.92	0.149	0.013
1	IN	10.62	9.83	11.13	9.93	0.183	0.936
2	Layer V-VI	10.94	8.93	10.81	11.75	0.165	0.280
3	Layer V-VI	11.67	8.54	11.35	11.81	0.133	0.006
4	Layer V-VI	9.32	8.22	8.91	9.58	0.223	0.058
5	GE	3.14	7.44	3.65	3.12	**0.020**	**0.004**
6	Str. Inh	4.05	4.84	4.11	4.08	0.201	0.224
7	IN	4.19	4.08	4.22	4.11	0.782	0.914
8	RG	6.56	4.34	5.72	6.66	**0.024**	**0.001**
9	Str. Inh	3.76	3.80	3.83	3.78	0.923	0.799
10	SVZ	5.07	3.23	4.49	4.84	**0.048**	**0.000**
11	Layer V-VI	3.72	3.01	3.69	4.05	0.040	0.243
12	Str. Inh	3.71	2.99	3.58	3.73	0.306	0.269
13	IN	2.32	4.81	2.39	2.09	**0.011**	**0.011**
14	IN	1.27	1.22	1.02	1.30	0.737	0.595
15	IN	0.56	2.00	0.68	0.60	**0.006**	**0.006**
16	SVZ mig	0.94	1.09	1.10	0.87	0.979	0.162
17	Layer I	0.85	0.85	0.96	0.88	0.422	0.125
18	Endo	0.62	0.92	0.63	0.55	0.072	0.158
19	micro	0.455	0.580	0.483	0.430	0.194	0.028

Relative proportions of cortical layers II-IV (Layer II-IV), interneurons (IN), cortical layers V-VI (Layer V-VI), ganglionic eminence (GE), striatal inhibitory (Str. Inh), radial glia (RG), subventricular zone (SVZ), migratory SVZ (SVZ mig), cortical layer I (Layer I), endothelial (endo) and microglia (micro) are presented across all genotypes (% of total), and per genotype (*Gatad2b*^+/+^: WT (avg%), *Gatad2b*^stop/+^: Het (avg%), *Gatad2b*^stop/stop^: KO (avg%)). Bold values (in last two columns) highlight significant *p* values (*p* < 0.05) in both comparisons, *Gatad2b*^+/+^ vs. *Gatad2b*^stop/+^: *p* value (Het vs WT) and *Gatad2b*^+/+^ vs. *Gatad2b*^stop/stop^: *p* value (KO vs WT).

**Fig. 7:**
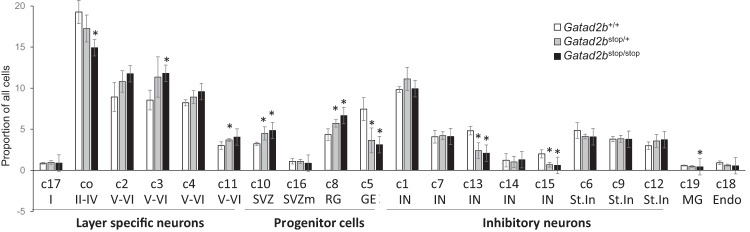
Alterations of cell type proportions in cortex of *Gatad2b* mutant mice. Plot of proportion of cells assigned to each cell type in *Gatad2b*^+/+^ (WT), *Gatad2b*^stop/stop^ (het) and *Gatad2b*^stop/stop^ (ko) E16.5 developing cortex. cortical layers II-IV (Layet II-IV), interneurons (IN), cortical layers II-IV (Layer V-VI), ganglionic eminence (GE), striatal inhibitory (Str. Inh), radial glia (RG), subventricular zone (SVZ), migratory SVZ (SVZ mig), cortical layer I (Layer I), endothelial (endo) and microglia (micro) are presented as across all genotypes (% of total), and per genotype (*Gatad2b*^+/+^: WT (avg%), *Gatad2b*^stop/+^: Het (avg%), *Gatad2b*^stop/stop^: KO (avg%)). Bolded values (last two columns) highlight significant *p* values (*p* < 0.05) in both comparisons, *Gatad2b*^+/+^ vs. *Gatad2b*^stop/+^: *p* value (Het vs. WT), and *Gatad2b*^+/+^ vs. *Gatad2b*^stop/stop^: *p* value (KO vs. WT).

Analysis of the proportion of cells of the clusters in the different genotypes revealed differentially relative abundance of subpopulations in *Gatad2b*^stop/+^ and WT embryos (Fig. 7). Interestingly, there was a significant increase in the proportion of progenitor-like cells in the subventricular zone (cluster 10) and radial glial cells (cluster 8) in *Gatad2b*^stop/+^ as compared to WT embryos, which was further increased in *Gatad2b*^stop/stop^ neocortices. In contrast to this finding, the proportion of ganglionic eminence cells is reduced in *Gatad2b*^stop/+^ and *Gatad2b*^stop/stop^ brains (Table 1). These observations suggest an excitatory-inhibitory imbalance derived from abnormal neurodevelopment. Consistently, the data showed decreased proportions of interneurons in the E16 cortex of *Gatad2b*^stop/+^ (Table 1, Fig. 7).

The scRNAseq data was then analyzed jointly (pseudo-bulk approach) across all cell types and identified 44 differentially expressed genes (DEG, adjusted *p* value < 0.05, average log 2-fold change > ABS 0.25) in *Gatad2b*^stop/+^. 60.0% (26/44) of these genes were downregulated (Supplementary Table 3). All the 44 genes with overall dysregulation in *Gatad2b*^stop/+^ embryos were also identified as DEG in the WT vs *Gatad2b*^stop/stop^ comparison, with larger expression differences in *Gatad2b*^stop/stop^ than in *Gatad2b*^stop/+^ samples (Table 2, Supplementary Table 4), including the NuRD subunit *Cdk2ap1*, which was upregulated in both *Gatad2b*^stop/+^ and *Gatad2b*^stop/stop^ E16 brain cortices in the joint analysis. Pathway enrichment analysis of these pseudo-bulk DEGs (excluding Gatad2b from the list to reduce bias) revealed significant enrichment of genes matching the membership term: neuron development (*p* = 0.0015, Supplementary Fig. 3).

**Table 2:** Genes with differential expression in the *Gatad2b*^stop/+^ vs. *Gatad2b*^+/+^ pseudo-bulked comparison (Het vs. WT).

Gene	FC Het vs WT	FC KO vs WT	FC Ratio
Hist1h1e	0.687	1.040	1.513
Frmd4a	−0.311	−0.484	1.559
Cbx3	0.328	0.515	1.570
Ntrk2	−0.255	−0.403	1.579
Slc24a5	0.317	0.511	1.612
Gm10076	−0.467	−0.756	1.618
Rps29	−0.356	−0.583	1.639
Hist1h1b	0.375	0.618	1.647
Nrxn1	−0.287	−0.478	1.666
Uba52	−0.436	−0.731	1.676
Rps28	−0.353	−0.597	1.688
Atp5k	−0.277	−0.469	1.690
Itpr1	−0.263	−0.449	1.707
Gsdme	−0.277	−0.476	1.719
Hmgb2	0.336	0.580	1.727
H3f3b	0.375	0.654	1.745
Arpp21	−0.372	−0.654	1.759
Jund	0.320	0.563	1.761
Lsamp	−0.280	−0.512	1.828
Rpl38	−0.379	−0.692	1.829
Mef2c	−0.408	−0.769	1.884
Mdk	0.358	0.676	1.887
Ptn	−0.317	−0.602	1.897
Ptprd	−0.300	−0.575	1.914
Cdk2ap1	0.287	0.563	1.959
Mapt	−0.315	−0.625	1.982
Nfia	0.409	0.829	2.029
H1f0	0.264	0.536	2.029
Gatad2b	−0.305	−0.621	2.038
Grin2b	−0.343	−0.714	2.085

The fold change (FC) of the *Gatad2b*^stop/stop^ vs. *Gatad2b*^+/+^ analysis (KO vs. WT) is also shown. Although all Het vs. WT DEG were also differentially expressed in the KO vs. WT, genes presented here have a FC KO vs. WT/FC Het vs. WT > than 1.5, suggesting a direct relationship of Gatad2b deficiency with their expression level.

### Cell-type-specific transcriptome analysis identifies *Gatad2b* dosage-sensitive genes

We then compared gene expression of *Gatad2b*^stop/+^ vs. WT cells for each cluster to identify cell-type-specific DEGs in the *Gatad2b*^stop/+^ brain (Supplementary Fig. 4). We detected 251 DEGs (adjusted *p* value < 0.05 and log 2 absolute fold-change > 0.25) across all individual clusters, with 60% of the identified DEGs being downregulated in the *Gatad2b*^stop/+^ cortices. Due to overlap in the DEGs between clusters, the 251 DEG includes 172 unique genes (Supplementary Table 5), with 43 genes differentially expressed in multiple clusters (Supplementary Table 6). Of note, *Gatad2b* haploinsufficiency caused significant alterations in gene expression in every cluster containing more than 500 cells (Supplementary Table 5).

We then leveraged information from the *Gatad2b*^stop/stop^ brains to identify genes likely to be direct targets of Gatad2b regulation by selecting those that were differentially expressed in *Gatad2b*^stop/+^ vs. WT, that also showed significant differential expression in *Gatad2b*^stop/stop^ vs. WT and in *Gatad2b*^stop/+^ vs. *Gatad2b*^stop/stop^ comparisons. In addition, the effect size should be larger in *Gatad2b*^stop/stop^ vs. WT than in the other two comparisons. This method identified 49 Gatad2b dosage-sensitive genes (Supplementary Table 7), which were significantly enriched in “neuron development” (*p* = 1.1E-12, Supplementary Fig. 5). Interestingly, the scRNAseq data showed increased transcriptional expression of *Bcl11b* in layer V-VI neurons (clusters 3 and 11) in *Gatad2b*^stop/+^ cortex. This data, along with our observation of increased colocalization of Ctip2 (encoded by *Bcl11b*) and Satb2 immunostaining signal in *Gatad2b*^stop/+^ brain sections (Fig. 4), led us to evaluate the percentage of cells that co-expressed *Bcl11b* and *Satb2* across the different clusters. Consistent with the immunostaining data, we observed an elevated proportion of single cells co-expressing *Ctip2* and *Satb2* in layer V-VI (clusters 2 and 3) and layer II-IV (cluster 0) neurons. This increase was largest in the *Gatad2b*^stop/stop^ cortices, where we observed significant increase in expression of *Bcl11b* in neurons (clusters 0, 2–4 and 11), interneurons (clusters 1 and 7) and in subventricular progenitor cells (cluster 10) (Supplementary Table 8). Expression of Satb2 was not significantly different in these cells, suggesting that the enhanced expression of *Bcl11b* is not due to reduced transcription of its repressor Satb2. Conversely, *Lmo4*, suggested to promote Bcl11b expression by interfering with Satb2-mediated repression, was upregulated in cortical layer neurons (Cluster 0 and cluster 2) of *Gatad2b*^stop/+^ mice (Supplementary Table 8).

## Discussion

The majority of *GATAD2B* variants associated with GAND are predicted to be loss of function, which suggests haploinsufficiency is the pathogenic mechanism of GAND. *GATAD2B* missense variants have also been identified in GAND subjects and localize within domains (CR1 or CR2) that are critical for protein-protein interactions between GATAD2B and other NuRD subunits (e.g. MBDs or CHDs), [5], which further suggests a functional haploinsufficiency effect [9, 34]. Although there is a small increased frequency of epilepsy in missense carrying subjects, GAND subjects possessing LoF and missense *GATAD2B* variants typically present with similar phenotypes, indicating that haploinsufficiency models should be appropriate to model GAND [5]. Our data shows that *Gatad2b* heterozygous mice, which express approximately 50% of *Gatad2b*, exhibit a cognitive phenotype substantiate the haploinsufficiency model of human GAND. How reduced *Gatad2b* expression results in the clinical presentation of GAND is unknown. However, our observation of defective cortical development in *Gatad2b* haploinsufficient mice suggests abnormal neurodevelopment as the underlying cause of GAND.

The cortex is a highly complex brain structure that is organized into six layers, with each layer containing highly specialized cell types that receive and extend different projections to and from specific targets. The complex organization of the cortex reflects the precise orchestration of cell replication, migration and differentiation during embryogenesis. The immunohistochemical data indicating cortical alterations in *Gatad2b* mutant brains, including increased Satb2-Ctip2 co-expression, suggested that *Gatad2b* is involved in the spatiotemporal regulation of gene expression in the developing cortex necessary for the organization of a wide variety of neuronal and non-neuronal cell types into a functional unit that supports cognitive processes. The scRNAseq data from Gatad2b haploinsufficient embryonic cortex revealed misexpression of key developmental genes. One example of this is the reduced expression of Pou3f2/Brn2, a transcriptional regulator that controls neural precursor proliferation [35–37], in subventricular progenitor cells from *Gatad2b*^stop/+^ cortices (Supplementary Table 5). Interestingly, the scRNAseq data also showed elevated content of Mki67 in the ganglionic eminence cell cluster in these Gatad2b mutant brains. Altogether, these data suggest cell type-specific abnormal cell proliferation associated to Gatad2 deficiency, likely decreasing proliferation in the SVZ and enhancing replication in the ganglionic eminence probably affecting cellular content in the cortex.

An additional example of altered gene expression in Gatad2b-deficient mice with potential to alter cortical development includes the significant increase of *Bcl11b* (Ctip2) expression in deep-layer neurons of Gatad2b heterozygous brains. Overexpression of *Bcl11b*, which is more pronounced in *Gatad2b*^stop/stop^ brains (Log2 FC in *Gatad2b*^stop/stop^ vs. WT is 0.8 while in *Gatad2b*^stop/+^ vs. WT is 0.35) likely reflects the incomplete repression of *Bcl11b* expression by NuRD and Satb2. The observed increase in *Bcl11b* may alter neuronal connections (e.g. transcallosal cortico-cortical projections) [38–40].

To better define the Gatad2b-regulated transcriptional programs involved in corticogenesis, we focused on genes with Gatad2b dose-dependent expression. Consistent with recent data suggesting repressive as well as activating activities of NuRD, genes identified as Gatad2b dosage-sensitive are upregulated and downregulated in Gatad2b mutant brains. Furthermore, some of these Gatad2b dose-sensitive genes were shared between different cell types, while others had more cell-type specificity. Gatad2b dosage-sensitive genes differentially expressed in multiple cell types include *H3f3b*, which has been previously shown to mediate neuronal fate specification through epigenetic transcriptional regulation [41] and to be associated with neuro-developmental delay, dysmorphic features, and brain abnormalities [42] and *Nfia*, which has been reported to control gliogenesis [43] and associated with brain malformations [44]. In addition, *Lmo4* and *Sox5*, which have been shown to participate in neuron differentiation [45–48] with SOX5 being clinically associated with an NDD with apraxia of speech [49]. *Arpp21* and the autism gene *Grin2b*, that control dendritogenesis and branching [50, 51] were also Gatad2b dosage-sensitive genes differentially expressed in more than one cell cluster, with Grin2b being clinically associated with epilepsy and autism [52]. Cell cluster-specific Gatad2b dosage-sensitive genes include *Frmd4a*, associated with intellectual disability [53, 54] and *Robo1* that mediates proliferation and neurogenesis in development and is clinically associated with a neurodevelopmental disorder [55, 56] in cluster 0 (Layer II-VI). In cluster 1 interneurons, *Cdkn1c*, involved in cell survival during cortex development and Beckwith-Wideman syndrome [57, 58] along with the dendritic organizer *Chl1* [59] were identified as dosage-sensitive genes. Layer V-VI cluster-specific dosage-sensitive genes include a regulator of neuron migration, *Dpy19l1* (cluster 2) [60] and *Bcl11b* (cluster 3), which is clinically associated with neurodevelopmental and immunological disorder [61]. Thus, we have uncovered genes involved in proliferation, migration and differentiation, many of which are involved in neurodevelopmental disorders, as being associated with Gatad2b expression. These data support a critical role for Gatad2b in the regulation of neural migration, neural proliferation, and the cell cycle during cortical development. Since these data are correlative, follow up experiments will determine whether the identified genes are direct targets of Gatad2b regulation, are part of a Gatad2b regulated program, or are dysregulated as a secondary response to altered corticogenesis.

Our data showing that homozygous mutation of Gatad2b is inconsistent with survival indicates that, similar to its paralog Gatad2a [62], Gatad2b plays an essential role in development. This is consistent with absence of identified homozygous carriers of *GATAD2B* loss of function mutations in humans. Interestingly, *Gatad2b*^stop*/stop*^ mice die perinatally, while Gatad2a KO mice die early during embryonic development. The distinct outcomes of homozygous mutation of the two paralogs could reflect functional specialization or, alternatively, similar functionality but differences in the timing/pattern of expression. The identification of Gatad2b and Gatad2a as mutually exclusive subunits in NuRD complexes [10, 63], together with the observation of a lack of compensatory activity in the observed lethality of Gatad2a^−/−^ and Gatad2b^stop/stop^ of the reciprocal paralog [62], suggests functional specification of both paralogs and in turn of the different NuRD complexes (e.g. GATAD2A-NuRD or GATAD2B-NuRD). Our transcriptional data suggest that haplo-insufficiency for Gatad2b does not induce compensatory upregulation of Gatad2a expression, suggesting a lack of regulatory feedback mechanisms between the two genes in mice. Whether Gatad2b haploinsufficiency effects are the result of reduced levels of functional NuRD, abnormally composed NuRD or even yet unknown NuRD-independent functions of Gatad2b is not known. The significant overlap of phenotypes between CHD3-, CHD4-, and CHD5-related disorders and GAND suggests a shared pathogenesis mechanism involving NuRD function/chromatin deposition [5, 6, 64–66].

These studies provide important resources for the GAND research community: we validated a mouse model for GAND that will be instrumental for identification of pathogenesis mechanisms and for therapeutic studies; we highlight cortical development as an important pathogenetic component of the disease; we identified cell-type specific transcriptional dysregulation associated with Gatad2b haploinsufficiency and linked Gatad2b to proliferation, migration and differentiation of neural progenitor cells. Our findings underscore the complex nature of the pathogenesis of GAND, which involves alterations in multiple genes and cell types. We hypothesize that the effect of Gatad2b haploinsufficiency in cortex development is the result of this collective transcriptional change, which in turn drives pathogenesis and behavioral disturbances. Future cellular and epigenetic profiling would be critical to identify cell autonomous vs non-autonomous effects, and analysis of other developmental stages will be important to define the Gatad2b transcriptional program.

### Supplementary information

Supplementary figures

Dataset 1

supplementary tables

## Data Availability

Single-cell RNA-sequencing data are available at the NCBI Gene Expression Omnibus (GEO) under accession number GSE244477.
