# Supplementary material for: Gatad2b, associated with the neurodevelopmental syndrome GAND, plays a critical role in neurodevelopment and cortical patterning
Source: Transl Psychiatry. 2024 Jan 18;14:33. doi: 10.1038/s41398-023-02678-x (PMC10796954; doi:10.1038/s41398-023-02678-x)

Supplementary figure 1.

Gatad2b targeted allele. Insertion of a *LacZ* trapping cassette and a floxed promoter-driven *Neo* cassette inserted into the intron of *Gatad2b* is expected to disrupt gene function.

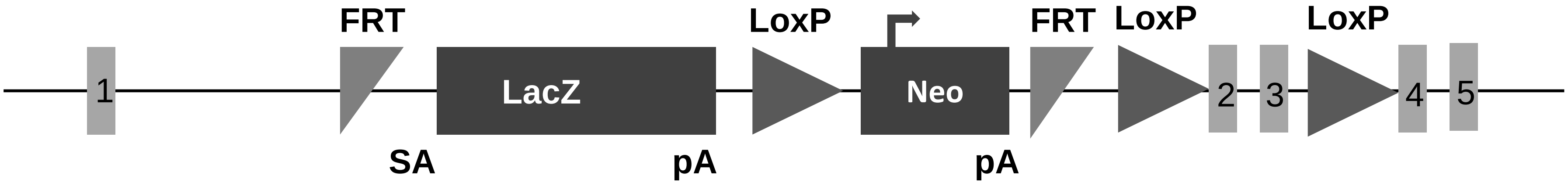

Supplementary Figure 2.

A. Measurement of nasal width. A digital caliper was used to measure the distance between the most lateral 3rd row of vibrissae in anesthetized *Gatad2b*$^{stop/+}$ (n=8) and *Gatad2b*$^{stop/+}$ (n=7) mice (*p=0.0002, unpaired Student's t-test). No significant difference was measured in head width.

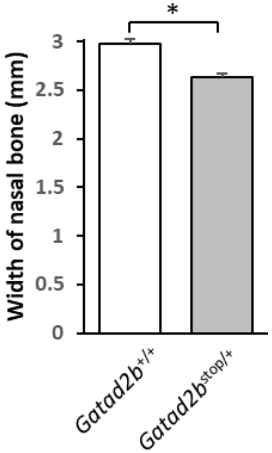

B. Facial dysmorphology in Gatad2b$^{stop/+}$ mice. The nasion (arrow), was elevated in the majority of the heterozygous mice.

Gatad2b$^{+/+}$

Gatad2b$^{stop/+}$

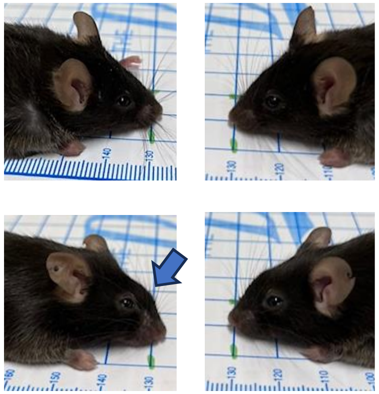

	Curved
+/+	14%
Stop/+	85%
chi-square *p*-value: 0 .0187	

Supplementary figure 3.

Enrichment of genes matching membership term: **neuron development** . The outer pie shows the number and the percentage of genes in the background that are associated with the membership (in black); the inner pie shows the number and the percentage of genes in the individual input gene list that are associated with the membership. The p-value indicates whether the membership is statistically significantly enriched in the list.

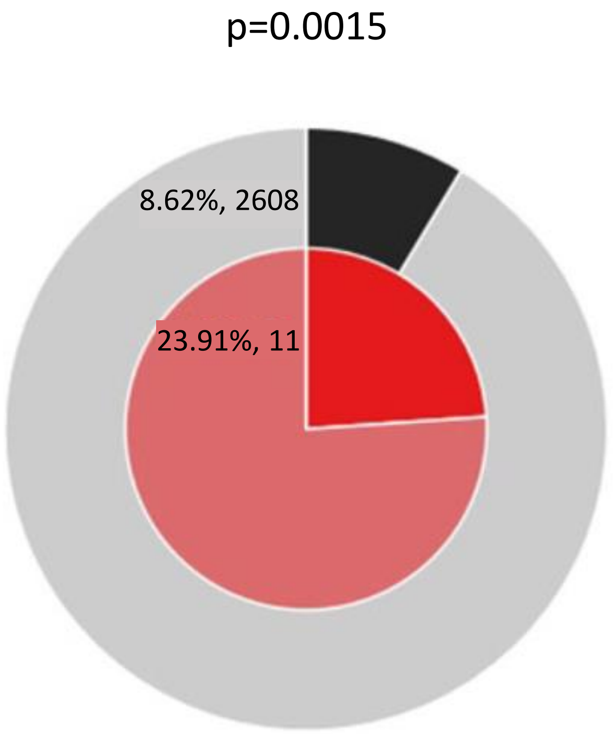

Supplementary Figure 4.

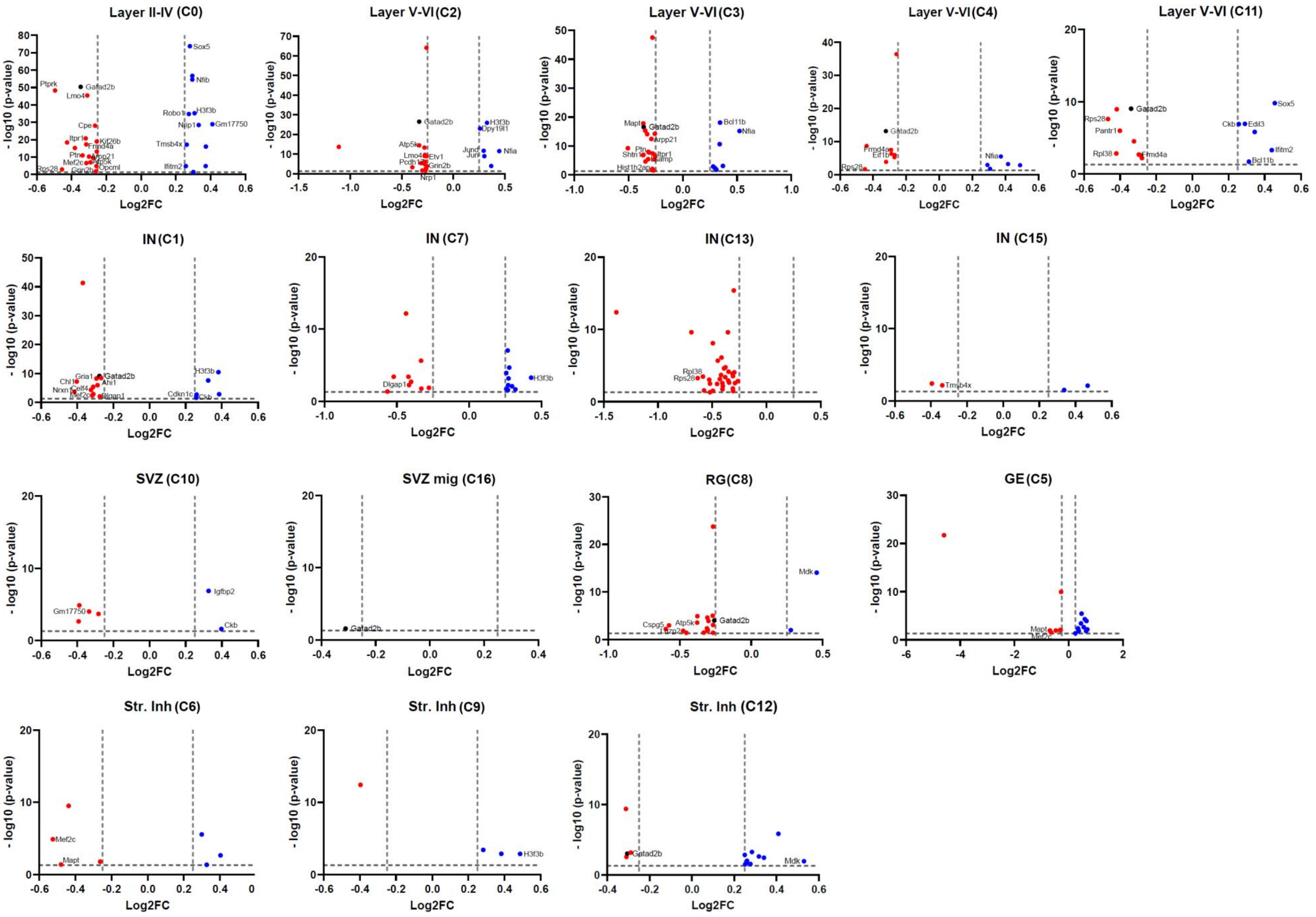

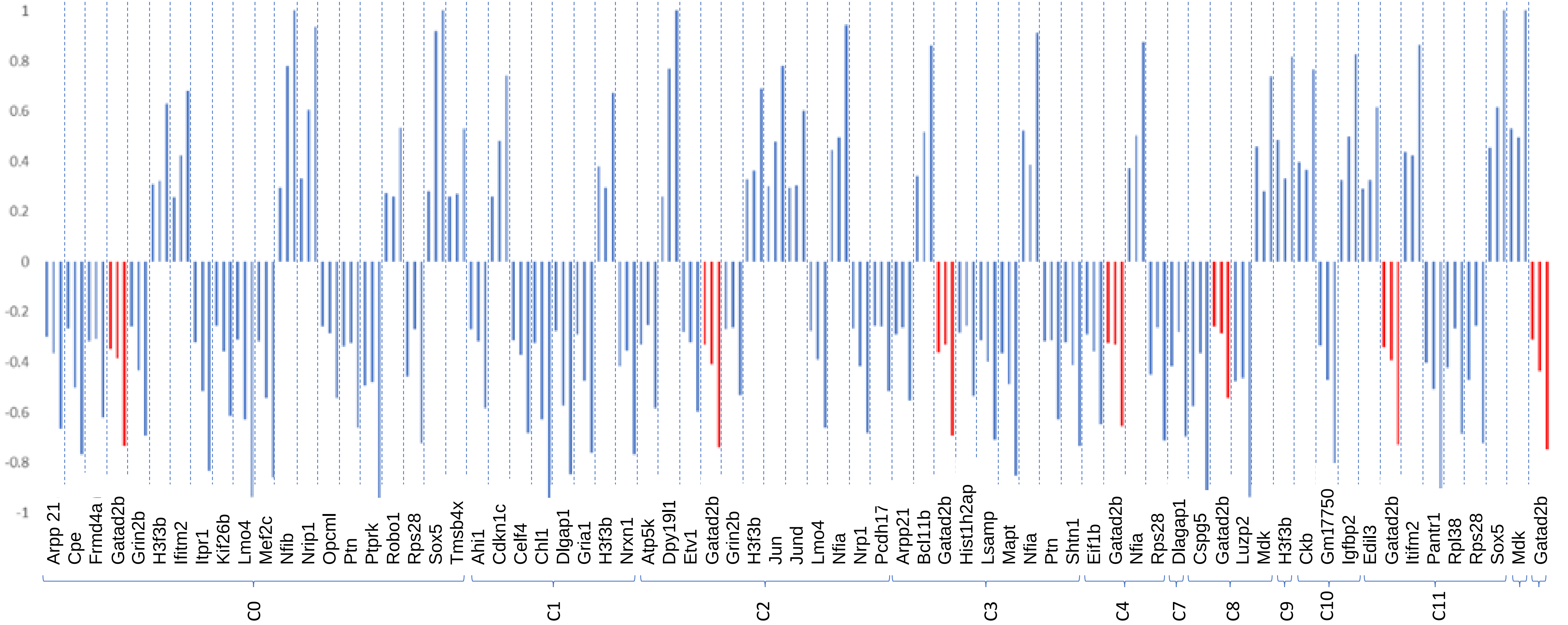

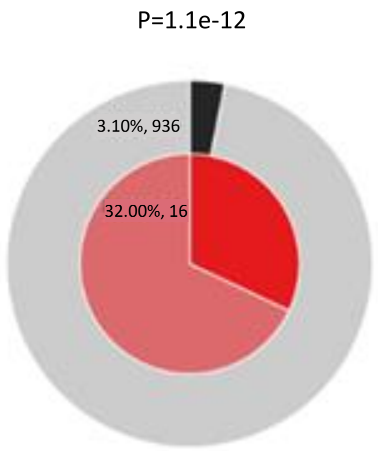

Enrichment of genes matching membership term: neuron development (GO:0048666). The outer pie shows the number and the percentage of genes in the background that are associated with the membership (in black); the inner pie shows the number and the percentage of genes in the individual input gene list that are associated with the membership. The p-value indicates whether the membership is statistically significantly enriched in the list.

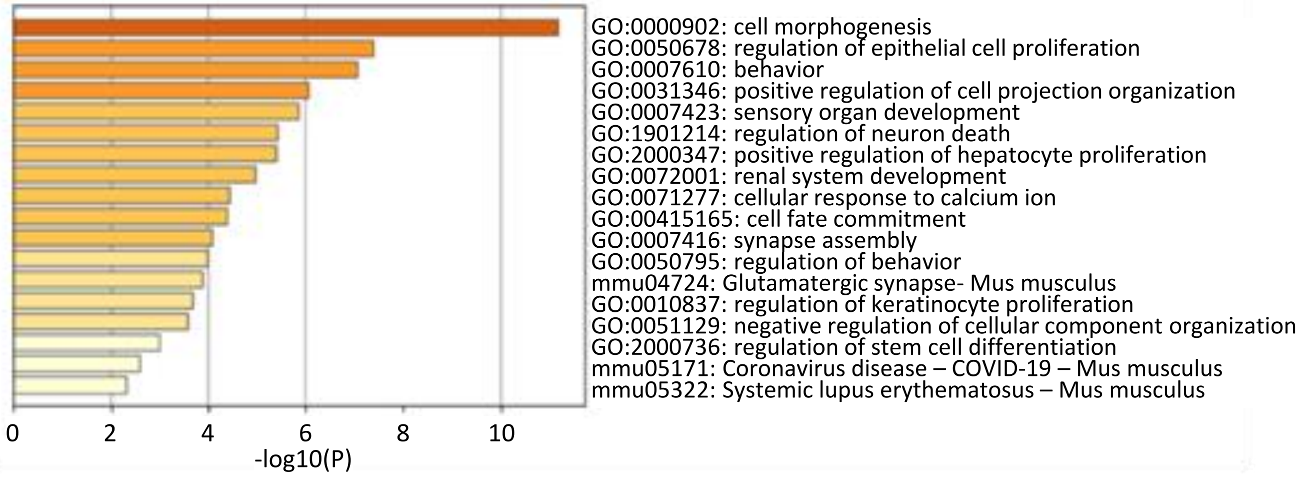

Supplement: Supplementary file 1 — Supplementary figures [file 41398_2023_2678_MOESM1_ESM.pdf]
