# Supplementary material for: Gatad2b, associated with the neurodevelopmental syndrome GAND, plays a critical role in neurodevelopment and cortical patterning
Source: Transl Psychiatry. 2024 Jan 18;14:33. doi: 10.1038/s41398-023-02678-x (PMC10796954; doi:10.1038/s41398-023-02678-x)
Supplement: Supplementary file 3 — supplementary tables [file 41398_2023_2678_MOESM3_ESM.pdf]

id		gene-name	chromosome	r	donor_id	DFC 8 pcw 13058	DFC 9 pcw 12833	DFC 12 pcw 12835	DFC 12 pcw 12960	DFC 12 pcw 13060	DFC 13 pcw 12820	DFC 13 pcw 12834	DFC 13 pcw 12888	DFC 16 pcw 12287	DFC 16 pcw 12837
1095367	GATAD2B	GATA zinc finger domain containing 2B	1	1		2.5266	4.6865	4.834	4.3716	4.6682	4.8599	4.7082	4.6635	4.2152	5.1297
1086962	MLL5		7	0.9		2.7252	4.8354	4.8352	4.3738	4.4353	4.6397	4.5694	4.1254	3.8812	4.7576
1104672	NCOA6	nuclear receptor coactivator 6	20	0.9		1.9691	4.6839	4.8235	3.9921	4.1958	4.5584	5.0161	4.3552	3.9786	5.0983
1089098	TCF20	transcription factor 20 (AR1)	22	0.9		2.2456	4.3663	4.7177	4.1267	4.023	4.4818	4.5452	4.0202	3.7995	4.4931
279991298		AC134943.1	3	0.9		2.7018	4.0066	4.4347	4.1282	3.5626	4.2057	4.2388	3.4385	3.4171	4.6001
1101925	SETD2	SET domain containing 2	3	0.9		2.1872	4.0514	4.0715	3.1835	3.6768	4.0031	4.0523	3.5187	3.3569	4.0385
1088486	ZNF8	zinc finger protein 8	19	0.9		1.0903	3.3908	3.1377	2.019	2.2774	2.873	3.166	2.1646	2.895	3.347
1097876	RPRD2		1	0.9		2.4255	3.8872	3.8152	3.5187	3.5858	3.8578	3.6896	3.445	3.2106	3.8123
1097192	C11orf30	chromosome 11 open reading frame 30	11	0.9		1.5032	2.8029	2.778	2.2081	2.4725	2.4543	2.6274	1.9333	2.0004	2.675
1102783	ZNF445	zinc finger protein 445	3	0.9		2.4473	3.76	4.1803	3.9189	3.4409	3.9869	3.8695	3.3373	3.3391	4.4272
1096103	CELF1	CUGBP, Elav-like family member 1	11	0.9		3.4708	4.591	5.1148	4.5362	4.703	4.8027	4.5665	4.0449	4.2535	5.0792
1095327	POGZ		1	0.9		2.4501	4.7048	4.5345	3.9217	4.0735	4.3954	4.5512	3.699	3.9478	4.4127
1092242	MED13L	mediator complex subunit 13-like	12	0.9		2.1926	3.9433	5.3818	4.8461	4.6174	4.7272	4.9172	3.3573	3.3061	4.8165
1096438	SETBP1	SET binding protein 1	18	0.9		2.2658	5.117	4.4826	3.892	3.8921	4.4412	4.1804	3.991	3.7093	4.5445
1088125	CLASP1	cytoplasmic linker associated protein 1	2	0.9		3.5109	4.51	5.1707	4.9294	4.8514	4.9406	5.0159	4.657	4.3488	5.4402
1104143	ZNF398	zinc finger protein 398	7	0.9		2.2916	2.8049	3.2103	2.9627	3.028	3.3416	3.1544	2.6393	2.8347	3.1496
1087078	CELSR3		3	0.9		1.8003	3.3719	4.469	3.6837	3.2518	4.255	4.2841	2.896	3.7927	4.8051
1091905	KIAA1267	KIAA1267	17	0.9		2.8688	4.7303	4.4622	3.8375	3.9861	4.1164	4.606	3.9315	4.0669	4.4517
1098110	YEATS2	YEATS domain containing 2	3	0.9		2.4389	3.898	3.84	3.0973	3.3025	3.6016	3.8877	3.0692	3.5735	3.9926
1089281	CHD8		14	0.9		2.798	4.2817	4.5468	3.7354	4.1487	4.5129	4.3852	3.7169	3.7952	4.4058
1096954	KAT6B	K(lysine) acetyltransferase 6B	10	0.9		2.4825	4.3386	4.2687	3.9555	3.9024	4.2143	4.0682	3.4905	3.1806	3.9562
1103877	ZNF836	zinc finger protein 836	19	0.9		1.0267	1.9783	3.0789	1.8998	2.8318	2.506	2.6943	2.5167	2.5764	2.4237
1095808	ATXN7L1	ataxin 7-like 1	7	0.9		0.638	1.7876	2.3508	2.1538	2.2911	2.6374	2.4156	1.815	1.7055	2.581
1087537	ARID1B		6	0.9		1.5939	3.3012	3.7147	2.8605	3.1206	3.4486	3.6842	2.9178	2.9614	3.6225
1094888	IGF1R	insulin-like growth factor 1 receptor	15	0.9		2.4745	4.4722	4.5424	4.3083	3.8359	4.5105	4.4053	3.3307	3.6619	4.8895
1091694	KLF7	Kruppel-like factor 7 (ubiquitous)	2	0.9		2.7157	3.8485	5.1917	4.8877	4.5853	4.897	4.7581	4.258	3.7367	4.8573
1091430	BCL9	B-cell CLL/lymphoma 9	1	0.9		3.2938	4.7138	4.8916	4.1044	4.47	4.6821	4.8719	4.2625	4.2258	5.1279
1091653	ARID1A	AT rich interactive domain 1A (SWI-like)	1	0.9		2.7031	5.4663	5.0482	4.1558	4.2886	4.8052	4.9272	4.3911	4.2495	5.2377
1088521	BCORL1	BCL6 corepressor-like 1	X	0.9		1.4899	3.4203	3.3021	2.7098	2.9106	3.2717	3.3729	3.0087	2.8559	3.6138
1093233	PRRC2B	proline-rich coiled-coil 2B	9	0.9		4.0233	6.3571	6.6628	6.0629	5.7891	6.6488	6.5138	5.4109	6.0475	7.0634
1086954	CREBBP	CREB binding protein	16	0.9		1.9906	4.8543	4.634	3.6943	3.8671	4.5529	4.5573	3.6291	4.0869	4.9996
1091820	YLPM1	YLP motif containing 1	14	0.9		2.6033	4.6335	4.5111	3.6659	4.3549	4.8796	4.3617	4.4382	3.8889	4.7936
1094240	UGGT1		2	0.9		2.1012	3.3529	3.528	2.8409	3.0964	3.5124	3.202	3.1208	2.707	3.437
1103456	ZNF490	zinc finger protein 490	19	0.9		1.055	1.7712	2.6394	2.0393	2.0822	2.3375	2.4979	1.4367	2.1032	2.7391
1087835	KDM4A	lysine (K)-specific demethylase 4A	1	0.9		4.0143	4.704	4.9876	4.6816	4.7695	4.9393	4.9748	4.6316	4.6919	4.9104
1104547	QRICH1	glutamine-rich 1	3	0.9		3.3821	4.3749	4.0417	3.7714	4.1124	4.0773	4.2695	3.8193	4.0562	4.2803
1098169	ZNF589	zinc finger protein 589	3	0.9		2.3946	3.3942	2.9852	2.8991	2.5834	2.8229	3.0704	2.8267	3.0063	2.863
279850307		RP11-175O19.4	10	0.9		1.9047	2.3091	3.2611	3.0261	3.0367	3.0993	3.1679	2.7982	2.3948	3.015
1104199	C20orf112		20	0.9		2.6488	4.4323	4.9112	4.2449	4.4671	4.6675	5.0108	4.559	4.2972	5.4593
279862629		RP11-261C10.4	1	0.9		1.5861	4.1194	5.0994	4.456	4.2932	4.5856	5.1897	3.6657	3.3512	4.6508
1094965	ZCCHC14	zinc finger, CCHC domain containing 14	16	0.9		2.6484	3.8935	4.5248	4.0608	3.8809	4.6025	4.2402	3.3906	4.0883	4.9192
1095293	ABL2		1	0.9		1.8272	2.6715	2.9983	2.8474	2.6268	2.8472	2.6143	2.2367	2.349	3.0995
1093256	ZNF317	zinc finger protein 317	19	0.9		3.3109	3.8462	4.0129	3.8963	3.8185	3.8715	3.9871	3.5794	3.6946	3.7669
1095381	CEP170	centrosomal protein 170kDa	1	0.9		3.0806	4.6193	5.4643	5.3931	5.5101	5.2805	5.3368	5.2631	4.2027	5.0748
1088692	KDM2B	lysine (K)-specific demethylase 2B	12	0.9		2.3944	2.9373	3.1367	2.9915	2.9913	3.3096	3.2186	2.5547	3.1744	3.3346
1101724	ZNF609	zinc finger protein 609	15	0.9		2.8125	5.4241	4.6033	4.0232	3.7881	4.2568	4.5655	3.8478	3.8139	4.9567
1092596	MED1	mediator complex subunit 1	17	0.9		2.7671	3.778	3.8163	3.6045	3.5467	3.6005	3.5526	3.2284	3.0298	3.6479
1101379	ZNF543	zinc finger protein 543	19	0.9		1.3925	2.536	2.4828	2.1664	2.0229	2.1725	2.3102	1.4478	2.1251	2.6032
279808690		RP11-261C10.5	1	0.9		2.2549	3.9674	4.4428	4.3805	4.5562	4.2885	4.3813	4.2455	3.1654	4.1393
1095013	SSH2	slingshot homolog 2 (Drosophila)	17	0.9		1.7591	2.8368	3.555	3.3228	2.8734	3.1671	3.1787	2.5699	2.8039	3.1351
1097683	GMEB1		1	0.9		2.285	2.8132	2.8577	2.6491	3.0372	3.1259	2.9846	2.9071	2.7297	2.7531
1094794	SETD1B	SET domain containing 1B	12	0.9		1.052	3.1946	3.4556	2.6266	2.6469	3.6152	3.0725	2.2018	2.9584	4.0495
279968499		AC004893.11	7	0.9		1.1224	2.1824	2.2274	1.7602	1.797	2.2856	2.2582	1.6657	1.6783	2.2968
280010958	ZNF154	ZNF154	19	0.9		1.9237	3.4573	3.6344	3.5552	2.9418	2.8432	3.4419	3.1399	2.8488	3.328
1101646	ZNF154	zinc finger protein 154	19	0.9		1.9077	3.4355	3.61	3.5335	2.9213	2.8216	3.4191	3.1181	2.8285	3.3073
1102306	ZNF623	zinc finger protein 623	8	0.9		2.812	3.1912	3.9119	3.7687	3.4189	3.6466	3.6823	2.9542	3.1845	3.5438
1100030	ZNF562	zinc finger protein 562	19	0.9		2.3	3.152	3.3936	3.0397	2.8955	3.153	3.4305	2.8658	2.9183	3.2053
1095881	MFHAS1		8	0.9		2.5721	2.9809	3.9673	3.395	3.1851	3.704	3.6818	3.0292	2.9621	3.6888
1095141	DOPEY2	dopey family member 2	21	0.9		1.6552	3.2995	4.1079	4.0463	4.36	4.5781	4.3398	3.8546	3.5905	4.8065
1105800	ZNF611	zinc finger protein 611	19	0.9		1.4901	1.8306	2.508	2.3196	2.4684	2.508	2.3652	2.1228	2.1012	2.5314
1091070	DROSHA	drosha, ribonuclease type III	5	0.9		3.0383	4.0095	3.978	3.4978	3.9903	3.9898	4.0899	3.91	3.536	3.7993
1100027	ASXL1	additional sex combs like 1 (Drosophila)	20	0.9		2.9214	4.1511	3.8995	3.4762	3.4747	3.791	3.8411	2.9257	3.6312	4.0837
1095034	ASXL3	additional sex combs like 3 (Drosophila)	18	0.9		0.7482	2.7246	4.3277	3.4682	3.3374	3.9102	3.8793	2.8237	2.6284	4.5082
1099482	CSNK1G1	casein kinase 1, gamma 1	15	0.9		2.6204	2.6675	3.2496	3.1266	2.9123	2.7091	2.8355	2.5751	2.2426	2.6664
1090384	ZMIZ1	zinc finger, MIZ-type containing 1	10	0.9		3.2042	5.4628	5.4078	4.8942	4.8128	5.4704	5.4702	4.6703	5.1422	6.08
1090982	KIAA0240	KIAA0240	6	0.9		1.5756	2.6269	3.7644	3.5395	3.4209	3.4477	3.5836	2.9688	2.6421	3.4727
1104745	LRIG2		1	0.9		0.9784	2.5748	2.5036	2.2757	1.8667	1.9637	2.2066	1.9076	1.5954	2.2363
1098607	NSD1		5	0.9		2.6572	4.1875	3.8318	3.2975	3.0633	3.6148	3.5261	2.8196	3.2351	3.7523
1089345	DIDO1	death inducer-obliterator 1	20	0.9		2.3231	2.999	3.2019	2.7285	2.728	3.1524	2.9404	2.2945	2.8772	3.274
280021880	DNAH10OS		12	0.9		0.7183	3.2444	4.1194	2.9401	2.8194	3.9053	3.9637	2.8503	3.1713	4.6857
1091673	MLL		11	0.9		1.1649	4.3626	3.9988	2.5848	2.8059	3.4404	3.7072	2.778	2.9401	4.1985
1105039	TMEM57	transmembrane protein 57	1	0.9		3.8517	4.0505	4.9732	5.0189	5.3318	5.4054	4.903	5.1709	4.7656	4.8308
1088219	PLXNA2	plexin A2	1	0.9		2.6792	4.135	4.9025	4.6043	4.2545	5.876	5.1161	3.3766	4.9332	5.927
1087421	TRIO		5	0.9		1.8169	3.7986	4.9938	3.9855	4.0261	4.8441	4.7906	3.4929	4.2878	5.0443
1090182	USP42	ubiquitin specific peptidase 42	7	0.9		1.9865	2.6727	2.9146	2.7823	2.8176	2.995	2.6892	1.8922	2.4813	2.8966
1095582	VPRBP	Vpr (HIV-1) binding protein	3	0.9		3.2303	4.186	3.9063	3.4695	3.5766	3.6496	3.8324	3.5798	3.3872	3.6651
280039366		AC068134.10	2	0.9		1.271	2.785	2.649	2.0338	1.6069	2.8952	2.8329	1.9511	1.9116	2.8779
1094236	SAP130	Sin3A-associated protein, 130kDa	2	0.9		2.804	4.1322	4.1786	3.5431	3.9437	3.7855	4.3655	3.962	3.705	4.1869
280111205		RP11-493P1.2	12	0.9		1.7515	3.7911	5.1756	4.5698	4.2731	4.2564	4.9466	2.9155	3.0939	4.5481
1093666	MORC2	MORC family CW-type zinc finger 2	22	0.9		3.4875	4.3403	4.3259	3.8349	4.2342	4.5224	4.4143	4.1054	4.2352	4.4321
1087920	POLR1A		2	0.9		1.6763	3.3459	3.3574	2.5406	2.5132	3.2953	3.0315	2.4072	2.6395	3.334
1094259	RALGPS1		9	0.9		2.0561	2.8789	3.9273	4.0129	3.6106	4.1142	3.8454	3.2987	3.3219	4.377
1101369	ZNF454	zinc finger protein 454	5	0.9		1.479	1.8817	2.3688	1.5822	1.9029	2.2628	2.2336	1.8378	1.7641	2.0465
1093173	MLLT4		6	0.9		3.5215	5.3351	6.0481	5.2567	5.5554	5.5319	5.7848	4.7473	4.3438	4.9582
1089325	ADNP		20	0.9		4.4887	5.4224	5.6732	5.3327	5.3612	5.707	5.4353	4.8042	5.0301	5.3986
1096118	NCAM1	neural cell adhesion molecule 1	11	0.9		4.8241	6.3766	6.9774	6.9064	6.9513	7.2935	7.1269	6.9959	5.8383	6.9286
1102158	TTC3	tetratricopeptide repeat domain 3	21	0.9		4.111	4.8629	5.5289	5.7461	5.5629	5.3163	5.5431	5.5266	4.8953	5.5748
1095464	AFF3	AF4/FMR2 family, member 3	2	0.9		2.0476	4.075	5.8413	5.6772	5.4946	5.7265	5.8498	5.2361	4.0514	5.6048
1093790	KIDINS220	kinase D-interacting substrate, 220kDa	2	0.9		4.7723	5.7308	7.1925	7.0788	6.9728	7.0763	6.9137	6.7971	5.4469	6.6941
1096072	PDCD11	programmed cell death 11	10	0.9		3.086	3.5558	3.7097	3.0666	3.2404	3.5848	3.5126	2.8265	3.3289	3.5231
1100960	JAKMIP2	[illegible]	5	0.9		3.1228	3.8111	4.2942	4.1767	4.0693	4.1912	4.1179	3.7148	3.3638	4.1567
279914319		RPS-1184F4.5	20	0.9		1.348	3.3378	3.5994	2.8006	2.8504	3.2742	4.0027	2.7868	3.2211	4.2833

[illegible]

MFC 2 yrs	MFC 3 yrs	MFC 8 yrs	MFC 8 yrs	MFC 11 yrs	MFC 13 yrs	MFC 18 yrs	MFC 19 yrs	MFC 21 yrs	MFC 23 yrs	MFC 30 yrs	MFC 36 yrs	MFC 37 yrs
12979	12980	12841	12981	12289	12831	12984	12832	13057	12300	12290	12302	12303
2.1939	2.7601	2.6527	2.032	3.5412	3.6748	2.3082	3.5088	3.3006	3.3595	3.2365	3.5182	3.3775
2.2833	3.0573	2.4529	2.1543	3.3691	3.3161	2.8049	3.2537	3.2716	3.6303	2.9338	3.1666	3.5529
1.245	2.6006	1.5682	1.6219	3.0125	3.0476	2.0764	3.0801	2.6173	2.8888	2.8293	3.1324	3.2078
[illegible]												

OFC 8 pcw	OFC 9 pcw	OFC 12 pcw	OFC 12 pcw	OFC 12 pcw	OFC 13 pcw	OFC 13 pcw	OFC 13 pcw	OFC 16 pcw	OFC 16 pcw	OFC 17 pcw	OFC 21 pcw	OFC 24 pcw	OFC 37 pcw
13058	12833	12835	12960	13060	12820	12834	12888	12287	12837	12880	12886	12288	3E+08
2.3017	4.6595	4.6208	3.834	4.6377	4.2811	4.6203	3.3634	4.4877	5.0862	4.4373	4.4061	4.358	3.3026
2.7009	4.6306	4.5824	4.0247	4.6709	4.2335	4.6353	3.4841	4.0498	4.8846	4.3769	4.2167	4.1158	3.6313
1.6416	4.6091	4.3987	3.429	4.4017	4.0263	4.9581	2.4413	4.1742	5.1325	4.3972	4.2754	4.326	3.8578
[illegible]													

OFC 4 mos	OFC 4 mos	OFC 10 mos	OFC 1 yrs	OFC 2 yrs	OFC 3 yrs	OFC 8 yrs	OFC 11 yrs	OFC 13 yrs	OFC 18 yrs	OFC 19 yrs	OFC 21 yrs	OFC 23 yrs	OFC 30 yrs	OFC 36 yrs	OFC 37 yrs	OFC 40 yrs
12889	12890	12977	12830	12979	12980	12841	12289	12831	12984	12832	13057	12300	12290	12302	12303	12304
3.2482	2.2967	3.4534	3.5411	2.1653	2.9395	3.2105	3.4823	3.7432	2.6799	3.5823	3.1307	3.2727	3.1497	3.6466	3.6193	3.3084
3.4774	2.7723	3.0259	3.4143	1.9657	3.1926	2.8833	3.4211	3.2218	2.8918	3.4541	3.5333	3.6216	3.1112	3.444	3.6417	3.5187
3.1076	2.5772	1.9793	3.4077	1.1864	2.7742	2.2446	2.9538	3.0648	2.3063	3.0912	2.6306	2.7797	2.8009	3.244	3.3177	2.6271
[illegible]																

M1C 12 pcw	M1C 12 pcw	M1C 12 pcw	M1C 13 pcw	M1C 13 pcw	M1C 13 pcw	M1C 16 pcw	M1C 21 pcw	M1C 24 pcw	M1C 37 pcw
12835	12960	13060	12820	12834	12888	12837	12886	12288	3E+08
4.7794	5.0214	4.6504	4.9182	4.7747	4.7777	5.1687	4.4556	4.1508	3.1599
4.7737	4.8151	4.3972	4.8522	4.7391	4.4762	5.0544	4.4914	3.9754	3.438
4.757	5.0486	4.0764	4.7002	5.1031	4.7705	5.2209	4.4565	3.9225	3.3936
[illegible]									

M1C 4 mos	M1C 4 mos	M1C 1 yrs	M1C 3 yrs	M1C 3 yrs	M1C 8 yrs	M1C 11 yrs	M1C 13 yrs	M1C 18 yrs	M1C 19 yrs	M1C 21 yrs	M1C 23 yrs	M1C 30 yrs	M1C 36 yrs	M1C 37 yrs	M1C 40 yrs
12889	12890	12830	12836	12980	12841	12289	12831	12984	12832	13057	12300	12290	12302	12303	12304
3.2547	2.889	3.3846	2.5169	2.5867	2.6893	3.319	3.716	2.4682	3.4701	3.3845	3.0608	3.1153	3.3606	3.6848	2.3721
3.5214	2.9818	3.289	2.8466	2.9245	2.3455	3.1307	3.296	2.7578	3.3276	3.5357	3.4612	3.0567	3.2407	3.5117	2.2705
2.9908	2.9257	3.2982	2.5227	2.5341	1.4398	2.8331	3.0216	2.0437	3.1236	2.8662	2.6788	2.7006	3.0218	3.1898	1.6103
[illegible]															

[illegible] 8 pcw	[illegible] 9 pcw	[illegible] 16 pcw	[illegible] 17 pcw	[illegible] 19 pcw	PCx 8 pcw	PCx 9 pcw
13058	12833	12879	12880	12885	13058	12833
4.2008	4.5779	4.6829	4.2641	4.2383	3.8887	4.7119
4.42	4.8426	4.3282	4.2525	3.0822	4.0687	4.8313
4.1944	4.5944	4.3612	4.2652	3.0932	3.9192	5.0161
[illegible]						

S1C 12 pcw	S1C 12 pcw	S1C 12 pcw	S1C 13 pcw	S1C 13 pcw	S1C 13 pcw	S1C 16 pcw	S1C 21 pcw	S1C 24 pcw	S1C 37 pcw	S1C 4 mos	S1C 10 mos
12835	12960	13060	12820	12834	12888	12837	12886	12288	3E+08	12890	12977
4.8311	4.4922	4.4619	4.7939	4.7635	4.5955	5.2141	4.5289	4.5412	3.1819	2.9584	3.162
4.6574	4.3634	4.2308	4.6658	4.7664	4.3239	4.893	4.4205	4.464	3.4577	3.1289	2.8325
4.7028	4.3994	3.9309	4.6953	5.1721	4.5104	5.1724	4.4967	4.6474	3.3813	3.208	1.4409
[illegible]											

S1C 1 yrs	S1C 2 yrs	S1C 3 yrs	S1C 8 yrs	S1C 11 yrs	S1C 13 yrs	S1C 18 yrs	S1C 19 yrs	S1C 21 yrs	S1C 23 yrs	S1C 30 yrs
12830	12979	12980	12841	12289	12831	12984	12832	13057	12300	12290
3.6996	2.646	2.6789	2.5829	3.3481	3.6737	2.6171	3.7142	3.4019	3.2202	3.1146
3.6084	2.7203	3.1576	2.3493	3.198	3.42	2.7846	3.6027	3.6911	3.5702	3.1973
3.4671	1.8087	2.4705	1.3412	2.7288	2.9217	2.0875	3.0473	2.9001	2.6994	2.7173
[illegible]										

S1C 36 yrs	S1C 37 yrs	S1C 40 yrs	IPC 12 pcw	IPC 12 pcw	IPC 12 pcw	IPC 13 pcw	IPC 13 pcw	IPC 13 pcw	IPC 16 pcw	IPC 16 pcw	IPC 16 pcw	IPC 17 pcw	IPC 19 pcw	IPC 21 pcw	IPC 24 pcw	IPC 37 pcw	IPC 4 mos	IPC 10 mos	IPC 1 yrs
12302	12303	12304	12835	12960	13060	12820	12834	12888	12287	12837	12879	12880	12885	12886	12288	3E+08	12890	12977	12830
3.575	3.5112	2.3987	4.8441	4.6914	3.626	5.0235	4.4832	4.7023	4.7171	5.2289	4.6957	4.6494	4.0343	4.4075	4.419	3.4184	3.0401	3.4031	3.6816
3.4388	3.3157	2.3966	4.8727	4.513	3.5662	4.9842	4.4917	4.5805	4.3207	4.8146	4.5558	4.5023	3.1927	4.2872	3.791	3.6474	3.125	3.0088	3.5573
3.0689	3.1599	1.92	4.7973	4.3116	2.5573	4.9023	4.7955	4.7985	4.2773	5.1287	4.7373	4.4701	3.1438	4.4437	4.155	4.0289	3.2454	1.9182	3.5417
3.2873	2.9868	1.8987	4.8061	4.3216	3.0671	4.877	4.457	4.6355	4.3314	4.711	4.644	4.3526	3.086	4.1498	3.5745	3.3681	2.9643	2.2064	3.5416
2.5386	2.1961	1.1938	4.42	4.279	3.3998	4.2527	3.9358	4.1991	3.9636	4.4491	4.2041	3.9367	2.7782	3.7931	3.8357	2.8265	2.5566	2.1769	2.6107
2.6687	2.8351	1.579	4.0454	3.3867	2.8891	4.2918	3.6819	3.9427	3.7179	3.9726	3.8592	4.0931	2.3537	3.8288	3.2782	2.7967	2.501	2.3076	3.0695
1.7359	1.691	0.7647	3.0729	2.0798	1.48	3.0831	2.5639	3.1659	3.1497	3.0887	2.8644	2.7982	1.5901	2.4142	1.6423	1.4907	1.2236	1.2753	1.5816
2.8153	2.8886	2.1412	3.7534	3.7166	3.0153	3.9708	3.6017	3.561	3.383	3.7457	3.7645	3.5294	2.4453	3.595	3.1462	3.0344	2.5781	2.7057	2.9447
1.4003	1.3332	0.7794	2.5882	2.2563	1.8403	2.6473	2.4284	2.1054	2.1016	2.4563	2.3314	2.1385	1.3326	1.9558	1.7996	1.5556	1.0768	1.0758	1.6779
2.5349	2.2012	1.1131	4.1097	4.024	3.1378	4.107	3.6806	4.0218	3.7553	4.2063	3.9663	3.9264	3.064	3.6879	3.5564	2.785	2.209	1.8301	2.3888
3.9249	3.9287	2.3548	4.9824	4.6534	4.0296	4.9961	4.2016	4.1271	4.8067	5.2653	4.9199	4.9626	4.6097	4.6224	4.6044	3.9523	3.3059	3.3196	3.9579
3.6444	3.7912	1.8601	4.5764	4.1403	3.2121	4.6218	4.4052	4.0285	4.141	4.4822	4.1477	4.1606	3.1128	3.888	3.7798	3.1009	2.4986	2.4961	2.9446
2.733	2.3596	1.4215	4.9344	4.8565	3.6316	5.0466	4.5531	3.8195	4.0699	4.7176	4.5128	4.2688	2.6718	3.9031	3.6494	3.0085	2.3555	2.2284	2.9368
2.0636	1.7721	1.0465	4.4075	4.1082	2.4062	4.7022	3.9151	4.5162	3.7942	4.3497	4.2223	4.2087	2.8229	3.7887	3.3611	2.7508	2.3827	1.6832	2.6727
3.8883	3.9466	2.6502	5.0956	5.0887	4.3022	5.4778	5.0446	4.9481	4.9841	5.743	5.1563	5.2778	3.4937	5.0308	4.9248	3.9799	3.5743	2.8982	3.7772
1.9543	1.9149	1.4596	3.2728	3.1551	2.9137	3.4145	3.0861	2.7864	3.0794	3.1677	3.2119	3.3306	2.5859	3.1249	2.8703	1.884	1.7866	1.3761	2.2959
2.7606	2.673	1.3466	4.3348	4.0266	2.222	4.3267	4.0693	3.8006	3.9827	4.3603	4.452	3.5103	3.2364	3.465	2.6211	2.3497	1.9627	1.7911	2.0364
3.4668	3.3175	2.2053	4.4821	4.1075	3.1802	4.3332	4.2006	4.2576	4.0561	4.2605	4.0382	3.7108	2.4844	3.6465	3.5061	3.2946	2.602	2.4703	2.8398
3.2531	3.1982	2.4184	4.0066	3.3552	2.7801	3.9747	3.6435	3.6026	3.7966	3.8938	3.7128	3.6271	2.6912	3.3795	3.0764	3.046	2.2597	2.3193	3.0169
3.2228	3.43	2.0238	4.465	3.9693	3.6488	4.6564	4.4753	4.1621	3.9918	4.2755	4.2295	3.9879	2.7532	4.1048	3.6538	3.1503	2.872	3.0511	3.3531
2.0308	1.8384	1.1816	4.4649	4.131	3.2005	4.586	4.101	4.2243	3.5446	3.984	3.8457	3.73	2.5135	3.5154	2.8355	2.5356	1.9595	1.2531	2.1242
0.9742	0.9403	0.9604	2.9169	2.1092	2.0664	2.8743	2.2693	2.5953	2.6976	2.252	2.214	2.8023	1.7123	2.5083	1.8003	1.2881	0.7392	0.9449	1.3629
1.1398	0.9635	0.7484	2.7176	2.2726	2.1593	2.9623	2.4819	2.302	2.5711	2.8694	3.0875	2.7906	2.0988	2.7485	2.3127	1.6006	1.0133	0.7343	1.1697
2.4067	2.4792	1.4802	3.4552	3.1458	2.0826	3.4998	3.4491	3.374	3.0631	3.2629	3.5166	3.0519	1.9637	3.2658	2.5458	2.427	2.2586	1.7577	2.528
3.1253	2.9252	1.4626	4.5853	4.5274	3.0224	4.7551	4.399	4.1029	3.9676	4.6902	4.0834	3.7208	2.6798	3.4969	3.2268	3.14	1.9148	1.3654	2.5743
2.2339	1.9378	1.3425	5.11	4.9578	4.0642	5.1658	4.6106	4.4365	4.5318	5.1261	4.6887	4.6118	3.1203	4.6204	4.4685	3.8305	2.6734	2.1689	3.2575
2.267	2.6296	1.5393	4.6597	4.3955	3.5864	4.5799	4.6633	4.4504	4.2258	4.7655	4.6927	4.25	3.1559	4.144	3.9963	3.3199	2.4415	1.7657	2.4671
3.3585	3.3212	1.6292	4.9799	4.4901	2.9563	5.1465	4.97	5.0847	4.5612	5.1855	5.3179	4.4913	3.5458	4.6798	4.0559	3.7131	3.2189	2.2503	3.5225
1.3163	1.5215	0.8288	3.1456	3.0233	2.0359	3.3227	3.4005	3.3754	3.0755	3.5952	3.8799	3.004	2.4142	3.1982	2.7677	2.6424	1.7101	1.5811	1.6657
5.2915	5.3611	3.9468	6.5003	6.3902	4.4763	6.5678	6.4067	6.242	6.2511	6.7387	6.6295	6.0895	5.382	5.9705	5.6367	5.2644	5.1899	4.5007	5.4664
3.4027	3.0819	1.886	4.5763	3.904	2.2273	4.8467	4.4128	4.4507	4.2348	4.7615	4.9846	4.1505	3.4664	4.1983	3.6621	3.4682	3.0736	2.3981	3.3637
3.564	3.5672	2.3304	4.4193	3.8931	3.365	5.032	4.2858	4.9787	4.0484	4.6627	4.7749	4.4459	3.4613	4.5314	3.9844	3.2837	3.1376	3.1801	3.5882
2.706	2.3788	1.5296	3.32	2.898	2.1068	3.7701	2.8965	3.5361	2.8274	3.3125	3.2495	3.2217	3.1718	2.8629	2.7759	2.0013	1.8716	2.0714	2.813
1.5377	1.6061	0.7266	2.4827	2.2392	1.4024	2.4215	2.1035	2.1296	2.4217	2.7443	1.7345	1.9865	1.3408	1.6498	1.481	1.628	0.9608	0.7253	1.3241
3.2254	3.6402	2.4096	4.9779	4.8495	4.6342	5.0766	5.1876	4.8295	4.8828	4.9605	4.8455	4.6306	3.3874	4.7692	4.7374	3.4734	3.131	3.4131	3.3697
3.5906	3.6775	2.9564	4.1953	4.0169	3.8233	4.1704	4.2707	4.0707	4.0927	4.1573	4.1897	3.9264	3.0967	4.0411	4.0393	3.5393	3.0654	2.7384	3.5816
2.2491	2.125	1.5078	3.1136	3.1333	2.4894	2.8195	2.961	3.2158	3.131	2.7676	2.6964	2.9847	2.3441	2.6661	2.8043	2.1326	1.7642	1.9844	2.1963
1.2547	1.7158	0.7245	3.0849	3.2085	1.9543	3.1472	2.7136	2.6461	2.6026	3.2665	2.346	2.6646	2.3541	2.0502	2.2347	1.7367	1.5289	0.8865	1.3452
2.526	2.8341	2.0422	4.7993	4.5534	3.1641	4.7303	4.7539	4.6634	4.7452	5.4022	4.8278	4.5952	4.1093	4.6519	4.3539	4.2957	3.5286	2.6054	3.1603
1.4038	1.7946	0.1774	4.6155	4.7667	3.7595	4.5289	5.0982	3.9781	4.2293	4.5926	3.9536	3.9292	0.3462	3.9009	3.8798	2.731	2.0627	2.0075	2.4759
2.7209	2.6663	1.9377	4.5197	4.2028	3.0582	4.8714	4.0733	3.82	4.5724	4.886	5.0009	4.772	3.2022	4.8469	4.5822	3.8599	3.3556	2.6056	3.2281
2.199	2.0367	1.1953	2.9347	2.8474	2.2716	3.1235	2.5217	2.6167	2.8489	3.2146	2.7506	3.0456	2.2517	2.9067	2.6522	1.8462	1.8458	1.3065	2.1923

STC 30 yrs	STC 36 yrs	STC 37 yrs	STC 40 yrs	ITC 8 pcw	ITC 12 pcw	ITC 12 pcw	ITC 12 pcw	ITC 13 pcw	ITC 13 pcw	ITC 13 pcw	ITC 16 pcw	ITC 16 pcw	ITC 21 pcw	ITC 21 pcw	ITC 24 pcw	ITC 37 pcw	ITC 4 mos	ITC 4 mos	ITC 4 mos
12290	12302	12303	12304	13058	12835	12960	13060	12820	12834	12888	12837	12879	12365	12886	12288	3E+08	12296	12889	12890
3.1165	3.3204	3.455	3.1683	3.8204	4.2809	4.5786	3.8976	4.8214	4.102	4.1695	4.7402	4.1938	4.3184	4.4431	4.5802	3.3972	3.4739	3.1317	2.8307
3.2703	3.5188	3.3064	3.612	4.0473	4.3781	4.5651	3.7964	4.7255	4.1169	3.9436	4.4555	3.993	4.3515	4.5922	4.5056	3.6731	3.8138	3.1146	3.2624
2.7733	3.0219	3.1318	2.5765	3.7859	4.3187	4.2676	3.0653	4.6956	4.405	3.6526	4.8469	3.9084	4.2161	4.5583	4.7235	4.1642	3.1818	2.7401	3.0947
3.0208	2.8943	3.3787	2.929	3.901	4.4206	4.4941	3.4944	4.5376	4.2954	3.8967	4.5805	4.1309	4.3545	4.3469	4.0084	3.5234	3.1619	1.9775	3.1283
1.8585	2.2387	2.3196	2.1315	3.7281	3.8612	4.27	3.6082	4.1386	3.8051	3.7835	4.4867	4.0311	4.0616	4.0573	3.7295	2.9767	2.5135	2.2004	2.0434
2.3323	2.712	2.5303	2.7472	3.2233	3.4729	3.3242	3.0939	4.102	3.2148	3.103	3.7605	3.4152	3.846	3.9861	3.6928	2.8102	2.9536	2.617	2.6806
1.3438	1.2689	1.6574	1.0569	1.8651	2.1519	2.1468	1.6064	3.2346	2.3254	2.2404	2.9967	2.0688	2.6267	2.4715	2.1239	1.6276	1.8455	1.6513	1.0768
2.5941	2.7329	2.9428	2.9666	3.597	3.6786	3.7561	3.2226	3.7893	3.3745	3.3584	3.6445	3.4224	3.1978	3.7103	3.5135	2.9792	2.7934	2.2812	2.4811
0.9776	1.3542	1.3839	1.2258	2.2983	2.4646	2.413	2.0807	2.6077	2.2228	1.7275	2.4067	2.0877	2.0013	2.1797	2.2276	1.2625	1.415	1.5552	1.1802
1.629	2.0924	2.2059	1.9051	3.4376	3.6605	3.9933	3.2633	3.9389	3.5904	3.5111	4.2549	3.7914	3.8305	3.874	3.6135	2.9307	2.5637	2.3417	2.1951
3.0607	3.5412	3.666	3.2312	4.1145	4.297	4.3134	3.9635	4.8867	3.8511	3.7502	4.773	4.3979	4.8946	4.6596	4.8608	4.0086	3.488	3.3289	3.39
2.8229	3.399	3.3772	2.8808	3.859	4.1564	4.2389	3.6393	4.4677	4.2405	3.6027	4.4893	3.7342	4.4105	3.9269	4.0581	3.2061	2.7481	3.251	2.5476
2.2445	2.5684	2.8469	2.5657	3.5855	4.5707	5.2081	4.0285	4.7142	4.4131	3.5958	4.3876	3.9255	3.53	4.163	3.8545	2.9648	2.6285	2.4111	2.4119
1.6412	1.9574	2.0003	1.9819	3.6441	3.6747	3.9714	2.7795	4.7349	3.3749	3.5737	3.9183	3.3125	3.7413	4.0127	3.7162	2.8821	2.4933	1.8123	2.3815
3.586	3.722	3.9246	3.6928	4.6535	5.0774	5.1772	4.7593	5.0389	5.1212	4.6889	5.3074	5.1392	4.8736	5.0666	5.105	4.2033	3.8179	3.3035	3.6211
1.8457	1.9651	2.0718	1.9677	2.9489	3.0823	3.129	2.9691	3.2727	2.9279	2.7434	3.058	3.1368	2.6996	3.2259	2.9395	1.8183	1.9943	1.9085	1.8404
2.5319	2.4395	2.8108	2.137	3.3254	3.7669	4.0601	2.7659	4.7068	4.0185	3.0857	4.5545	3.4473	3.9716	3.3689	3.2124	2.7817	1.1544	1.5811	2.0911
3.0304	3.0923	3.2547	2.6196	3.7814	4.0255	4.1046	3.5188	4.4999	4.0195	3.7019	4.0865	3.5517	3.9598	3.7664	3.8695	3.4353	2.5721	2.8356	2.4677
2.9729	3.1334	3.0543	2.879	3.1759	3.5204	3.3968	3.1313	3.8609	3.5031	3.0613	3.858	3.3249	3.9612	3.4448	3.2976	3.0966	2.625	2.6336	2.3321
3.0146	3.1704	3.3069	3.0858	4.2212	4.1729	4.0655	3.8471	4.5258	4.2339	3.8578	4.2113	4.1306	4.3429	4.1103	3.7988	3.1224	3.0115	2.6274	2.896
1.7111	1.9846	1.9609	2.1154	3.2075	3.5648	4.1328	3.401	4.3522	3.5341	3.2156	3.5207	3.2024	3.0808	3.6628	3.2304	2.6271	2.4474	2.0486	2.041
0.7252	1.0832	1.0063	1.1501	2.0025	2.7493	2.0963	2.3043	2.5398	1.9405	2.0577	2.4154	2.1189	2.2858	2.5592	2.2117	1.2385	1.3829	1.2681	0.9987
0.952	0.9166	0.9833	0.9563	1.2676	2.066	2.2023	2.1928	2.4532	2.1937	1.8386	2.0904	2.1773	2.0023	3.0732	2.4262	1.5674	1.3433	0.72	1.1108
2.1997	2.2776	2.7231	2.327	3.0002	3.0225	3.2076	2.3014	3.6119	2.9995	2.6129	3.5132	2.9419	3.2944	3.2773	2.9071	2.5732	2.3028	1.895	2.1997
2.5477	2.76	2.9777	2.5481	3.6722	4.1352	4.7502	3.5116	4.7567	4.2018	3.7703	4.635	3.5221	3.8047	3.4836	3.5261	3.2701	1.9388	2.0367	2.0354
2.1215	2.0658	2.1849	1.9825	4.143	4.7853	4.8723	4.185	4.6782	4.3943	3.8988	5.095	4.8781	4.4015	4.7394	4.8996	3.9586	3.0878	2.5677	2.8744
2.2751	2.2724	2.6732	2.1959	4.2852	4.562	4.5966	3.9163	4.6156	4.4894	4.0462	4.7248	4.2595	4.321	4.0878	4.6203	3.6972	2.3241	2.2967	2.6214
3	3.1824	3.4568	2.7545	4.5635	4.7175	4.6239	3.5879	5.2079	4.8752	4.362	5.0577	4.4084	4.6972	4.5972	4.4875	3.8537	2.8277	2.9277	3.2494
1.5094	1.3899	1.5936	1.3744	2.5661	2.5769	2.9802	2.1973	3.2407	2.9243	2.5841	3.1247	2.9444	2.7553	3.239	2.9698	2.6244	1.3205	1.1288	1.8052
5.3841	5.3796	5.6018	5.1021	5.6258	6.0044	6.4082	5.0039	6.8053	6.1323	5.2588	6.9081	5.9416	6.0901	6.015	5.9367	5.6436	4.7727	4.2456	5.1945
3.0608	2.9535	3.4256	2.9715	3.6437	4.1675	4.0642	2.8989	4.9294	4.2791	3.5561	4.6794	3.8408	4.0349	4.2236	4.109	3.6127	2.6678	2.8986	3.1051
3.1474	3.4637	3.4431	3.6558	4.0559	4.1485	4.0054	3.735	4.834	4.0923	4.1825	4.5784	4.3806	4.6393	4.5397	4.2299	3.2879	3.7705	2.5816	3.2738
2.037	2.3744	2.3702	2.4664	3.1352	3.083	2.9959	2.5603	3.5967	2.7478	3.1515	3.4319	2.922	2.9283	2.9948	2.9085	1.9366	2.4851	1.7659	1.8276
0.8568	1.2081	1.2754	1.0504	1.7266	2.0159	2.2624	1.6026	2.3484	1.8967	1.8112	2.5935	1.6221	1.7435	1.6734	1.6836	1.6315	1.2793	1.564	1.0396
3.137	3.3261	3.3285	3.0769	4.8765	4.8882	4.949	4.7758	4.9721	5.0372	4.6117	4.7549	4.741	4.5893	4.6113	4.5936	3.5951	3.0508	3.0012	3.0746
3.4659	3.4547	3.7637	3.1602	4.0738	3.9863	4.0168	4.029	4.1877	3.9733	3.8354	4.0448	4.0407	3.8239	4.0648	4.1451	3.6651	2.8884	3.0131	3.0191
1.8995	2.0112	1.9644	1.7317	2.42	2.587	3.1332	2.492	2.8239	2.6474	2.6898	2.9346	2.3783	3.2613	2.7945	2.8657	2.2651	2.27	1.839	1.6738
1.1143	1.1779	1.283	1.1091	2.7612	2.6237	3.1799	2.1901	3.1163	2.5174	2.295	2.8895	2.4397	2.6596	1.7884	2.7645	1.602	1.2663	1.3214	1.5055
2.9077	2.9441	2.9508	2.3183	4.11	4.4098	4.4915	3.5737	4.7309	4.3629	3.8238	5.1288	4.1764	4.4296	4.8052	4.7256	4.5862	3.5765	3.4036	3.6485

[illegible]

HIP 23 yrs	HIP 30 yrs	HIP 36 yrs	HIP 37 yrs	HIP 40 yrs	AMY 8 pcw	AMY 9 pcw	AMY 12 pcw	AMY 12 pcw	AMY 12 pcw	AMY 13 pcw	AMY 13 pcw	AMY 13 pcw	AMY 16 pcw	AMY 16 pcw	AMY 17 pcw	AMY 21 pcw	AMY 24 pcw	AMY 37 pcw	AMY 4 mos
12300	12290	12302	12303	12304	13058	12833	12835	12960	13060	12820	12834	12888	12837	12879	12880	12886	12288	3E+08	12296
3.2776	3.0496	3.1533	3.5233	2.2822	3.7229	4.2067	3.9216	3.9903	3.7044	4.9527	4.2534	4.1002	4.6804	4.4082	4.2322	4.1944	4.5071	3.0952	3.0999
3.4666	2.8043	3.1221	3.3499	2.4003	3.8617	4.5419	4.0347	4.2415	3.8396	4.8023	4.5589	4.2228	4.285	4.4076	4.3319	4.44	4.5105	3.376	3.2778
2.8247	3.0133	2.9396	3.3677	1.9241	3.5502	4.5292	4.082	4.1043	3.6494	4.6909	4.8479	4.2828	4.6131	4.2441	4.2011	4.2471	4.5964	2.9312	2.9754
2.6083	3.0682	2.8988	3.2518	1.6013	4.0368	4.0711	3.7123	3.8153	3.6283	4.5999	4.4176	4.0111	3.3182	4.5027	4.1423	4.3536	3.4283	2.2673	3.1552
1.8911	1.6559	2.2768	2.4158	0.3482	3.7219	4.0634	3.5662	3.8645	3.7327	4.3003	4.1429	3.9631	3.5966	4.1294	3.9549	3.765	3.6592	2.2068	2.3307
2.7063	2.2209	2.5628	3.0204	1.1105	3.1438	2.4543	3.2383	3.1048	3.3638	4.2186	3.8952	3.5892	3.2169	4.0357	3.702	3.9453	3.6624	2.3511	2.6613
1.4187	1.1831	1.3595	1.805	0.637	1.6851	2.5104	1.8906	1.934	1.6584	3.2724	2.821	2.7787	1.9288	2.2325	2.7295	2.6719	2.2931	1.2433	1.6474
2.7942	2.3519	2.5607	2.891	1.8407	3.3594	3.8049	3.5778	3.4958	3.2214	3.9394	3.6876	3.591	3.3679	3.8139	3.4569	3.6924	3.8578	2.5582	2.5774
1.295	0.8568	1.1535	1.5678	0.8032	2.1466	2.3872	2.3932	2.2903	2.0837	2.648	2.7615	2.1278	2.0867	2.4966	2.3928	2.307	2.5044	1.1986	1.2357
2.062	1.6118	2.0997	2.3834	0.8776	3.44	3.7805	3.3394	3.7091	3.4657	4.0529	3.7909	3.756	3.3742	3.9372	3.7433	3.7686	3.2905	2.2451	2.2448
3.3203	3.0784	3.6844	3.847	3.2622	4.1939	4.0469	4.2874	4.3289	4.0162	4.9238	4.3563	4.088	4.0213	4.5845	4.6317	4.4719	4.7403	3.4015	3.4831
2.9907	2.8544	3.1862	4.0284	1.7234	3.6979	4.4947	4.0889	3.8428	3.7051	4.4741	4.5484	3.8165	3.8759	4.1285	4.21	4.0394	4.1134	3.227	2.6152
2.5297	2.0174	2.6088	2.9051	1.4434	3.3351	3.6177	3.1779	3.5558	3.0632	4.7306	3.7957	2.628	2.631	3.9269	3.5817	3.8769	3.133	2.5893	2.4034
1.3515	1.1071	1.2499	1.671	0.9329	3.3026	4.423	3.5793	3.5037	3.2655	4.8342	4.0452	4.0381	3.6791	4.0611	4.118	3.9764	4.1898	1.552	1.7703
3.1745	3.4059	3.513	3.872	1.8403	4.7203	4.8501	4.6379	4.8613	4.671	5.0717	5.2153	4.5381	4.6402	5.0326	5.2321	5.0858	5.2859	3.3419	3.5477
1.6046	1.4652	1.8607	1.8932	1.1835	2.95	2.7902	2.9168	2.91	2.6936	3.1999	2.8582	2.5849	2.56	3.079	2.9338	3.0783	2.7457	1.3765	1.6475
1.357	2.3739	2.1241	2.1332	1.0198	3.191	3.5625	3.341	3.7166	2.9764	4.4979	4.1188	3.0001	3.5917	3.6516	3.8424	3.5548	3.4195	1.473	2.2321
2.7878	2.7951	2.8437	3.1073	1.7246	3.8155	4.5001	4.0487	3.8753	3.5156	4.4128	4.6031	4.1212	4.0809	3.9217	4.1826	3.8644	4.5847	3.1796	2.8177
2.512	2.6745	2.6407	3.0484	1.7318	3.107	3.2846	3.3315	3.0637	2.971	3.8388	3.9329	3.2571	3.402	3.5314	3.8251	3.4852	3.4572	3.1879	2.6002
3.2064	2.9703	2.9283	3.3252	1.3991	4.1372	4.1234	4.0103	3.7443	3.6802	4.5214	4.3046	3.6886	3.5232	4.2634	4.0094	4.1884	3.871	2.7173	3.0973
2.2085	1.7102	2.0632	2.0556	1.1643	3.0983	3.6824	3.4571	3.1662	3.142	4.431	4.0139	3.4767	3.0732	3.5687	3.8136	3.5667	3.5588	2.025	2.308
0.9536	0.6553	1.2593	1.0918	0.5775	1.9448	2.1551	2.2185	1.7346	1.8274	2.7078	2.3273	2.2247	1.7582	2.6236	2.695	2.6431	2.4624	0.9501	1.0406
0.7534	0.8551	0.8454	1.0388	0.314	1.1635	1.1554	1.0545	1.0702	1.0084	2.3103	1.6622	1.322	1.0713	1.7693	1.7973	2.1154	1.3183	0.6581	0.9391
2.2758	2.3866	2.4939	2.4216	1.3528	3.2579	3.3492	3.1152	3.0651	2.8072	3.6203	3.86	3.1939	2.7839	3.3864	3.7134	3.3824	3.1931	2.0642	2.4685
2.5753	2.2276	2.4453	2.7622	1.2998	3.4598	4.129	3.6858	3.5884	3.0571	4.7186	4.1739	3.3542	3.9809	3.9103	4.3242	3.8355	4.0113	3.285	2.2001
2.1971	1.6478	1.8261	2.5566	0.8281	4.2043	4.3769	3.9974	4.5252	4.1042	4.7745	4.4072	3.9657	4.046	4.8184	4.7704	4.8242	4.1346	2.6277	2.1409
2.2813	2.4952	2.603	2.6474	1.2629	4.0348	4.2484	4.1517	4.19	3.9134	4.5893	4.4741	3.8809	4.3517	4.2203	4.2414	4.2772	4.2296	2.7606	2.6565
3.2588	3.1156	3.0281	3.257	1.5709	4.5308	5.2354	4.8406	4.5478	4.2217	5.1614	5.2888	4.8759	4.539	4.7404	4.7766	4.5901	4.4706	3.2055	3.3646
1.4602	1.4007	1.2643	1.1338	0.457	2.3864	3.0533	2.4363	2.6946	2.0368	3.1296	3.0132	2.6643	2.4848	2.6659	2.7937	2.7701	2.7244	1.6729	1.6613
4.6927	5.581	5.4782	5.254	3.1019	5.4319	5.9902	5.6348	6.132	5.4781	6.6394	6.3312	5.7202	5.891	6.1699	6.0142	6.1176	5.7136	4.5356	5.4139
2.9907	2.7885	2.8276	2.96	2.1819	3.3475	4.3982	4.1995	3.9199	3.6941	4.8776	4.7801	4.2515	4.1822	4.2817	4.5104	4.2546	4.2038	2.9586	3.1374
3.2305	2.9152	3.1367	3.4794	1.782	4.0256	4.2509	4.0455	3.7566	3.9737	4.9245	4.3479	4.4993	4.2299	4.7104	4.3204	4.4486	4.4016	2.6813	3.5124
2.5073	1.939	2.1923	2.6151	1.4824	2.944	3.2598	2.83	2.72	2.8043	3.7352	3.1393	3.4845	2.7133	3.308	3.1013	3.0548	2.9888	1.675	2.2918
1.1612	0.7792	1.1075	1.6078	0.3061	1.6429	1.9451	1.963	1.9375	1.61	2.4749	1.9565	1.8283	2.6458	1.8841	1.9972	1.9038	1.9136	1.4541	1.1371
2.9696	3.1678	3.0222	3.3469	1.4472	4.9166	4.9352	5.0395	4.8147	4.6701	4.9737	5.0589	4.7407	4.4151	4.6992	4.5683	4.646	4.789	3.0576	3.0753

[illegible]

MD 40 yrs	URL 8 pcw	URL 9 pcw	CB 12 pcw	CB 13 pcw	CB 13 pcw	CBC 12 pcw	CBC 16 pcw	CBC 17 pcw	CBC 21 pcw	CBC 21 pcw	CBC 24 pcw	CBC 35 pcw	CBC 37 pcw	CBC 4 mos	CBC 4 mos	CBC 10 mos	CBC 1 yrs	CBC 2 yrs	CBC 3 yrs	CBC 3 yrs	CBC 4 yrs	CBC 8 yrs	CBC 8 yrs	CBC 11 yrs	CBC 13 yrs	CBC 15 yrs	CBC 18 yrs	CBC 19 yrs	CBC 21 yrs	CBC 23 yrs	CBC 30 yrs	CBC 36 yrs	CBC 37 yrs	CBC 40 yrs H376.XI.56
12304	13058	12833	12960	12834	12888	13060	12837	12880	12365	12886	12288	12295	[illegible]	12296	12890	12977	12830	12979	12836	12980	12298	12841	12981	12289	12831	12299	12984	12832	13057	12300	12290	12302	12303	12304
[illegible]	[illegible]	[illegible]	[illegible]	[illegible]	[illegible]	[illegible]	[illegible]	[illegible]	[illegible]	[illegible]	[illegible]	[illegible]	[illegible]	[illegible]	[illegible]	[illegible]	[illegible]	[illegible]	[illegible]	[illegible]	[illegible]	[illegible]	[illegible]	[illegible]	[illegible]	[illegible]	[illegible]	[illegible]	[illegible]	[illegible]	[illegible]	[illegible]	[illegible]	[illegible]

Supplemental Table 2

Top 10 clusters with their representative enriched term. "Count" is the number of genes in the list of genes with developmental expression correlated to GATAD2B (excluding GATAD2B) with membership in the given ontology term. "%" is the percentage of all of the user-provided genes that are found in the given ontology term (only input genes with at least one ontology term annotation are included in the calculation). "Log10(P)" is the p-value in log base 10. "Log10(q)" is the multi-test adjusted p-value in log base 10.

GO	Category	Description	Count	%	Log10(P)	Log10(q)
GO:0016569	GO Biologi	covalent chromatin modifi	19	20.00	-14.60	-10.40
GO:0016573	GO Biologi	histone acetylation	8	8.42	-7.24	-3.95
GO:0006338	GO Biologi	chromatin remodeling	7	7.37	-5.83	-2.91
GO:0010452	GO Biologi	histone H3-K36 methylatic	3	3.16	-4.78	-1.99
GO:0007420	GO Biologi	brain development	11	11.58	-4.33	-1.61
GO:0071824	GO Biologi	protein-DNA complex sub	6	6.32	-3.68	-1.09
GO:0030218	GO Biologi	erythrocyte differentiation	4	4.21	-3.12	-0.59
GO:0035107	GO Biologi	appendage morphogenesi	4	4.21	-2.89	-0.41
GO:0016055	GO Biologi	Wnt signaling pathway	6	6.32	-2.41	-0.02
GO:0061564	GO Biologi	axon development	6	6.32	-2.30	0.00

Gene	p_val	avg_log2F(	pct.1	pct.2	p_val_adj
Hist1h1e	0	0.687	0.436	0.65	0
Fos	0	0.465	0.183	0.372	0
AC149090.	0	0.446	0.389	0.624	0
Hnrnpa0	0	0.430	0.875	0.95	0
H3f3b	0	0.375	0.963	0.985	0
Cbx3	0	0.328	0.851	0.935	0
Actb	0	0.326	0.986	0.991	0
Slc24a5	0	0.317	0.68	0.827	0
Chchd2	0	0.284	0.892	0.947	0
Atp5k	0	-0.277	0.838	0.878	0
Rps28	0	-0.353	0.958	0.965	0
Rps29	0	-0.356	0.982	0.98	0
Rpl38	0	-0.379	0.972	0.97	0
Uba52	0	-0.436	0.846	0.869	0
Gm10076	0	-0.467	0.871	0.886	0
Ttr	0	-1.892	0.163	0.001	0
Gatad2b	3.55E-320	-0.305	0.666	0.638	7.76E-316
Jund	1.22E-307	0.320	0.803	0.9	2.67E-303
Marcks	3.00E-306	0.269	0.903	0.957	6.55E-302
Cdk2ap1	1.48E-256	0.287	0.426	0.57	3.24E-252
Frmd4a	3.45E-214	-0.311	0.709	0.732	7.55E-210
H2afv	6.68E-210	0.256	0.749	0.857	1.46E-205
H1f0	5.78E-197	0.264	0.551	0.682	1.26E-192
Mapt	1.36E-195	-0.315	0.87	0.855	2.98E-191
Nfia	9.64E-181	0.409	0.548	0.646	2.11E-176
Mdk	5.89E-166	0.358	0.448	0.569	1.29E-161
Gria2	1.03E-161	-0.360	0.863	0.874	2.25E-157
Ptprk	1.38E-145	-0.323	0.384	0.37	3.01E-141
Itpr1	2.01E-143	-0.263	0.333	0.301	4.39E-139
Hmgb2	1.08E-140	0.336	0.454	0.571	2.36E-136
Ptprd	2.09E-128	-0.300	0.73	0.745	4.57E-124
Grin2b	2.22E-123	-0.343	0.623	0.582	4.85E-119
Gsdme	1.60E-117	-0.277	0.508	0.498	3.50E-113
Arpp21	6.93E-97	-0.372	0.481	0.453	1.51E-92
Mef2c	1.10E-94	-0.408	0.741	0.695	2.40E-90
Malat1	2.17E-93	-0.342	0.998	0.995	4.73E-89
Nrxn1	2.21E-81	-0.287	0.696	0.685	4.82E-77
Ntrk2	1.59E-76	-0.255	0.488	0.483	3.48E-72
Meg3	2.00E-72	-0.264	0.716	0.76	4.37E-68
Lsamp	2.39E-69	-0.280	0.452	0.416	5.22E-65
Ptn	1.73E-65	-0.317	0.72	0.697	3.79E-61
Hist1h1b	6.60E-37	0.375	0.114	0.154	1.44E-32
AC154782.	2.61E-32	-0.268	0.14	0.134	5.70E-28
Nrxn3	2.96E-21	-0.262	0.371	0.369	6.47E-17

Gene	FC Het vs. WT	FC KO vs. WT	FC Ratio
Ttr	-1.892	-1.916	1.013
Malat1	-0.342	-0.404	1.183
Meg3	-0.264	-0.316	1.198
AC149090.	0.446	0.542	1.216
AC154782.	-0.268	-0.341	1.271
Gria2	-0.360	-0.480	1.332
Nrxn3	-0.262	-0.352	1.343
Marcks	0.269	0.370	1.378
Hnrnpa0	0.430	0.597	1.388
Actb	0.326	0.464	1.426
Chchd2	0.284	0.412	1.452
Fos	0.465	0.676	1.453
Ptprk	-0.323	-0.476	1.473
H2afv	0.256	0.383	1.498
Hist1h1e	0.687	1.040	1.513
Frmd4a	-0.311	-0.484	1.559
Cbx3	0.328	0.515	1.570
Ntrk2	-0.255	-0.403	1.579
Slc24a5	0.317	0.511	1.612
Gm10076	-0.467	-0.756	1.618
Rps29	-0.356	-0.583	1.639
Hist1h1b	0.375	0.618	1.647
Nrxn1	-0.287	-0.478	1.666
Uba52	-0.436	-0.731	1.676
Rps28	-0.353	-0.597	1.688
Atp5k	-0.277	-0.469	1.690
Itpr1	-0.263	-0.449	1.707
Gsdme	-0.277	-0.476	1.719
Hmgb2	0.336	0.580	1.727
H3f3b	0.375	0.654	1.745
Arpp21	-0.372	-0.654	1.759
Jund	0.320	0.563	1.761
Lsamp	-0.280	-0.512	1.828
Rpl38	-0.379	-0.692	1.829
Hba-a2	-1.211	-2.264	1.869
Hba-a1	-1.298	-2.429	1.872
Mef2c	-0.408	-0.769	1.884
Mdk	0.358	0.676	1.887
Ptn	-0.317	-0.602	1.897
Ptprd	-0.300	-0.575	1.914
Hbb-bs	-1.279	-2.494	1.949
Cdk2ap1	0.287	0.563	1.959
Mapt	-0.315	-0.625	1.982
Hbb-bt	-1.151	-2.305	2.002
Nfia	0.409	0.829	2.029
H1f0	0.264	0.536	2.029

Gatad2b	-0.305	-0.621	2.038
Grin2b	-0.343	-0.714	2.085

Gene	analysis	p_val	avg_log2FC	pct.1	pct.2	p_val_adj	cluster	chromosom	start_positi	end_position
Ptprk	het vs wt	2.18E-53	-0.495	0.703	0.798	4.76E-49	0	10	27950816	28473393
Rps28	het vs wt	5.86E-08	-0.455	0.997	0.999	0.001281	0	17	34038001	34043536
Rps29	het vs wt	1.78E-23	-0.426	0.999	0.999	3.88E-19	0	12	69204496	69205960
Zbtb20	het vs wt	2.81E-20	-0.381	0.344	0.471	6.14E-16	0	16	42696244	43462965
Gatad2b	het vs wt	2.06E-55	-0.348	0.808	0.885	4.49E-51	0	3	90200485	90270714
Ptn	het vs wt	5.28E-16	-0.337	0.993	0.995	1.15E-11	0	6	36691864	36787155
Itpr1	het vs wt	8.30E-26	-0.319	0.838	0.888	1.81E-21	0	6	1.08E+08	1.09E+08
Frmd4a	het vs wt	2.36E-22	-0.316	0.984	0.992	5.15E-18	0	2	4022528	4618854
Mef2c	het vs wt	1.16E-11	-0.315	0.991	0.99	2.53E-07	0	13	83652153	83815199
Lmo4	het vs wt	1.69E-50	-0.310	0.843	0.845	3.70E-46	0	3	1.44E+08	1.44E+08
Arpp21	het vs wt	2.21E-15	-0.300	0.895	0.92	4.82E-11	0	9	1.12E+08	1.12E+08
Atp5k	het vs wt	4.23E-12	-0.291	0.982	0.98	9.23E-08	0	5	1.09E+08	1.09E+08
Cntn2	het vs wt	1.77E-14	-0.275	0.902	0.917	3.87E-10	0	1	1.32E+08	1.32E+08
Cpe	het vs wt	3.98E-33	-0.265	0.694	0.814	8.69E-29	0	8	65045576	65146088
Grin2b	het vs wt	6.97E-07	-0.260	0.893	0.928	0.015218	0	6	1.36E+08	1.36E+08
Opcml	het vs wt	6.44E-10	-0.257	0.753	0.784	1.41E-05	0	9	27702071	28836706
Kif26b	het vs wt	4.62E-24	-0.255	0.406	0.519	1.01E-19	0	1	1.78E+08	1.79E+08
CT025619.	het vs wt	3.09E-18	-0.255	0.843	0.876	6.74E-14	0			
Ifitm2	het vs wt	6.29E-10	0.257	0.475	0.345	1.37E-05	0	7	1.41E+08	1.41E+08
Tmsb4x	het vs wt	2.93E-22	0.262	1	1	6.41E-18	0	X	1.66E+08	1.66E+08
Robo1	het vs wt	8.02E-40	0.274	0.865	0.735	1.75E-35	0	16	72105194	72842983
Sox5	het vs wt	7.86E-79	0.280	0.744	0.607	1.72E-74	0	6	1.44E+08	1.45E+08
Nfib	het vs wt	1.16E-59	0.294	0.992	0.981	2.54E-55	0	4	82208410	82623987
Oma1	het vs wt	8.58E-62	0.294	0.552	0.337	1.87E-57	0	4	1.03E+08	1.03E+08
Actb	het vs wt	1.64E-06	0.300	1	1	0.035875	0	5	1.43E+08	1.43E+08
H3f3b	het vs wt	2.82E-40	0.307	1	1	6.15E-36	0	11	1.16E+08	1.16E+08
Nrip1	het vs wt	1.79E-33	0.331	0.823	0.744	3.90E-29	0	16	76084288	76170715
Hist1h1e	het vs wt	6.97E-10	0.371	0.712	0.519	1.52E-05	0			
Hnrnpa0	het vs wt	4.56E-21	0.372	0.999	0.995	9.96E-17	0	13	58273693	58276370
Gm17750	het vs wt	5.43E-34	0.410	0.934	0.849	1.19E-29	0	13	84173416	84212922
Nrxn1	het vs wt	9.29E-09	-0.416	0.506	0.619	0.000203	1	17	90341059	91400499
Malat1	het vs wt	2.71E-12	-0.403	0.996	1	5.93E-08	1	19	5845717	5852704
Ttr	het vs wt	2.25E-46	-0.369	0	0.141	4.92E-42	1	18	20798337	20807378
Chl1	het vs wt	2.02E-09	-0.324	0.731	0.794	4.40E-05	1	6	1.03E+08	1.04E+08
Neurod6	het vs wt	1.73E-07	-0.317	0.229	0.35	0.003771	1	6	55654807	55658248
Celf4	het vs wt	1.78E-10	-0.312	0.949	0.961	3.89E-06	1	18	25610689	25887214
Mef2c	het vs wt	5.65E-08	-0.311	0.916	0.937	0.001233	1	13	83652153	83815199
Gsdme	het vs wt	2.41E-13	-0.294	0.509	0.573	5.27E-09	1	6	50165868	50240842
Gria1	het vs wt	4.67E-11	-0.288	0.757	0.815	1.02E-06	1	11	56902213	57221070
Gatad2b	het vs wt	3.25E-14	-0.277	0.498	0.587	7.10E-10	1	3	90200485	90270714
Dlgap1	het vs wt	3.75E-07	-0.275	0.814	0.862	0.008186	1	17	70276068	71128408
Ahi1	het vs wt	1.66E-13	-0.268	0.674	0.735	3.63E-09	1	10	20828446	20956328
Ckb	het vs wt	1.04E-06	0.259	0.865	0.777	0.022826	1	12	1.12E+08	1.12E+08
Cdkn1c	het vs wt	8.77E-08	0.260	0.548	0.481	0.001917	1	7	1.43E+08	1.43E+08
H3f3a	het vs wt	1.15E-12	0.324	0.997	0.994	2.51E-08	1	1	1.81E+08	1.81E+08
H3f3b	het vs wt	1.51E-15	0.381	0.999	0.998	3.31E-11	1	11	1.16E+08	1.16E+08
Actb	het vs wt	6.25E-08	0.385	0.999	0.998	0.001365	1	5	1.43E+08	1.43E+08
Nts	het vs wt	9.35E-19	-1.111	0.054	0.137	2.04E-14	2	10	1.02E+08	1.02E+08
Rps29	het vs wt	2.33E-08	-0.397	0.999	0.998	0.000509	2	12	69204496	69205960
Atp5k	het vs wt	1.78E-19	-0.331	0.959	0.942	3.88E-15	2	5	1.09E+08	1.09E+08
Gatad2b	het vs wt	1.48E-31	-0.331	0.744	0.808	3.23E-27	2	3	90200485	90270714
Ntm	het vs wt	1.80E-10	-0.313	0.561	0.595	3.94E-06	2	9	28906046	29874437
Syt4	het vs wt	9.77E-07	-0.301	0.553	0.578	0.021339	2	18	31570861	31580459
Pcsk2	het vs wt	2.27E-18	-0.280	0.348	0.432	4.96E-14	2	2	1.43E+08	1.44E+08
Etv1	het vs wt	4.55E-14	-0.278	0.185	0.272	9.93E-10	2	12	38829379	38920483
Scd2	het vs wt	5.04E-10	-0.278	0.908	0.923	1.10E-05	2	19	44282113	44295303

Lmo4	het vs wt	1.15E-14	-0.275	0.717	0.726	2.50E-10	2	3	1.44E+08	1.44E+08
Ntrk2	het vs wt	3.44E-11	-0.272	0.803	0.806	7.52E-07	2	13	58954383	59281784
Grin2b	het vs wt	1.30E-06	-0.269	0.984	0.974	0.028319	2	6	1.36E+08	1.36E+08
Nrp1	het vs wt	1.01E-07	-0.265	0.395	0.438	0.002199	2	8	1.29E+08	1.29E+08
Meg3	het vs wt	9.23E-08	-0.264	0.958	0.911	0.002016	2	12	1.1E+08	1.1E+08
Ttr	het vs wt	3.28E-69	-0.261	0	0.187	7.16E-65	2	18	20798337	20807378
Sparcl1	het vs wt	4.88E-14	-0.257	0.718	0.711	1.07E-09	2	5	1.04E+08	1.04E+08
Pcdh17	het vs wt	5.66E-09	-0.256	0.304	0.36	0.000124	2	14	84681003	84776442
Dpy19l1	het vs wt	4.50E-28	0.259	0.739	0.595	9.82E-24	2	9	24323072	24414436
Jund	het vs wt	9.61E-17	0.295	0.948	0.878	2.10E-12	2	8	71151599	71153265
Jun	het vs wt	5.34E-14	0.302	0.524	0.352	1.17E-09	2	4	94937271	94940459
H3f3b	het vs wt	5.20E-31	0.327	0.998	0.992	1.14E-26	2	11	1.16E+08	1.16E+08
Hnrnpa0	het vs wt	5.06E-09	0.368	0.994	0.967	0.000111	2	13	58273693	58276370
Nfia	het vs wt	1.41E-16	0.446	0.946	0.878	3.08E-12	2	4	97660971	98007111
Gria2	het vs wt	2.71E-14	-0.506	0.997	0.998	5.92E-10	3	3	80588757	80710142
Rps29	het vs wt	6.55E-12	-0.367	0.999	1	1.43E-07	3	12	69204496	69205960
Mapt	het vs wt	7.37E-23	-0.364	0.898	0.944	1.61E-18	3	11	1.04E+08	1.04E+08
Rsrp1	het vs wt	5.06E-12	-0.363	0.949	0.975	1.10E-07	3	4	1.35E+08	1.35E+08
Gatad2b	het vs wt	1.24E-21	-0.361	0.721	0.837	2.70E-17	3	3	90200485	90270714
CT025619.:	het vs wt	2.15E-20	-0.346	0.866	0.92	4.70E-16	3			
Pou3f2	het vs wt	1.03E-09	-0.344	0.951	0.966	2.25E-05	3	4	22482780	22488366
Rbfox1	het vs wt	3.56E-19	-0.328	0.746	0.816	7.79E-15	3	16	5703219	7229390
Shtn1	het vs wt	1.74E-10	-0.319	0.773	0.842	3.81E-06	3	19	58961788	59064532
Ptn	het vs wt	4.78E-13	-0.317	0.942	0.953	1.04E-08	3	6	36691864	36787155
Lsamp	het vs wt	7.71E-13	-0.311	0.643	0.712	1.68E-08	3	16	39804723	42002042
Arpp21	het vs wt	1.69E-17	-0.290	0.424	0.532	3.69E-13	3	9	1.12E+08	1.12E+08
Jph4	het vs wt	9.10E-07	-0.284	0.866	0.892	0.01989	3	14	55344283	55354392
Hist1h2ap	het vs wt	3.80E-07	-0.281	0.536	0.609	0.008306	3			
Ttr	het vs wt	1.42E-52	-0.280	0	0.182	3.10E-48	3	18	20798337	20807378
Fam171b	het vs wt	1.82E-12	-0.277	0.715	0.792	3.98E-08	3	2	83642980	83713830
Tsc22d1	het vs wt	1.27E-06	-0.269	0.996	0.999	0.027834	3	14	76652401	76745205
Hivep3	het vs wt	1.68E-10	-0.263	0.522	0.626	3.66E-06	3	4	1.2E+08	1.2E+08
D430041D(	het vs wt	2.70E-19	-0.257	0.431	0.53	5.90E-15	3	2	1.04E+08	1.04E+08
Itpr1	het vs wt	1.29E-11	-0.255	0.412	0.498	2.82E-07	3	6	1.08E+08	1.09E+08
Chchd2	het vs wt	5.68E-08	0.281	0.999	0.995	0.00124	3	5	1.3E+08	1.3E+08
Rprm	het vs wt	2.09E-07	0.297	0.362	0.253	0.004561	3	2	53974105	53975564
Fabp7	het vs wt	6.77E-07	0.309	0.734	0.628	0.014797	3	10	57660977	57664546
Vim	het vs wt	9.97E-16	0.340	0.427	0.274	2.18E-11	3	2	13578738	13587637
Bcl11b	het vs wt	3.65E-23	0.344	0.764	0.581	7.98E-19	3	12	1.08E+08	1.08E+08
AC149090.	het vs wt	3.51E-08	0.371	0.63	0.389	0.000767	3			
Nfia	het vs wt	2.96E-20	0.524	0.929	0.849	6.46E-16	3	4	97660971	98007111
Rps28	het vs wt	9.86E-07	-0.450	0.994	0.997	0.021532	4	17	34038001	34043536
Rps29	het vs wt	8.89E-14	-0.439	0.999	0.999	1.94E-09	4	12	69204496	69205960
Gatad2b	het vs wt	3.03E-18	-0.323	0.662	0.76	6.62E-14	4	3	90200485	90270714
Gria2	het vs wt	5.97E-09	-0.322	0.993	0.988	0.00013	4	3	80588757	80710142
Frmd4a	het vs wt	1.40E-12	-0.292	0.895	0.928	3.05E-08	4	2	4022528	4618854
Eif1b	het vs wt	2.24E-11	-0.290	0.988	0.982	4.88E-07	4	9	1.2E+08	1.2E+08
Thsd7a	het vs wt	2.46E-10	-0.271	0.427	0.493	5.38E-06	4	6	12311609	12749409
Nudcd3	het vs wt	5.23E-11	-0.270	0.794	0.839	1.14E-06	4	11	6055691	6150415
Ttr	het vs wt	1.64E-41	-0.259	0.001	0.157	3.59E-37	4	18	20798337	20807378
Nr2f2	het vs wt	5.31E-08	0.291	0.718	0.638	0.001161	4	7	70001692	70016483
Chchd2	het vs wt	7.83E-07	0.307	0.994	0.983	0.017107	4	5	1.3E+08	1.3E+08
Nfia	het vs wt	1.36E-10	0.372	0.577	0.473	2.97E-06	4	4	97660971	98007111
Nefm	het vs wt	2.84E-08	0.416	0.442	0.355	0.000621	4	14	68320039	68362295
AC149090.	het vs wt	6.21E-08	0.488	0.702	0.435	0.001356	4			
Ttr	het vs wt	8.78E-27	-4.600	0.001	0.14	1.92E-22	5	18	20798337	20807378
Mapt	het vs wt	5.39E-07	-0.690	0.431	0.541	0.011772	5	11	1.04E+08	1.04E+08

Mef2c	het vs wt	1.36E-06	-0.645	0.274	0.357	0.029689	5	13	83652153	83815199
Tubb2a	het vs wt	5.40E-07	-0.473	0.435	0.478	0.011795	5	13	34258257	34261990
Ccnd2	het vs wt	4.43E-07	-0.319	0.353	0.257	0.009685	5	6	1.27E+08	1.27E+08
Tmsb10	het vs wt	4.04E-07	-0.296	0.957	0.985	0.008819	5	6	72934330	72935731
Tuba1a	het vs wt	5.09E-15	-0.280	0.969	0.993	1.11E-10	5	15	98847718	98851584
Mir9-3hg	het vs wt	2.03E-06	0.252	0.139	0.088	0.044283	5	7	79148856	79184151
Ezh2	het vs wt	1.84E-07	0.344	0.385	0.235	0.004025	5	6	47507073	47572275
Ccar1	het vs wt	2.84E-07	0.353	0.252	0.176	0.006197	5	10	62579707	62628065
Golga4	het vs wt	7.66E-07	0.377	0.138	0.081	0.016734	5	9	1.18E+08	1.18E+08
Phip	het vs wt	1.78E-08	0.455	0.322	0.209	0.000388	5	9	82748212	82857569
Meis2	het vs wt	1.68E-10	0.476	0.575	0.449	3.67E-06	5	2	1.16E+08	1.16E+08
Mki67	het vs wt	1.06E-07	0.557	0.144	0.073	0.002308	5	7	1.35E+08	1.35E+08
Pax6	het vs wt	2.39E-09	0.596	0.141	0.065	5.23E-05	5	2	1.05E+08	1.06E+08
Hmgn2	het vs wt	6.54E-07	0.648	0.538	0.389	0.014278	5	4	1.34E+08	1.34E+08
Prc1	het vs wt	5.70E-09	0.660	0.144	0.07	0.000125	5	7	79944198	79966007
Top2a	het vs wt	3.15E-07	0.691	0.213	0.119	0.006877	5	11	98883769	98915015
Mef2c	het vs wt	5.80E-10	-0.525	0.514	0.665	1.27E-05	6	13	83652153	83815199
Mapt	het vs wt	1.83E-06	-0.480	0.797	0.847	0.040069	6	11	1.04E+08	1.04E+08
Ttr	het vs wt	1.42E-14	-0.438	0.001	0.123	3.10E-10	6	18	20798337	20807378
Pcsk1n	het vs wt	7.31E-07	-0.263	0.44	0.514	0.015966	6	X	7786061	7790649
Tpt1	het vs wt	1.25E-10	0.299	0.987	0.99	2.73E-06	6	14	76082533	76085965
Zfhx4	het vs wt	1.99E-06	0.326	0.623	0.477	0.04342	6	3	5283586	5480917
Ezr	het vs wt	1.01E-07	0.403	0.281	0.178	0.002214	6	17	7005440	7050183
Adarb2	het vs wt	1.99E-06	-0.565	0.848	0.867	0.043421	7	13	8252902	8818783
Malat1	het vs wt	1.82E-08	-0.521	0.995	1	0.000398	7	19	5845717	5852704
Ttr	het vs wt	3.21E-17	-0.436	0	0.123	7.02E-13	7	18	20798337	20807378
Gm3764	het vs wt	1.80E-08	-0.420	0.824	0.83	0.000393	7	3	88113837	88137268
Dlgap1	het vs wt	2.50E-07	-0.415	0.255	0.341	0.005467	7	17	70276068	71128408
Uba52	het vs wt	8.84E-08	-0.401	0.789	0.782	0.001931	7	8	70960913	70963451
Nbea	het vs wt	1.11E-10	-0.332	0.849	0.805	2.42E-06	7	3	55532616	56091122
Myt1l	het vs wt	8.06E-07	-0.330	0.877	0.886	0.017601	7	12	29578383	29973212
Git2	het vs wt	6.11E-07	-0.278	0.35	0.338	0.01336	7	5	1.15E+08	1.15E+08
Pafah1b3	het vs wt	5.50E-09	0.257	0.905	0.786	0.00012	7	7	24994474	24997411
H1fx	het vs wt	9.14E-07	0.258	0.785	0.651	0.019974	7			
Ftl1	het vs wt	4.10E-12	0.267	0.986	0.964	8.96E-08	7	7	45107368	45109308
Selenow	het vs wt	1.22E-06	0.269	0.964	0.873	0.026575	7	7	15651133	15656327
Tpt1	het vs wt	2.60E-07	0.270	0.995	0.979	0.005676	7	14	76082533	76085965
Nnat	het vs wt	3.09E-08	0.273	0.993	0.961	0.000675	7	2	1.57E+08	1.57E+08
Atp5j	het vs wt	9.76E-10	0.278	0.906	0.787	2.13E-05	7	16	84624754	84632513
Eef1b2	het vs wt	3.75E-07	0.297	0.915	0.821	0.008191	7	1	63215984	63219645
Hist3h2ba	het vs wt	9.86E-07	0.320	0.88	0.743	0.021549	7			
H3f3b	het vs wt	2.40E-08	0.430	0.999	0.975	0.000525	7	11	1.16E+08	1.16E+08
Ptprz1	het vs wt	2.81E-07	-0.597	0.909	0.888	0.006142	8	6	22875501	23052915
Cspg5	het vs wt	4.68E-08	-0.575	0.441	0.5	0.001023	8	9	1.1E+08	1.1E+08
Luzp2	het vs wt	6.55E-07	-0.476	0.196	0.303	0.014318	8	7	54485246	54918633
Serpine2	het vs wt	1.63E-06	-0.454	0.121	0.178	0.035512	8	1	79771914	79838897
Bcan	het vs wt	1.23E-08	-0.378	0.179	0.274	0.000269	8	3	87894838	87907537
Olig1	het vs wt	5.54E-10	-0.376	0.104	0.202	1.21E-05	8	16	91066660	91068821
Gm3764	het vs wt	1.54E-06	-0.331	0.721	0.77	0.033683	8	3	88113837	88137268
Atp5k	het vs wt	2.00E-07	-0.309	0.972	0.969	0.004359	8	5	1.09E+08	1.09E+08
Fam181b	het vs wt	6.82E-07	-0.306	0.424	0.479	0.01489	8	7	92729073	92731074
Tubb2a	het vs wt	1.13E-09	-0.306	0.7	0.782	2.47E-05	8	13	34258257	34261990
Rlbp1	het vs wt	4.51E-07	-0.304	0.364	0.436	0.009856	8	7	79024618	79036796
Xylt1	het vs wt	5.69E-09	-0.299	0.168	0.262	0.000124	8	7	1.17E+08	1.17E+08
Rtn1	het vs wt	4.38E-10	-0.269	0.961	0.968	9.57E-06	8	12	72258526	72455828
Lrp1	het vs wt	1.66E-06	-0.266	0.447	0.525	0.036289	8	10	1.27E+08	1.27E+08
Ttr	het vs wt	8.43E-29	-0.266	0.005	0.176	1.84E-24	8	18	20798337	20807378

Olig2	het vs wt	3.95E-08	-0.266	0.307	0.341	0.000862	8	16	91022345	91025565
Gatad2b	het vs wt	4.51E-09	-0.258	0.747	0.803	9.84E-05	8	3	90200485	90270714
Insm1	het vs wt	4.81E-07	0.278	0.754	0.633	0.010507	8	2	1.46E+08	1.46E+08
Mdk	het vs wt	4.04E-19	0.459	0.971	0.901	8.82E-15	8	2	91760150	91762642
Ttr	het vs wt	1.74E-17	-0.397	0	0.126	3.80E-13	9	18	20798337	20807378
Actg1	het vs wt	1.71E-08	0.282	0.986	0.957	0.000373	9	11	1.2E+08	1.2E+08
H1f0	het vs wt	5.91E-08	0.382	0.628	0.429	0.001292	9	15	78912650	78914704
H3f3b	het vs wt	6.15E-08	0.486	0.985	0.969	0.001345	9	11	1.16E+08	1.16E+08
Negr1	het vs wt	1.01E-07	-0.393	0.61	0.703	0.002202	10	3	1.56E+08	1.57E+08
Rps29	het vs wt	6.07E-10	-0.389	0.997	1	1.33E-05	10	12	69204496	69205960
Gm17750	het vs wt	4.32E-09	-0.335	0.705	0.779	9.45E-05	10	13	84173416	84212922
Pou3f2	het vs wt	9.25E-09	-0.283	0.961	0.951	0.000202	10	4	22482780	22488366
Igfbp2	het vs wt	6.00E-12	0.326	0.787	0.676	1.31E-07	10	1	72863662	72891633
Ckb	het vs wt	1.15E-06	0.397	0.934	0.903	0.025083	10	12	1.12E+08	1.12E+08
Rps28	het vs wt	1.13E-12	-0.468	0.999	0.999	2.47E-08	11	17	34038001	34043536
Rpl38	het vs wt	6.56E-08	-0.422	0.999	1	0.001434	11	11	1.15E+08	1.15E+08
Rps29	het vs wt	5.12E-14	-0.419	0.999	1	1.12E-09	11	12	69204496	69205960
Pantr1	het vs wt	4.71E-11	-0.402	0.786	0.815	1.03E-06	11	1	42629754	42734584
Gatad2b	het vs wt	4.11E-14	-0.340	0.834	0.909	8.98E-10	11	3	90200485	90270714
Plxna4	het vs wt	1.38E-09	-0.324	0.418	0.54	3.02E-05	11	6	32121203	32565127
Tsc22d1	het vs wt	9.20E-08	-0.297	0.994	0.988	0.002011	11	14	76652401	76745205
Frmd4a	het vs wt	1.16E-07	-0.285	0.984	0.979	0.002526	11	2	4022528	4618854
Rsrp1	het vs wt	2.80E-07	-0.280	0.985	0.981	0.006107	11	4	1.35E+08	1.35E+08
Ckb	het vs wt	5.74E-12	0.256	0.995	0.982	1.26E-07	11	12	1.12E+08	1.12E+08
Edil3	het vs wt	5.25E-12	0.290	0.95	0.853	1.15E-07	11	13	88969591	89471342
Bcl11b	het vs wt	8.89E-07	0.312	0.892	0.832	0.01942	11	12	1.08E+08	1.08E+08
Vim	het vs wt	6.87E-11	0.344	0.779	0.652	1.50E-06	11	2	13578738	13587637
Ifitm2	het vs wt	2.28E-08	0.439	0.785	0.609	0.000497	11	7	1.41E+08	1.41E+08
Sox5	het vs wt	7.24E-15	0.456	0.707	0.561	1.58E-10	11	6	1.44E+08	1.45E+08
Ttr	het vs wt	1.89E-14	-0.312	0	0.126	4.12E-10	12	18	20798337	20807378
Mau2	het vs wt	1.21E-07	-0.310	0.467	0.495	0.002652	12	8	70468773	70495384
Gatad2b	het vs wt	4.22E-08	-0.306	0.575	0.627	0.000923	12	3	90200485	90270714
Cald1	het vs wt	2.91E-08	-0.289	0.567	0.6	0.000636	12	6	34575435	34752408
Tim+G169:	het vs wt	6.25E-08	0.250	0.807	0.639	0.001365	12	10	80735284	80736803
Hmgn1	het vs wt	1.37E-06	0.252	0.972	0.902	0.029892	12	16	95921818	95928929
Rps10	het vs wt	9.45E-07	0.258	0.994	0.962	0.02064	12	17	27849392	27855643
Ndufab1	het vs wt	4.27E-07	0.261	0.822	0.672	0.009334	12	7	1.22E+08	1.22E+08
Mif	het vs wt	1.23E-06	0.277	0.896	0.802	0.026771	12	10	75695187	75696074
Eef1b2	het vs wt	2.39E-08	0.284	0.95	0.881	0.000523	12	1	63215984	63219645
Tpt1	het vs wt	1.05E-07	0.317	1	0.997	0.002293	12	14	76082533	76085965
Hmgn2	het vs wt	1.61E-07	0.341	0.928	0.818	0.003524	12	4	1.34E+08	1.34E+08
Zic1	het vs wt	6.33E-11	0.409	0.192	0.11	1.38E-06	12	9	91240111	91247863
Mdk	het vs wt	4.96E-07	0.530	0.519	0.355	0.010828	12	2	91760150	91762642
Ttr	het vs wt	1.94E-17	-1.384	0	0.136	4.24E-13	13	18	20798337	20807378
Rps29	het vs wt	1.11E-14	-0.692	0.688	0.88	2.43E-10	13	12	69204496	69205960
Rps28	het vs wt	2.56E-08	-0.634	0.541	0.757	0.00056	13	17	34038001	34043536
Rpl38	het vs wt	1.55E-08	-0.585	0.569	0.815	0.000339	13	11	1.15E+08	1.15E+08
Cd24a	het vs wt	1.26E-06	-0.575	0.187	0.346	0.027475	13	10	43454280	43460261
Rps21	het vs wt	4.13E-08	-0.527	0.527	0.736	0.000902	13	2	1.8E+08	1.8E+08
Rpl6	het vs wt	2.24E-06	-0.521	0.532	0.703	0.049044	13	5	1.21E+08	1.21E+08
Rpl37	het vs wt	1.49E-07	-0.521	0.614	0.781	0.003254	13	15	5146127	5148622
Tmsb10	het vs wt	3.68E-13	-0.494	0.871	0.966	8.03E-09	13	6	72934330	72935731
Rpl39	het vs wt	1.40E-06	-0.493	0.499	0.671	0.03066	13	X	36346173	36349055
Snrpd1	het vs wt	1.61E-07	-0.455	0.109	0.217	0.003509	13	18	10617775	10642079
Eef1a1	het vs wt	9.62E-11	-0.448	0.796	0.92	2.10E-06	13	9	78385731	78396433
Rps10	het vs wt	3.48E-08	-0.429	0.473	0.669	0.00076	13	17	27849392	27855643
Rpl23	het vs wt	9.26E-09	-0.418	0.627	0.772	0.000202	13	11	97668353	97673263

Rps16	het vs wt	3.51E-11	-0.414	0.463	0.616	7.67E-07	13	7	28050077	28052580
Rpl30	het vs wt	1.24E-07	-0.406	0.524	0.677	0.002707	13	15	34440651	34443786
Rpl7	het vs wt	8.49E-07	-0.405	0.441	0.601	0.018549	13	1	16171519	16174886
Fau	het vs wt	3.19E-07	-0.402	0.529	0.69	0.006959	13	19	6107874	6109554
Tubb2b	het vs wt	1.12E-09	-0.390	0.751	0.877	2.44E-05	13	13	34310731	34314449
Rps27a	het vs wt	1.83E-08	-0.379	0.579	0.741	0.0004	13	11	29495846	29498109
Rpsa	het vs wt	8.07E-10	-0.377	0.58	0.759	1.76E-05	13	9	1.2E+08	1.2E+08
Stmn1	het vs wt	1.13E-14	-0.355	0.854	0.959	2.48E-10	13	4	1.34E+08	1.34E+08
Rpl34	het vs wt	5.34E-08	-0.352	0.509	0.683	0.001167	13	3	1.31E+08	1.31E+08
Rpl21	het vs wt	3.18E-09	-0.349	0.516	0.677	6.94E-05	13	5	1.47E+08	1.47E+08
Stmn3	het vs wt	1.03E-07	-0.344	0.313	0.43	0.002261	13	2	1.81E+08	1.81E+08
Rps7	het vs wt	6.19E-09	-0.313	0.584	0.73	0.000135	13	12	28680853	28685952
Rpl24	het vs wt	6.76E-07	-0.309	0.463	0.625	0.01477	13	16	55786638	55791798
Rpl32	het vs wt	1.31E-08	-0.308	0.567	0.724	0.000287	13	6	1.16E+08	1.16E+08
Rps14	het vs wt	1.52E-06	-0.307	0.546	0.693	0.033214	13	18	60880170	60911618
Tuba1a	het vs wt	1.88E-20	-0.301	0.909	0.97	4.10E-16	13	15	98847718	98851584
Rpl13	het vs wt	3.97E-09	-0.296	0.692	0.81	8.67E-05	13	8	1.24E+08	1.24E+08
Rps5	het vs wt	1.24E-07	-0.289	0.517	0.673	0.002707	13	7	12656217	12660613
Rps8	het vs wt	6.61E-08	-0.263	0.718	0.831	0.001444	13	4	1.17E+08	1.17E+08
Tuba1a	het vs wt	1.79E-07	-0.395	0.93	0.969	0.003906	15	15	98847718	98851584
Tmsb4x	het vs wt	3.19E-07	-0.338	0.965	0.978	0.006959	15	X	1.66E+08	1.66E+08
Srrm2	het vs wt	1.42E-06	0.337	0.733	0.598	0.030976	15	17	24009506	24043715
Malat1	het vs wt	3.60E-07	0.467	1	1	0.007863	15	19	5845717	5852704
Gatad2b	het vs wt	1.23E-06	-0.311	0.817	0.885	0.026837	16	3	90200485	90270714

Gene	p_val	avg_log2F	pct.1	pct.2	p_val_adj	cluster	analysis	chromosom	start_posit	end_positi	Number of clusters
Ttr	2.25E-46	-0.369	0	0.141	4.92E-42	1	het vs wt	18	20798337	20807378	11
Ttr	1.89E-14	-0.312	0	0.126	4.12E-10	12	het vs wt	18	20798337	20807378	11
Ttr	1.94E-17	-1.384	0	0.136	4.24E-13	13	het vs wt	18	20798337	20807378	11
Ttr	3.28E-69	-0.261	0	0.187	7.16E-65	2	het vs wt	18	20798337	20807378	11
Ttr	1.42E-52	-0.280	0	0.182	3.10E-48	3	het vs wt	18	20798337	20807378	11
Ttr	1.64E-41	-0.259	0.001	0.157	3.59E-37	4	het vs wt	18	20798337	20807378	11
Ttr	8.78E-27	-4.600	0.001	0.14	1.92E-22	5	het vs wt	18	20798337	20807378	11
Ttr	1.42E-14	-0.438	0.001	0.123	3.10E-10	6	het vs wt	18	20798337	20807378	11
Ttr	3.21E-17	-0.436	0	0.123	7.02E-13	7	het vs wt	18	20798337	20807378	11
Ttr	8.43E-29	-0.266	0.005	0.176	1.84E-24	8	het vs wt	18	20798337	20807378	11
Ttr	1.74E-17	-0.397	0	0.126	3.80E-13	9	het vs wt	18	20798337	20807378	11
Gatad2b	2.06E-55	-0.348	0.808	0.885	4.49E-51	0	het vs wt	3	90200485	90270714	9
Gatad2b	3.25E-14	-0.277	0.498	0.587	7.10E-10	1	het vs wt	3	90200485	90270714	9
Gatad2b	4.11E-14	-0.340	0.834	0.909	8.98E-10	11	het vs wt	3	90200485	90270714	9
Gatad2b	4.22E-08	-0.306	0.575	0.627	0.000923	12	het vs wt	3	90200485	90270714	9
Gatad2b	1.23E-06	-0.311	0.817	0.885	0.026837	16	het vs wt	3	90200485	90270714	9
Gatad2b	1.48E-31	-0.331	0.744	0.808	3.23E-27	2	het vs wt	3	90200485	90270714	9
Gatad2b	1.24E-21	-0.361	0.721	0.837	2.70E-17	3	het vs wt	3	90200485	90270714	9
Gatad2b	3.03E-18	-0.323	0.662	0.76	6.62E-14	4	het vs wt	3	90200485	90270714	9
Gatad2b	4.51E-09	-0.258	0.747	0.803	9.84E-05	8	het vs wt	3	90200485	90270714	9
Rps29	1.78E-23	-0.426	0.999	0.999	3.88E-19	0	het vs wt	12	69204496	69205960	7
Rps29	6.07E-10	-0.389	0.997	1	1.33E-05	10	het vs wt	12	69204496	69205960	7
Rps29	5.12E-14	-0.419	0.999	1	1.12E-09	11	het vs wt	12	69204496	69205960	7
Rps29	1.11E-14	-0.692	0.688	0.88	2.43E-10	13	het vs wt	12	69204496	69205960	7
Rps29	2.33E-08	-0.397	0.999	0.998	0.000509	2	het vs wt	12	69204496	69205960	7
Rps29	6.55E-12	-0.367	0.999	1	1.43E-07	3	het vs wt	12	69204496	69205960	7
Rps29	8.89E-14	-0.439	0.999	0.999	1.94E-09	4	het vs wt	12	69204496	69205960	7
H3f3b	2.82E-40	0.307	1	1	6.15E-36	0	het vs wt	11	1.16E+08	1.16E+08	5
H3f3b	1.51E-15	0.381	0.999	0.998	3.31E-11	1	het vs wt	11	1.16E+08	1.16E+08	5
H3f3b	5.20E-31	0.327	0.998	0.992	1.14E-26	2	het vs wt	11	1.16E+08	1.16E+08	5
H3f3b	2.40E-08	0.430	0.999	0.975	0.000525	7	het vs wt	11	1.16E+08	1.16E+08	5
H3f3b	6.15E-08	0.486	0.985	0.969	0.001345	9	het vs wt	11	1.16E+08	1.16E+08	5
Mef2c	1.16E-11	-0.315	0.991	0.99	2.53E-07	0	het vs wt	13	83652153	83815199	4
Mef2c	5.65E-08	-0.311	0.916	0.937	0.001233	1	het vs wt	13	83652153	83815199	4
Mef2c	1.36E-06	-0.645	0.274	0.357	0.029689	5	het vs wt	13	83652153	83815199	4
Mef2c	5.80E-10	-0.525	0.514	0.665	1.27E-05	6	het vs wt	13	83652153	83815199	4
Rps28	5.86E-08	-0.455	0.997	0.999	0.001281	0	het vs wt	17	34038001	34043536	4
Rps28	1.13E-12	-0.468	0.999	0.999	2.47E-08	11	het vs wt	17	34038001	34043536	4
Rps28	2.56E-08	-0.634	0.541	0.757	0.00056	13	het vs wt	17	34038001	34043536	4
Rps28	9.86E-07	-0.450	0.994	0.997	0.021532	4	het vs wt	17	34038001	34043536	4
Atp5k	4.23E-12	-0.291	0.982	0.98	9.23E-08	0	het vs wt	5	1.09E+08	1.09E+08	3
Atp5k	1.78E-19	-0.331	0.959	0.942	3.88E-15	2	het vs wt	5	1.09E+08	1.09E+08	3
Atp5k	2.00E-07	-0.309	0.972	0.969	0.004359	8	het vs wt	5	1.09E+08	1.09E+08	3
Ckb	1.04E-06	0.259	0.865	0.777	0.022826	1	het vs wt	12	1.12E+08	1.12E+08	3
Ckb	1.15E-06	0.397	0.934	0.903	0.025083	10	het vs wt	12	1.12E+08	1.12E+08	3
Ckb	5.74E-12	0.256	0.995	0.982	1.26E-07	11	het vs wt	12	1.12E+08	1.12E+08	3
Frmd4a	2.36E-22	-0.316	0.984	0.992	5.15E-18	0	het vs wt	2	4022528	4618854	3
Frmd4a	1.16E-07	-0.285	0.984	0.979	0.002526	11	het vs wt	2	4022528	4618854	3
Frmd4a	1.40E-12	-0.292	0.895	0.928	3.05E-08	4	het vs wt	2	4022528	4618854	3
Malat1	2.71E-12	-0.403	0.996	1	5.93E-08	1	het vs wt	19	5845717	5852704	3
Malat1	3.60E-07	0.467	1	1	0.007863	15	het vs wt	19	5845717	5852704	3
Malat1	1.82E-08	-0.521	0.995	1	0.000398	7	het vs wt	19	5845717	5852704	3
Mapt	7.37E-23	-0.364	0.898	0.944	1.61E-18	3	het vs wt	11	1.04E+08	1.04E+08	3
Mapt	5.39E-07	-0.690	0.431	0.541	0.011772	5	het vs wt	11	1.04E+08	1.04E+08	3
Mapt	1.83E-06	-0.480	0.797	0.847	0.040069	6	het vs wt	11	1.04E+08	1.04E+08	3
Nfia	1.41E-16	0.446	0.946	0.878	3.08E-12	2	het vs wt	4	97660971	98007111	3
Nfia	2.96E-20	0.524	0.929	0.849	6.46E-16	3	het vs wt	4	97660971	98007111	3
Nfia	1.36E-10	0.372	0.577	0.473	2.97E-06	4	het vs wt	4	97660971	98007111	3
Tpt1	1.05E-07	0.317	1	0.997	0.002293	12	het vs wt	14	76082533	76085965	3
Tpt1	1.25E-10	0.299	0.987	0.99	2.73E-06	6	het vs wt	14	76082533	76085965	3

Tpt1	2.60E-07	0.270	0.995	0.979	0.005676	7	het vs wt	14	76082533	76085965	3
Tuba1a	1.88E-20	-0.301	0.909	0.97	4.10E-16	13	het vs wt	15	98847718	98851584	3
Tuba1a	1.79E-07	-0.395	0.93	0.969	0.003906	15	het vs wt	15	98847718	98851584	3
Tuba1a	5.09E-15	-0.280	0.969	0.993	1.11E-10	5	het vs wt	15	98847718	98851584	3
AC149090.	3.51E-08	0.371	0.63	0.389	0.000767	3	het vs wt				2
AC149090.	6.21E-08	0.488	0.702	0.435	0.001356	4	het vs wt				2
Actb	1.64E-06	0.300	1	1	0.035875	0	het vs wt	5	1.43E+08	1.43E+08	2
Actb	6.25E-08	0.385	0.999	0.998	0.001365	1	het vs wt	5	1.43E+08	1.43E+08	2
Arpp21	2.21E-15	-0.300	0.895	0.92	4.82E-11	0	het vs wt	9	1.12E+08	1.12E+08	2
Arpp21	1.69E-17	-0.290	0.424	0.532	3.69E-13	3	het vs wt	9	1.12E+08	1.12E+08	2
Bcl11b	8.89E-07	0.312	0.892	0.832	0.01942	11	het vs wt	12	1.08E+08	1.08E+08	2
Bcl11b	3.65E-23	0.344	0.764	0.581	7.98E-19	3	het vs wt	12	1.08E+08	1.08E+08	2
Chchd2	5.68E-08	0.281	0.999	0.995	0.00124	3	het vs wt	5	1.3E+08	1.3E+08	2
Chchd2	7.83E-07	0.307	0.994	0.983	0.017107	4	het vs wt	5	1.3E+08	1.3E+08	2
CT025619.:	3.09E-18	-0.255	0.843	0.876	6.74E-14	0	het vs wt				2
CT025619.:	2.15E-20	-0.346	0.866	0.92	4.70E-16	3	het vs wt				2
Dlgap1	3.75E-07	-0.275	0.814	0.862	0.008186	1	het vs wt	17	70276068	71128408	2
Dlgap1	2.50E-07	-0.415	0.255	0.341	0.005467	7	het vs wt	17	70276068	71128408	2
Eef1b2	2.39E-08	0.284	0.95	0.881	0.000523	12	het vs wt	1	63215984	63219645	2
Eef1b2	3.75E-07	0.297	0.915	0.821	0.008191	7	het vs wt	1	63215984	63219645	2
Gm17750	5.43E-34	0.410	0.934	0.849	1.19E-29	0	het vs wt	13	84173416	84212922	2
Gm17750	4.32E-09	-0.335	0.705	0.779	9.45E-05	10	het vs wt	13	84173416	84212922	2
Gm3764	1.80E-08	-0.420	0.824	0.83	0.000393	7	het vs wt	3	88113837	88137268	2
Gm3764	1.54E-06	-0.331	0.721	0.77	0.033683	8	het vs wt	3	88113837	88137268	2
Gria2	2.71E-14	-0.506	0.997	0.998	5.92E-10	3	het vs wt	3	80588757	80710142	2
Gria2	5.97E-09	-0.322	0.993	0.988	0.00013	4	het vs wt	3	80588757	80710142	2
Grin2b	6.97E-07	-0.260	0.893	0.928	0.015218	0	het vs wt	6	1.36E+08	1.36E+08	2
Grin2b	1.30E-06	-0.269	0.984	0.974	0.028319	2	het vs wt	6	1.36E+08	1.36E+08	2
Hmgn2	1.61E-07	0.341	0.928	0.818	0.003524	12	het vs wt	4	1.34E+08	1.34E+08	2
Hmgn2	6.54E-07	0.648	0.538	0.389	0.014278	5	het vs wt	4	1.34E+08	1.34E+08	2
Hnrnpa0	4.56E-21	0.372	0.999	0.995	9.96E-17	0	het vs wt	13	58273693	58276370	2
Hnrnpa0	5.06E-09	0.368	0.994	0.967	0.000111	2	het vs wt	13	58273693	58276370	2
Ifitm2	6.29E-10	0.257	0.475	0.345	1.37E-05	0	het vs wt	7	1.41E+08	1.41E+08	2
Ifitm2	2.28E-08	0.439	0.785	0.609	0.000497	11	het vs wt	7	1.41E+08	1.41E+08	2
Itpr1	8.30E-26	-0.319	0.838	0.888	1.81E-21	0	het vs wt	6	1.08E+08	1.09E+08	2
Itpr1	1.29E-11	-0.255	0.412	0.498	2.82E-07	3	het vs wt	6	1.08E+08	1.09E+08	2
Lmo4	1.69E-50	-0.310	0.843	0.845	3.70E-46	0	het vs wt	3	1.44E+08	1.44E+08	2
Lmo4	1.15E-14	-0.275	0.717	0.726	2.50E-10	2	het vs wt	3	1.44E+08	1.44E+08	2
Mdk	4.96E-07	0.530	0.519	0.355	0.010828	12	het vs wt	2	91760150	91762642	2
Mdk	4.04E-19	0.459	0.971	0.901	8.82E-15	8	het vs wt	2	91760150	91762642	2
Pou3f2	9.25E-09	-0.283	0.961	0.951	0.000202	10	het vs wt	4	22482780	22488366	2
Pou3f2	1.03E-09	-0.344	0.951	0.966	2.25E-05	3	het vs wt	4	22482780	22488366	2
Ptn	5.28E-16	-0.337	0.993	0.995	1.15E-11	0	het vs wt	6	36691864	36787155	2
Ptn	4.78E-13	-0.317	0.942	0.953	1.04E-08	3	het vs wt	6	36691864	36787155	2
Rpl38	6.56E-08	-0.422	0.999	1	0.001434	11	het vs wt	11	1.15E+08	1.15E+08	2
Rpl38	1.55E-08	-0.585	0.569	0.815	0.000339	13	het vs wt	11	1.15E+08	1.15E+08	2
Rps10	9.45E-07	0.258	0.994	0.962	0.02064	12	het vs wt	17	27849392	27855643	2
Rps10	3.48E-08	-0.429	0.473	0.669	0.00076	13	het vs wt	17	27849392	27855643	2
Rsrp1	2.80E-07	-0.280	0.985	0.981	0.006107	11	het vs wt	4	1.35E+08	1.35E+08	2
Rsrp1	5.06E-12	-0.363	0.949	0.975	1.10E-07	3	het vs wt	4	1.35E+08	1.35E+08	2
Sox5	7.86E-79	0.280	0.744	0.607	1.72E-74	0	het vs wt	6	1.44E+08	1.45E+08	2
Sox5	7.24E-15	0.456	0.707	0.561	1.58E-10	11	het vs wt	6	1.44E+08	1.45E+08	2
Tmsb10	3.68E-13	-0.494	0.871	0.966	8.03E-09	13	het vs wt	6	72934330	72935731	2
Tmsb10	4.04E-07	-0.296	0.957	0.985	0.008819	5	het vs wt	6	72934330	72935731	2
Tmsb4x	2.93E-22	0.262	1	1	6.41E-18	0	het vs wt	X	1.66E+08	1.66E+08	2
Tmsb4x	3.19E-07	-0.338	0.965	0.978	0.006959	15	het vs wt	X	1.66E+08	1.66E+08	2
Tsc22d1	9.20E-08	-0.297	0.994	0.988	0.002011	11	het vs wt	14	76652401	76745205	2
Tsc22d1	1.27E-06	-0.269	0.996	0.999	0.027834	3	het vs wt	14	76652401	76745205	2
Tubb2a	5.40E-07	-0.473	0.435	0.478	0.011795	5	het vs wt	13	34258257	34261990	2
Tubb2a	1.13E-09	-0.306	0.7	0.782	2.47E-05	8	het vs wt	13	34258257	34261990	2
Vim	6.87E-11	0.344	0.779	0.652	1.50E-06	11	het vs wt	2	13578738	13587637	2

Vim	9.97E-16	0.340	0.427	0.274	2.18E-11	3	het vs wt	2	13578738	13587637	2

p_val	avg_log2FC	pct.1	pct.2	p_val_adj	cluster	Gene	analysis	chromoson	start_position	end_position
2.21E-15	-0.300	0.895	0.92	4.82E-11	0	Arpp21	het vs wt	9	111894159	112065006
2.62E-45	-0.366	0.801	0.895	5.72E-41	0	Arpp21	ko vs het	9	111894159	112065006
2.28E-189	-0.666	0.801	0.92	4.99E-185	0	Arpp21	ko vs wt	9	111894159	112065006
3.98E-33	-0.265	0.694	0.814	8.69E-29	0	Cpe	het vs wt	8	65045576	65146088
2.23E-168	-0.501	0.354	0.694	4.87E-164	0	Cpe	ko vs het	8	65045576	65146088
0	-0.767	0.354	0.814	0	0	Cpe	ko vs wt	8	65045576	65146088
2.36E-22	-0.316	0.984	0.992	5.15E-18	0	Frmd4a	het vs wt	2	4022528	4618854
1.36E-43	-0.306	0.966	0.984	2.97E-39	0	Frmd4a	ko vs het	2	4022528	4618854
2.07E-250	-0.622	0.966	0.992	4.52E-246	0	Frmd4a	ko vs wt	2	4022528	4618854
2.06E-55	-0.348	0.808	0.885	4.49E-51	0	Gatad2b	het vs wt	3	90200485	90270714
4.70E-115	-0.386	0.586	0.808	1.03E-110	0	Gatad2b	ko vs het	3	90200485	90270714
0	-0.734	0.586	0.885	0	0	Gatad2b	ko vs wt	3	90200485	90270714
6.97E-07	-0.260	0.893	0.928	0.0152184	0	Grin2b	het vs wt	6	135690231	136150509
4.71E-65	-0.433	0.772	0.893	1.03E-60	0	Grin2b	ko vs het	6	135690231	136150509
8.12E-226	-0.692	0.772	0.928	1.77E-221	0	Grin2b	ko vs wt	6	135690231	136150509
2.82E-40	0.307	1	1	6.15E-36	0	H3f3b	het vs wt	11	115912738	115918788
1.62E-146	0.323	1	1	3.53E-142	0	H3f3b	ko vs het	11	115912738	115918788
0	0.630	1	1	0	0	H3f3b	ko vs wt	11	115912738	115918788
6.29E-10	0.257	0.475	0.345	1.37E-05	0	Ifitm2	het vs wt	7	140534750	140535900
3.15E-47	0.423	0.652	0.475	6.88E-43	0	Ifitm2	ko vs het	7	140534750	140535900
1.21E-175	0.681	0.652	0.345	2.64E-171	0	Ifitm2	ko vs wt	7	140534750	140535900
8.30E-26	-0.319	0.838	0.888	1.81E-21	0	Itpr1	het vs wt	6	108190057	108528070
2.22E-118	-0.513	0.673	0.838	4.86E-114	0	Itpr1	ko vs het	6	108190057	108528070
0	-0.832	0.673	0.888	0	0	Itpr1	ko vs wt	6	108190057	108528070
4.62E-24	-0.255	0.406	0.519	1.01E-19	0	Kif26b	het vs wt	1	178356690	178766765
8.71E-117	-0.359	0.142	0.406	1.90E-112	0	Kif26b	ko vs het	1	178356690	178766765
3.43E-245	-0.614	0.142	0.519	7.49E-241	0	Kif26b	ko vs wt	1	178356690	178766765
1.69E-50	-0.310	0.843	0.845	3.70E-46	0	Lmo4	het vs wt	3	143894291	143910981
2.68E-121	-0.627	0.834	0.843	5.86E-117	0	Lmo4	ko vs het	3	143894291	143910981
1.45E-205	-0.937	0.834	0.845	3.17E-201	0	Lmo4	ko vs wt	3	143894291	143910981
1.16E-11	-0.315	0.991	0.99	2.53E-07	0	Mef2c	het vs wt	13	83652153	83815199
6.01E-57	-0.542	0.984	0.991	1.31E-52	0	Mef2c	ko vs het	13	83652153	83815199
1.91E-173	-0.857	0.984	0.99	4.18E-169	0	Mef2c	ko vs wt	13	83652153	83815199
1.16E-59	0.294	0.992	0.981	2.54E-55	0	Nfib	het vs wt	4	82208410	82623987
0	0.779	1	0.992	0	0	Nfib	ko vs het	4	82208410	82623987
0	1.073	1	0.981	0	0	Nfib	ko vs wt	4	82208410	82623987
1.79E-33	0.331	0.823	0.744	3.90E-29	0	Nrip1	het vs wt	16	76084288	76170715
1.15E-116	0.605	0.934	0.823	2.51E-112	0	Nrip1	ko vs het	16	76084288	76170715
1.58E-254	0.936	0.934	0.744	3.46E-250	0	Nrip1	ko vs wt	16	76084288	76170715
6.44E-10	-0.257	0.753	0.784	1.41E-05	0	Opcml	het vs wt	9	27702071	28836706
4.32E-21	-0.286	0.657	0.753	9.43E-17	0	Opcml	ko vs het	9	27702071	28836706
5.25E-79	-0.542	0.657	0.784	1.15E-74	0	Opcml	ko vs wt	9	27702071	28836706
5.28E-16	-0.337	0.993	0.995	1.15E-11	0	Ptn	het vs wt	6	36691864	36787155
3.92E-16	-0.323	0.99	0.993	8.57E-12	0	Ptn	ko vs het	6	36691864	36787155
7.84E-80	-0.660	0.99	0.995	1.71E-75	0	Ptn	ko vs wt	6	36691864	36787155
2.18E-53	-0.495	0.703	0.798	4.76E-49	0	Ptprk	het vs wt	10	27950816	28473393
2.70E-80	-0.479	0.555	0.703	5.89E-76	0	Ptprk	ko vs het	10	27950816	28473393
5.91E-270	-0.974	0.555	0.798	1.29E-265	0	Ptprk	ko vs wt	10	27950816	28473393
8.02E-40	0.274	0.865	0.735	1.75E-35	0	Robo1	het vs wt	16	72105194	72842983
2.65E-40	0.261	0.929	0.865	5.80E-36	0	Robo1	ko vs het	16	72105194	72842983
2.25E-169	0.535	0.929	0.735	4.91E-165	0	Robo1	ko vs wt	16	72105194	72842983
5.86E-08	-0.455	0.997	0.999	0.0012812	0	Rps28	het vs wt	17	34038001	34043536
1.85E-16	-0.269	0.997	0.997	4.04E-12	0	Rps28	ko vs het	17	34038001	34043536
0	-0.724	0.997	0.999	0	0	Rps28	ko vs wt	17	34038001	34043536
7.86E-79	0.280	0.744	0.607	1.72E-74	0	Sox5	het vs wt	6	143774151	144727703
3.03E-305	0.919	0.946	0.744	6.61E-301	0	Sox5	ko vs het	6	143774151	144727703

0	1.199	0.946	0.607	0	0	Sox5	ko vs wt		6	143774151	144727703
2.93E-22	0.262	1	1	6.41E-18	0	Tmsb4x	het vs wt	X		165990089	165992311
3.68E-42	0.269	1	1	8.04E-38	0	Tmsb4x	ko vs het	X		165990089	165992311
1.75E-221	0.531	1	1	3.82E-217	0	Tmsb4x	ko vs wt	X		165990089	165992311
1.66E-13	-0.268	0.674	0.735	3.63E-09	1	Ahi1	het vs wt		10	20828446	20956328
3.60E-23	-0.315	0.536	0.674	7.86E-19	1	Ahi1	ko vs het		10	20828446	20956328
5.66E-59	-0.583	0.536	0.735	1.24E-54	1	Ahi1	ko vs wt		10	20828446	20956328
8.77E-08	0.260	0.548	0.481	0.0019167	1	Cdkn1c	het vs wt		7	143012076	143014787
1.78E-36	0.481	0.715	0.548	3.89E-32	1	Cdkn1c	ko vs het		7	143012076	143014787
6.37E-75	0.741	0.715	0.481	1.39E-70	1	Cdkn1c	ko vs wt		7	143012076	143014787
1.78E-10	-0.312	0.949	0.961	3.89E-06	1	Celf4	het vs wt		18	25610689	25887214
1.63E-29	-0.371	0.894	0.949	3.57E-25	1	Celf4	ko vs het		18	25610689	25887214
2.11E-110	-0.683	0.894	0.961	4.61E-106	1	Celf4	ko vs wt		18	25610689	25887214
2.02E-09	-0.324	0.731	0.794	4.40E-05	1	Chl1	het vs wt		6	103487547	103727172
1.88E-40	-0.629	0.541	0.731	4.11E-36	1	Chl1	ko vs het		6	103487547	103727172
1.59E-98	-0.952	0.541	0.794	3.48E-94	1	Chl1	ko vs wt		6	103487547	103727172
3.75E-07	-0.275	0.814	0.862	0.0081863	1	Dlgap1	het vs wt		17	70276068	71128408
7.10E-51	-0.571	0.604	0.814	1.55E-46	1	Dlgap1	ko vs het		17	70276068	71128408
1.03E-116	-0.846	0.604	0.862	2.25E-112	1	Dlgap1	ko vs wt		17	70276068	71128408
4.67E-11	-0.288	0.757	0.815	1.02E-06	1	Gria1	het vs wt		11	56902213	57221070
1.59E-31	-0.474	0.616	0.757	3.48E-27	1	Gria1	ko vs het		11	56902213	57221070
1.73E-78	-0.761	0.616	0.815	3.78E-74	1	Gria1	ko vs wt		11	56902213	57221070
1.51E-15	0.381	0.999	0.998	3.31E-11	1	H3f3b	het vs wt		11	115912738	115918788
3.67E-34	0.293	1	0.999	8.02E-30	1	H3f3b	ko vs het		11	115912738	115918788
1.21E-254	0.674	1	0.998	2.65E-250	1	H3f3b	ko vs wt		11	115912738	115918788
9.29E-09	-0.416	0.506	0.619	0.0002031	1	Nrxn1	het vs wt		17	90341059	91400499
7.41E-16	-0.352	0.352	0.506	1.62E-11	1	Nrxn1	ko vs het		17	90341059	91400499
5.59E-73	-0.768	0.352	0.619	1.22E-68	1	Nrxn1	ko vs wt		17	90341059	91400499
1.78E-19	-0.331	0.959	0.942	3.88E-15	2	Atp5k	het vs wt		5	108581110	108582314
5.82E-18	-0.251	0.958	0.959	1.27E-13	2	Atp5k	ko vs het		5	108581110	108582314
2.07E-195	-0.582	0.958	0.942	4.52E-191	2	Atp5k	ko vs wt		5	108581110	108582314
4.50E-28	0.259	0.739	0.595	9.82E-24	2	Dpy19l1	het vs wt		9	24323072	24414436
1.94E-175	0.768	0.934	0.739	4.24E-171	2	Dpy19l1	ko vs het		9	24323072	24414436
6.91E-265	1.027	0.934	0.595	1.51E-260	2	Dpy19l1	ko vs wt		9	24323072	24414436
4.55E-14	-0.278	0.185	0.272	9.93E-10	2	Etv1	het vs wt		12	38829379	38920483
1.74E-41	-0.319	0.053	0.185	3.80E-37	2	Etv1	ko vs het		12	38829379	38920483
2.49E-83	-0.597	0.053	0.272	5.44E-79	2	Etv1	ko vs wt		12	38829379	38920483
1.48E-31	-0.331	0.744	0.808	3.23E-27	2	Gatad2b	het vs wt		3	90200485	90270714
6.31E-75	-0.407	0.536	0.744	1.38E-70	2	Gatad2b	ko vs het		3	90200485	90270714
3.37E-219	-0.738	0.536	0.808	7.36E-215	2	Gatad2b	ko vs wt		3	90200485	90270714
1.30E-06	-0.269	0.984	0.974	0.0283189	2	Grin2b	het vs wt		6	135690231	136150509
2.79E-15	-0.262	0.987	0.984	6.09E-11	2	Grin2b	ko vs het		6	135690231	136150509
2.17E-108	-0.530	0.987	0.974	4.74E-104	2	Grin2b	ko vs wt		6	135690231	136150509
5.20E-31	0.327	0.998	0.992	1.14E-26	2	H3f3b	het vs wt		11	115912738	115918788
3.88E-93	0.362	0.999	0.998	8.49E-89	2	H3f3b	ko vs het		11	115912738	115918788
0	0.690	0.999	0.992	0	2	H3f3b	ko vs wt		11	115912738	115918788
5.34E-14	0.302	0.524	0.352	1.17E-09	2	Jun	het vs wt		4	94937271	94940459
1.17E-43	0.479	0.723	0.524	2.55E-39	2	Jun	ko vs het		4	94937271	94940459
4.27E-146	0.781	0.723	0.352	9.34E-142	2	Jun	ko vs wt		4	94937271	94940459
9.61E-17	0.295	0.948	0.878	2.10E-12	2	Jund	het vs wt		8	71151599	71153265
5.23E-33	0.306	0.98	0.948	1.14E-28	2	Jund	ko vs het		8	71151599	71153265
8.72E-142	0.602	0.98	0.878	1.90E-137	2	Jund	ko vs wt		8	71151599	71153265
1.15E-14	-0.275	0.717	0.726	2.50E-10	2	Lmo4	het vs wt		3	143894291	143910981
4.36E-32	-0.387	0.637	0.717	9.53E-28	2	Lmo4	ko vs het		3	143894291	143910981
1.15E-66	-0.662	0.637	0.726	2.51E-62	2	Lmo4	ko vs wt		3	143894291	143910981
1.41E-16	0.446	0.946	0.878	3.08E-12	2	Nfia	het vs wt		4	97660971	98007111
2.00E-47	0.497	0.979	0.946	4.37E-43	2	Nfia	ko vs het		4	97660971	98007111

4.63E-173	0.944	0.979	0.878	1.01E-168	2	Nfia	ko vs wt	4	97660971	98007111
1.01E-07	-0.265	0.395	0.438	0.0021987	2	Nrp1	het vs wt	8	129085085	129229844
1.58E-41	-0.417	0.212	0.395	3.46E-37	2	Nrp1	ko vs het	8	129085085	129229844
6.04E-78	-0.682	0.212	0.438	1.32E-73	2	Nrp1	ko vs wt	8	129085085	129229844
5.66E-09	-0.256	0.304	0.36	0.0001237	2	Pcdh17	het vs wt	14	84681003	84776442
3.07E-24	-0.259	0.174	0.304	6.70E-20	2	Pcdh17	ko vs het	14	84681003	84776442
8.96E-54	-0.516	0.174	0.36	1.96E-49	2	Pcdh17	ko vs wt	14	84681003	84776442
1.69E-17	-0.290	0.424	0.532	3.69E-13	3	Arpp21	het vs wt	9	111894159	112065006
6.79E-30	-0.263	0.314	0.424	1.48E-25	3	Arpp21	ko vs het	9	111894159	112065006
5.88E-80	-0.553	0.314	0.532	1.28E-75	3	Arpp21	ko vs wt	9	111894159	112065006
3.65E-23	0.344	0.764	0.581	7.98E-19	3	Bcl11b	het vs wt	12	107876662	107969861
4.07E-83	0.515	0.933	0.764	8.90E-79	3	Bcl11b	ko vs het	12	107876662	107969861
1.02E-202	0.860	0.933	0.581	2.23E-198	3	Bcl11b	ko vs wt	12	107876662	107969861
1.24E-21	-0.361	0.721	0.837	2.70E-17	3	Gatad2b	het vs wt	3	90200485	90270714
8.60E-49	-0.331	0.597	0.721	1.88E-44	3	Gatad2b	ko vs het	3	90200485	90270714
4.60E-206	-0.692	0.597	0.837	1.01E-201	3	Gatad2b	ko vs wt	3	90200485	90270714
3.80E-07	-0.281	0.536	0.609	0.008306	3	Hist1h2ap	het vs wt			
1.74E-16	-0.254	0.44	0.536	3.81E-12	3	Hist1h2ap	ko vs het			
2.30E-68	-0.535	0.44	0.609	5.02E-64	3	Hist1h2ap	ko vs wt			
7.71E-13	-0.311	0.643	0.712	1.68E-08	3	Lsamp	het vs wt	16	39804723	42002042
6.53E-33	-0.398	0.513	0.643	1.43E-28	3	Lsamp	ko vs het	16	39804723	42002042
5.83E-85	-0.709	0.513	0.712	1.27E-80	3	Lsamp	ko vs wt	16	39804723	42002042
7.37E-23	-0.364	0.898	0.944	1.61E-18	3	Mapt	het vs wt	11	104122216	104222916
1.36E-52	-0.487	0.849	0.898	2.97E-48	3	Mapt	ko vs het	11	104122216	104222916
4.20E-160	-0.851	0.849	0.944	9.17E-156	3	Mapt	ko vs wt	11	104122216	104222916
2.96E-20	0.524	0.929	0.849	6.46E-16	3	Nfia	het vs wt	4	97660971	98007111
8.05E-42	0.388	0.978	0.929	1.76E-37	3	Nfia	ko vs het	4	97660971	98007111
6.17E-193	0.912	0.978	0.849	1.35E-188	3	Nfia	ko vs wt	4	97660971	98007111
4.78E-13	-0.317	0.942	0.953	1.04E-08	3	Ptn	het vs wt	6	36691864	36787155
3.88E-14	-0.311	0.92	0.942	8.47E-10	3	Ptn	ko vs het	6	36691864	36787155
4.85E-46	-0.628	0.92	0.953	1.06E-41	3	Ptn	ko vs wt	6	36691864	36787155
1.74E-10	-0.319	0.773	0.842	3.81E-06	3	Shtn1	het vs wt	19	58961788	59064532
8.58E-40	-0.413	0.639	0.773	1.87E-35	3	Shtn1	ko vs het	19	58961788	59064532
6.92E-122	-0.732	0.639	0.842	1.51E-117	3	Shtn1	ko vs wt	19	58961788	59064532
2.24E-11	-0.290	0.988	0.982	4.88E-07	4	Eif1b	het vs wt	9	120321298	120324396
3.03E-12	-0.356	0.987	0.988	6.63E-08	4	Eif1b	ko vs het	9	120321298	120324396
1.40E-26	-0.646	0.987	0.982	3.05E-22	4	Eif1b	ko vs wt	9	120321298	120324396
3.03E-18	-0.323	0.662	0.76	6.62E-14	4	Gatad2b	het vs wt	3	90200485	90270714
1.41E-32	-0.332	0.495	0.662	3.08E-28	4	Gatad2b	ko vs het	3	90200485	90270714
1.44E-129	-0.654	0.495	0.76	3.16E-125	4	Gatad2b	ko vs wt	3	90200485	90270714
1.36E-10	0.372	0.577	0.473	2.97E-06	4	Nfia	het vs wt	4	97660971	98007111
1.05E-30	0.505	0.739	0.577	2.29E-26	4	Nfia	ko vs het	4	97660971	98007111
1.89E-80	0.876	0.739	0.473	4.14E-76	4	Nfia	ko vs wt	4	97660971	98007111
9.86E-07	-0.450	0.994	0.997	0.0215315	4	Rps28	het vs wt	17	34038001	34043536
3.87E-08	-0.262	0.994	0.994	0.0008456	4	Rps28	ko vs het	17	34038001	34043536
7.50E-305	-0.712	0.994	0.997	1.64E-300	4	Rps28	ko vs wt	17	34038001	34043536
2.50E-07	-0.415	0.255	0.341	0.0054674	7	Dlgap1	het vs wt	17	70276068	71128408
7.10E-07	-0.279	0.153	0.255	0.0155195	7	Dlgap1	ko vs het	17	70276068	71128408
1.27E-21	-0.694	0.153	0.341	2.77E-17	7	Dlgap1	ko vs wt	17	70276068	71128408
4.68E-08	-0.575	0.441	0.5	0.0010227	8	Cspg5	het vs wt	9	110072851	110091644
6.47E-07	-0.364	0.435	0.441	0.0141317	8	Cspg5	ko vs het	9	110072851	110091644
9.01E-31	-0.939	0.435	0.5	1.97E-26	8	Cspg5	ko vs wt	9	110072851	110091644
4.51E-09	-0.258	0.747	0.803	9.84E-05	8	Gatad2b	het vs wt	3	90200485	90270714
7.93E-23	-0.285	0.6	0.747	1.73E-18	8	Gatad2b	ko vs het	3	90200485	90270714
3.57E-72	-0.542	0.6	0.803	7.81E-68	8	Gatad2b	ko vs wt	3	90200485	90270714
6.55E-07	-0.476	0.196	0.303	0.0143178	8	Luzp2	het vs wt	7	54485246	54918633
5.81E-12	-0.462	0.114	0.196	1.27E-07	8	Luzp2	ko vs het	7	54485246	54918633

6.93E-36	-0.938	0.114	0.303	1.51E-31	8	Luzp2	ko vs wt	7	54485246	54918633
4.04E-19	0.459	0.971	0.901	8.82E-15	8	Mdk	het vs wt	2	91760150	91762642
2.59E-14	0.280	0.97	0.971	5.66E-10	8	Mdk	ko vs het	2	91760150	91762642
2.01E-83	0.740	0.97	0.901	4.38E-79	8	Mdk	ko vs wt	2	91760150	91762642
6.15E-08	0.486	0.985	0.969	0.0013446	9	H3f3b	het vs wt	11	115912738	115918788
2.37E-09	0.333	0.993	0.985	5.17E-05	9	H3f3b	ko vs het	11	115912738	115918788
2.31E-96	0.818	0.993	0.969	5.05E-92	9	H3f3b	ko vs wt	11	115912738	115918788
1.15E-06	0.397	0.934	0.903	0.0250829	10	Ckb	het vs wt	12	111635795	111638772
3.58E-10	0.368	0.965	0.934	7.81E-06	10	Ckb	ko vs het	12	111635795	111638772
2.67E-40	0.764	0.965	0.903	5.84E-36	10	Ckb	ko vs wt	12	111635795	111638772
4.32E-09	-0.335	0.705	0.779	9.45E-05	10	Gm17750	het vs wt	13	84173416	84212922
5.41E-27	-0.471	0.567	0.705	1.18E-22	10	Gm17750	ko vs het	13	84173416	84212922
4.40E-53	-0.806	0.567	0.779	9.61E-49	10	Gm17750	ko vs wt	13	84173416	84212922
6.00E-12	0.326	0.787	0.676	1.31E-07	10	Igfbp2	het vs wt	1	72863662	72891633
7.66E-43	0.500	0.929	0.787	1.67E-38	10	Igfbp2	ko vs het	1	72863662	72891633
1.57E-85	0.827	0.929	0.676	3.43E-81	10	Igfbp2	ko vs wt	1	72863662	72891633
5.25E-12	0.290	0.95	0.853	1.15E-07	11	Edil3	het vs wt	13	88969591	89471342
6.93E-22	0.326	0.984	0.95	1.51E-17	11	Edil3	ko vs het	13	88969591	89471342
1.68E-60	0.616	0.984	0.853	3.67E-56	11	Edil3	ko vs wt	13	88969591	89471342
4.11E-14	-0.340	0.834	0.909	8.98E-10	11	Gatad2b	het vs wt	3	90200485	90270714
9.01E-28	-0.391	0.61	0.834	1.97E-23	11	Gatad2b	ko vs het	3	90200485	90270714
2.51E-97	-0.731	0.61	0.909	5.49E-93	11	Gatad2b	ko vs wt	3	90200485	90270714
2.28E-08	0.439	0.785	0.609	0.0004973	11	Ifitm2	het vs wt	7	140534750	140535900
2.14E-21	0.424	0.934	0.785	4.68E-17	11	Ifitm2	ko vs het	7	140534750	140535900
6.74E-75	0.864	0.934	0.609	1.47E-70	11	Ifitm2	ko vs wt	7	140534750	140535900
4.71E-11	-0.402	0.786	0.815	1.03E-06	11	Pantr1	het vs wt	1	42629754	42734584
5.08E-18	-0.507	0.704	0.786	1.11E-13	11	Pantr1	ko vs het	1	42629754	42734584
2.63E-35	-0.909	0.704	0.815	5.74E-31	11	Pantr1	ko vs wt	1	42629754	42734584
6.56E-08	-0.422	0.999	1	0.0014339	11	Rpl38	het vs wt	11	114559350	114563157
2.08E-09	-0.264	1	0.999	4.55E-05	11	Rpl38	ko vs het	11	114559350	114563157
3.22E-178	-0.686	1	1	7.03E-174	11	Rpl38	ko vs wt	11	114559350	114563157
1.13E-12	-0.468	0.999	0.999	2.47E-08	11	Rps28	het vs wt	17	34038001	34043536
1.77E-07	-0.256	1	0.999	0.0038597	11	Rps28	ko vs het	17	34038001	34043536
3.13E-184	-0.724	1	0.999	6.84E-180	11	Rps28	ko vs wt	17	34038001	34043536
7.24E-15	0.456	0.707	0.561	1.58E-10	11	Sox5	het vs wt	6	143774151	144727703
9.12E-23	0.617	0.859	0.707	1.99E-18	11	Sox5	ko vs het	6	143774151	144727703
1.62E-50	1.072	0.859	0.561	3.54E-46	11	Sox5	ko vs wt	6	143774151	144727703
4.96E-07	0.530	0.519	0.355	0.0108276	12	Mdk	het vs wt	2	91760150	91762642
2.27E-07	0.498	0.589	0.519	0.004967	12	Mdk	ko vs het	2	91760150	91762642
7.01E-24	1.027	0.589	0.355	1.53E-19	12	Mdk	ko vs wt	2	91760150	91762642
1.23E-06	-0.311	0.817	0.885	0.0268368	16	Gatad2b	het vs wt	3	90200485	90270714
2.22E-09	-0.436	0.603	0.817	4.84E-05	16	Gatad2b	ko vs het	3	90200485	90270714
2.05E-19	-0.746	0.603	0.885	4.47E-15	16	Gatad2b	ko vs wt	3	90200485	90270714

cluster_Condition	cells.Ctip2_Satb2	cells.n.co-express.	total	percentage_per_cluster	P value (vs WT)
0__WT	Co_expressed_Ctip2_Satb2	1371	4314	31.78	
0__Het	Co_expressed_Ctip2_Satb2	1568	4127	37.99	0.047
0__KO	Co_expressed_Ctip2_Satb2	1913	2393	79.94	0.0067
1__WT	Co_expressed_Ctip2_Satb2	183	2199	8.32	
1__Het	Co_expressed_Ctip2_Satb2	134	2677	5.01	NS
1__KO	Co_expressed_Ctip2_Satb2	62	1592	3.89	NS
2__WT	Co_expressed_Ctip2_Satb2	922	1995	46.22	
2__Het	Co_expressed_Ctip2_Satb2	1390	2633	52.79	0.041
2__KO	Co_expressed_Ctip2_Satb2	1084	1766	61.38	0.008
3__WT	Co_expressed_Ctip2_Satb2	952	1919	49.61	
3__Het	Co_expressed_Ctip2_Satb2	1743	2674	65.18	0.033
3__KO	Co_expressed_Ctip2_Satb2	1701	2017	84.33	0.001
4__WT	Co_expressed_Ctip2_Satb2	609	1836	33.17	
4__Het	Co_expressed_Ctip2_Satb2	697	2137	32.62	NS
4__KO	Co_expressed_Ctip2_Satb2	566	1611	35.13	NS
5__WT	Co_expressed_Ctip2_Satb2	65	1652	3.93	
5__Het	Co_expressed_Ctip2_Satb2	59	928	6.36	0.053
5__KO	Co_expressed_Ctip2_Satb2	37	334	11.08	0.0024
6__WT	Co_expressed_Ctip2_Satb2	100	1085	9.22	
6__Het	Co_expressed_Ctip2_Satb2	58	989	5.86	NS
6__KO	Co_expressed_Ctip2_Satb2	27	641	4.21	NS
7__WT	Co_expressed_Ctip2_Satb2	57	912	6.25	
7__Het	Co_expressed_Ctip2_Satb2	48	1019	4.71	NS
7__KO	Co_expressed_Ctip2_Satb2	24	665	3.61	NS
8__WT	Co_expressed_Ctip2_Satb2	103	964	10.68	
8__Het	Co_expressed_Ctip2_Satb2	91	1388	6.56	NS
8__KO	Co_expressed_Ctip2_Satb2	82	1250	6.56	NS
9__WT	Co_expressed_Ctip2_Satb2	93	851	10.93	
9__Het	Co_expressed_Ctip2_Satb2	43	931	4.62	NS
9__KO	Co_expressed_Ctip2_Satb2	27	582	4.64	NS
10__WT	Co_expressed_Ctip2_Satb2	140	720	19.44	
10__Het	Co_expressed_Ctip2_Satb2	194	1042	18.62	NS
10__KO	Co_expressed_Ctip2_Satb2	221	960	23.02	NS
11__WT	Co_expressed_Ctip2_Satb2	278	672	41.37	
11__Het	Co_expressed_Ctip2_Satb2	350	887	39.46	NS
11__KO	Co_expressed_Ctip2_Satb2	276	608	45.39	NS
12__WT	Co_expressed_Ctip2_Satb2	46	665	6.92	
12__Het	Co_expressed_Ctip2_Satb2	34	856	3.97	NS
12__KO	Co_expressed_Ctip2_Satb2	32	635	5.04	NS
13__WT	Co_expressed_Ctip2_Satb2	97	1079	8.99	
13__Het	Co_expressed_Ctip2_Satb2	31	603	5.14	NS
13__KO	Co_expressed_Ctip2_Satb2	27	328	8.23	NS
14__WT	Co_expressed_Ctip2_Satb2	20	266	7.52	
14__Het	Co_expressed_Ctip2_Satb2	14	262	5.34	NS
14__KO	Co_expressed_Ctip2_Satb2	12	250	4.80	NS

15__WT	Co_expressed_Ctip2_Satb2	13	450	2.89	
15__Het	Co_expressed_Ctip2_Satb2	5	172	2.91	NS
15__KO	Co_expressed_Ctip2_Satb3	5	172	2.91	NS
16__WT	Co_expressed_Ctip2_Satb2	16	243	6.58	
16__Het	Co_expressed_Ctip2_Satb2	10	263	3.80	NS
16__KO	Co_expressed_Ctip2_Satb2	5	116	4.31	NS
17__WT	Co_expressed_Ctip2_Satb2	9	189	4.76	
17__Het	Co_expressed_Ctip2_Satb2	6	228	2.63	NS
17__KO	Co_expressed_Ctip2_Satb2	3	115	2.61	NS
18__WT	Co_expressed_Ctip2_Satb2	6	206	2.91	
18__Het	Co_expressed_Ctip2_Satb2	6	149	4.03	NS
18__KO	Co_expressed_Ctip2_Satb3	9	182	4.95	NS
19__WT	Co_expressed_Ctip2_Satb2	7	130	5.38	
19__Het	Co_expressed_Ctip2_Satb2	4	114	3.51	NS
19__KO	Co_expressed_Ctip2_Satb2	1	69	1.45	NS
